# Global Prevalence and Risk Factors of Gastro-oesophageal Reflux Disease (GORD): Systematic Review with Meta-analysis

**DOI:** 10.1038/s41598-020-62795-1

**Published:** 2020-04-02

**Authors:** Jorabar Singh Nirwan, Syed Shahzad Hasan, Zaheer-Ud-Din Babar, Barbara R. Conway, Muhammad Usman Ghori

**Affiliations:** Department of Pharmacy, School of Applied Sciences, University of Huddersfield, HD1 3DH Huddersfield, UK

**Keywords:** Gastrointestinal diseases, Gastro-oesophageal reflux disease

## Abstract

Although gastro-oesophageal reflux disease (GORD) is a common medical complaint, there is currently no consensus on the global prevalence of GORD. The aim of this study was to conduct a systematic review and meta-analysis on GORD prevalence and risk factors at a global level. MEDLINE, EMBASE, CINAHL, Scopus, Cochrane library, and Google Scholar were systematically searched, without language restrictions, for studies on the prevalence and risk factors of GORD. Data were pooled using a random effects model (95% confidence interval), and the odds ratio and relative risk for each risk factor were calculated. Out of 34,355 search results, 96 records reporting the results from 102 studies fulfilled the inclusion criteria, representing 37 countries and all regions of the UN geoscheme. The global pooled prevalence of GORD was 13.98% and varied greatly according to region (12.88% in Latin America and the Caribbean to 19.55% in North America) and country (4.16% in China to 22.40% in Turkey). Using the United Nations 2017 Revision of World Population Prospects, the estimated number of individuals suffering from GORD globally is 1.03 billion. Multiple risk factors associated with a significant increase in the risk of GORD were also identified. This systematic review and meta-analysis revealed that although a substantial proportion (13.98%) of the global population suffers from GORD, there are significant variations between regions and countries. Risk factors for GORD were also identified which may allow clinicians to recognise individuals most at risk.

## Introduction

Gastro-oesophageal reflux disease (GORD) is one of the most common complaints in general medical practice and can be a debilitating condition requiring life-long medication, invasive surgery, and lifestyle changes^1,2^. It is widely accepted that the pathophysiology of GORD is multifactorial representing different ends of a spectrum varying with the severity of reflux rather than distinct pathophysiological mechanisms^3^. A number of factors have been suggested to cause GORD including an increased compliance of the oesophagogastric junction (OGJ), and a higher pressure gradient across the OGJ^4^. Additionally, differences in the meal distribution or the localisation of the acid pocket on top of the meal, as well as a hypotensive lower oesophageal sphincter (LOS), and a defective gastric sling/clasp muscle fibre component, may also lead to the occurrence of GORD symptoms^5,6^.

In addition to having significant undesirable effects on an individual’s health-related quality of life, GORD has also shown to have a significant economic and societal burden. Although recent studies regarding the economic evaluation of GORD are scarce, previous studies have estimated that the resource implications of GORD are approximately £760 million/year in the UK, whereas in the USA, the cost of health care and lost productivity due to GORD are an estimated $24 billion/year^7–11^. Furthermore, it has been estimated that the total direct costs (physician visit, costs of drugs, costs of tests, and hospitalisation) and indirect costs (number of days with total productivity loss and number of days with at least 30% lower functionality) of GORD per patient in Iran during 6 months are $97.90 purchasing power parity dollars (PPP) and $13.70 PPP respectively^12^. Overall, these statistics highlight the need for this condition to receive more global awareness.

Although a number of systematic reviews investigating the prevalence of GORD according to specific regions or countries have been conducted, evaluations of epidemiological studies from around the world are limited and have been a challenge for investigators due to language and cultural differences in symptom interpretation^13–17^. A recent systematic review and meta-analysis conducted by Eusebi *et al*. 2017 included 108 studies and assessed the global prevalence of, and risk factors for, gastric reflux symptoms rather the prevalence of GORD^18^. The aim of this review, to evaluate the global prevalence of reflux symptoms, was met. However, as these symptoms may be an indication of other gastric conditions such as irritable bowel syndrome, the global prevalence of GORD was not estimated.

More relevantly to the current review, in 2005, Dent *et al*. conducted a systematic review to evaluate the global prevalence of GORD using stringent selection criteria and a GORD definition of at least weekly heartburn and/or acid regurgitation, or diagnosed by a physician^19^. This review was updated by El-Serag *et al*. in 2013 and, in total, only included 28 studies^20^. Furthermore, their review excluded studies not published in English and did not identify any studies from Africa. Risk factors for GORD were also not thoroughly explored; however, the authors stated that this was not the primary goal of their review. Hence, to the best of our knowledge, the current literature is missing a recent comprehensive global systematic review on the prevalence of GORD with associated meta-analyses since the review conducted by El-Serag *et al*. in 2013, and devoid of an extensive global scale systematic review on the risk factors for GORD. Therefore, the aim of the current review was to search the literature systematically using PRISMA (Preferred Reporting Items for Systematic Reviews and Meta-Analyses) 2009 guidelines and estimate the global prevalence of GORD, the prevalence of GORD according to geographical location and to identify risk factors associated with an increased risk of the condition^21^. It is predictable that being aware of the demographics of GORD patients and the risk factors for GORD permits clinicians to identify individuals most at risk, allowing early diagnosis and commencement of treatment, as well as highlighting areas which require more attention from researchers and clinicians.

## Methods

### Scope of review: eligibility criteria

This systematic review was performed in accordance with the PRISMA 2009 guidelines^21^. The primary investigators (JSN and MUG) screened titles and abstracts for articles reporting (a) prevalence of GORD, (b) risk factors associated with GORD, and (c) regional differences in the prevalence of GORD. Studies focusing on pathophysiology, lifestyle approaches or interventions, and evaluations of clinical data were excluded, whereas original studies (e.g. longitudinal studies, cross-sectional studies) on prevalence of GORD were included. Meta-analyses were conducted where ≥4 studies were available. The publication period was from 1^st^ January 1947 to 30^th^ June 2018 and included studies on subjects of any age group, with no language restrictions applied as studies not published in English were translated using an in-kind support from native speakers.

### Information sources

The following databases were searched: MEDLINE, EMBASE, Cumulative Index to Nursing and Allied Health Literature (CINAHL), Scopus, Cochrane library, and Google Scholar with the last update on 30^th^ June, 2018. Reference lists of articles identified in the search and relevant review articles were included and were subject to the same eligibility evaluation.

### Searching

The search strategy identified research on prevalence and risk factors of GORD. Search terms were ‘gastroesophageal reflux’, ‘GERD’, ‘GORD’, ‘heartburn’, ‘esophagitis’ or ‘oesophagitis’ combined with ‘epidemiology’, ‘epidemiological’, ‘prevalence’, ‘incidence’ or ‘population’ in the title, abstract or list of medical subject heading terms. Titles and abstracts were screened to remove studies that were evidently irrelevant to the aim of the review. Systematic reviews, meta-analyses, conference presentations, and letters or correspondences were all excluded from this review. The full texts of the remaining studies were then examined to determine eligibility.

### Study selection

Two investigators (JSN and MUG) assessed abstracts independently against the following criteria: (1) studies assessing prevalence with or without risk factors of GORD; (2) original studies (longitudinal and cross-sectional) studies; (3) inclusion of any age group; and (4) studies which recruited more than 50 participants. Full papers of potential studies were independently assessed by the two investigators for their suitability.

### Data extraction process

Studies were catalogued according to the selected criteria and data was extracted to a Microsoft Excel^®^ 2017 spreadsheet. Data extracted from eligible studies included: response rate, continent, country and geographical location of study, sample size, age range of sample population, sample size according to age group, sample size according to gender, method of data collection, criteria used to define GORD, instrument used to collect data, duration of symptoms, overall prevalence of GORD, and prevalence of GORD according to risk factors.

### Data Items/study characteristics

#### Prevalence of GORD

GORD was defined as one or more of the following: heartburn and/or acid regurgitation at least once a week regardless of severity of symptoms, diagnosed by a clinician, according to a score-based GORD specific questionnaire, or the Montreal definition of mild symptoms occurring at least twice a week or moderate to severe symptoms occurring at least once a week. Studies reporting at least weekly prevalence of only one GORD symptom were excluded to avoid bias in the results. Study populations were required to be representative of the general population, therefore, those studies which reported the prevalence of GORD in sub-groups, such as hospital patients, employees at an institution, or patients suffering from a particular disease, were excluded.

#### Risk factors of GORD

All eligible articles were screened to identify studies that investigated any risk factors of GORD. The risk factors assessed in this review include: gender, age group, self-reported alcohol intake, BMI level, education level, marriage status, self-reported NSAIDs/aspirin use, area of domicile, self-reported smoking habits, income-level, and diet (spicy food, sweet food, meat/fish, carbonated drinks, fatty food, fried food, and coffee/tea). Low education level was defined as uneducated, primary school level only, less than high school, or 0–8 years of study; medium education level was defined as up to secondary school/high school or 9–12 years of study; and high education level was defined as college/university or ≥13 years of study. Income-level was categorised as low, medium, or high according to the criteria used by the respective study. Data regarding the prevalence of GORD according to exposure and non-exposure of risk factors was then extracted.

### Quality assessment

The quality of the papers included was rated according to the Newcastle-Ottawa Quality Assessment Scale adapted for cross-sectional studies^22^. The quality assessment results are shown in Table S1.

### Statistical analysis

All statistical analyses, apart from odds ratio (OR) and risk factor (RR), were conducted using MetaXL version 5.3. OR and RR were calculated using MedCalc online calculators^23,24^. The prevalence of GORD in each study was pooled using a random effects model to give an estimate of the global prevalence of GORD. Heterogeneity across studies was assessed using the I^2^ statistic with a cut off of 50% and the *χ*^2^ test with a *P* value of <0.10 as the threshold for statistically significant heterogeneity.

The prevalence of GORD according to the geographical location was grouped according to the United Nations (UN) geoscheme^25^. Population data for regions and countries was obtained from the UN 2017 Revision of World Population Prospects^26^, and the number of people suffering from GORD in a specific geographical location was calculated by extrapolating the pooled prevalence of GORD to the respective population data. Potential sources of heterogeneity were investigated by stratifying pooled prevalence of GORD by (a) criteria used to define GORD, (b) duration of symptoms, (c) instrument used for data collection and (d) method of data collection. The effect of risk factors on the prevalence of GORD was investigated by calculating OR and RR of risk factors, with a 95% confidence interval (CI).

## Results

### Search results

**Figure 1:**
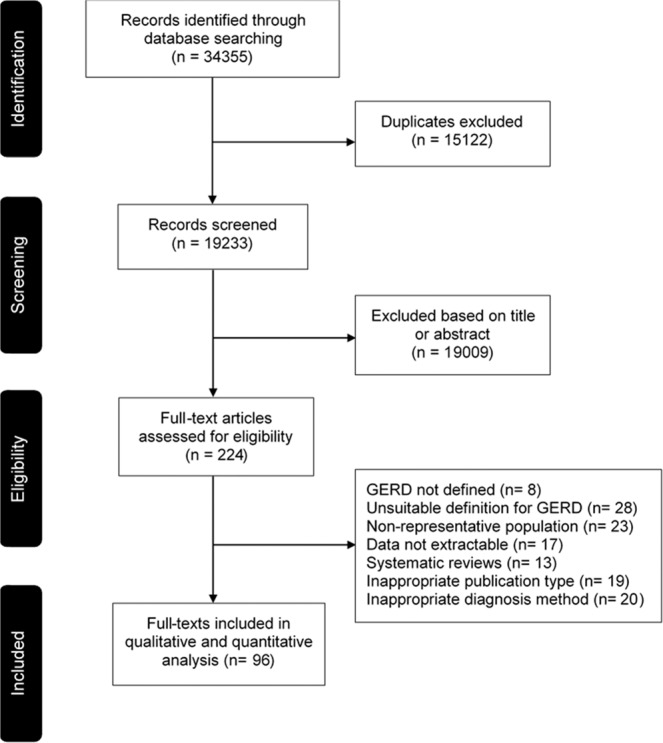
Flow-diagram of study selection.

A total of 34,355 records were retrieved from the databases after implementing the search strategy. Of these, 15,122 duplicates were excluded as well as 19,009 records excluded based on titles or abstracts. Therefore, 224 records were eligible for full-text review, of which 96 records reporting the results from 102 studies fulfilled the inclusion criteria representing 37 countries and included 469,899 participants (Fig. 1)^27–122^. The main reasons for exclusion were the use of an unsuitable definition for GORD (28 studies) and the inclusion of a non-representative population (23 studies). 5 studies were translated into English using an in-kind support of native speakers (2 from Persian and 1 each from Russian, Spanish and French). The details of the included studies are provided in Table S2.

### Prevalence of GORD according to geographical location

**Figure 2:**
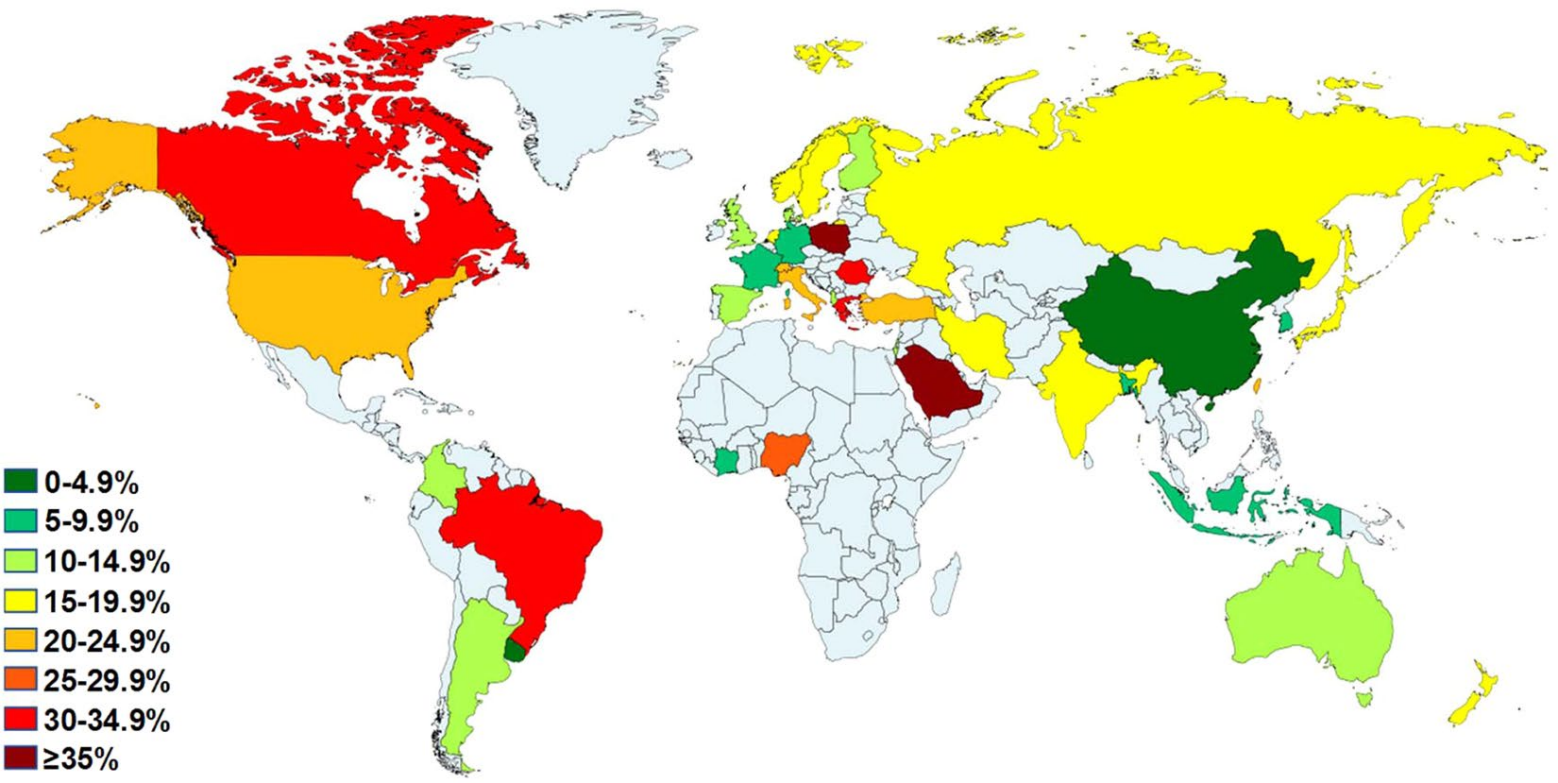
Distribution of GORD prevalence according to country.

**Figure 3:**
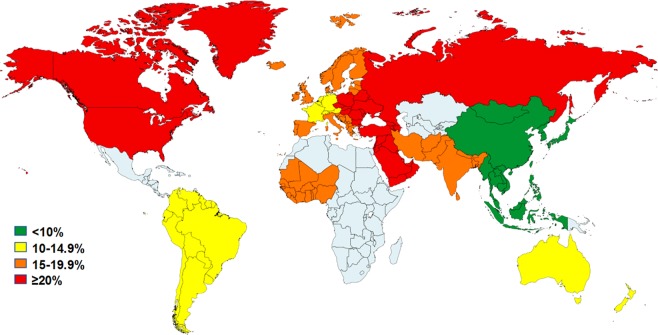
Distribution of GORD prevalence according to sub-regions (Northern America, South America, Northern Europe, Western Europe, Southern Europe, Eastern Europe, Western Africa, Western Asia, Southern Asia, Eastern Asia, South-eastern Asia, Australia & New Zealand).

**Figure 4:**
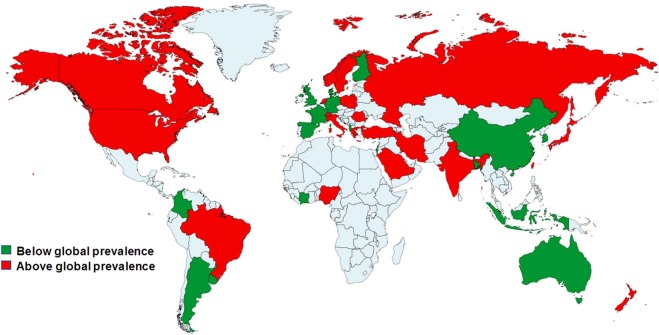
Distribution of GORD prevalence according to global prevalence.

From the 102 included studies (96 records), 63,394 subjects were diagnosed with GORD giving an overall pooled prevalence of 13.98% (95% CI 12.47%–15.56%). Using the UN 2017 Revision of World Population Prospects, the estimated number of individuals suffering from GORD globally is 1.03 billion. A funnel plot representing all of the studies included in this systematic review is depicted in Fig. S1 and also showed significant publication bias. Forest plots of the pooled prevalence of GORD stratified by country and UN region are shown in Figs. S2–S16. The distribution of the prevalence of GORD according to geographical location is shown in Table 1 and is visually depicted in Figs. 2–4. Upon visual analysis, Fig. 2 reveals substantial variations in the prevalence of GORD globally with the lowest GORD prevalence being reported in a study from China (2.5%) and the highest being reported in a study conducted in Saudi Arabia (45.4%) (Table S2)^28,79^. This was in agreement with the findings revealed in the review conducted by El-Serag *et al*. who also found the highest and lowest prevalence of GORD reported in studies from Saudi Arabia and China, respectively^20^. However, as the data was not pooled according to country, their review did not report the overall prevalence of GORD in each country. The current review identified the countries with the highest and lowest pooled prevalence of GORD as Turkey (22.40% (95% CI 18.53%–126.53%)), and China (4.16% (95% CI 3.35%–15.05%)), respectively. This also highlighted the disparity in the prevalence of GORD between sub-regions within the same continent which was especially evident in Asia which contained the sub-region with the lowest GORD prevalence (East Asia) as well as the highest GORD prevalence (Middle East) (Fig. 3). Furthermore, to highlight countries with a particularly high prevalence of GORD, Fig. 4 displays the countries with a GORD prevalence greater than and less than the global pooled GORD prevalence.

**Table 1:** Pooled prevalence of GORD according to geographical location.

Geographical location	No. of studies	No. of participants	GORD prevalence		Total estimated population size^b^	Total estimated prevalence of GORD, n^a^ (95% CI)	95% CI		I^2^	Cochran’s Q	Chi^2^, p	Tau^2^
			n^a^	%			LCI	HCI				
**Global**												
Overall	102^c^	469899	63394	13.98	7,383,008,820	1,032,144,633 (920,661,200–1,148,796,172)	12.47	15.56	99.57	23218.83	0.010	0.052
Male	50	122849	17763	15.69	3,724,132,110	584,316,328 (489,723,372–685,985,135)	13.15	18.42	99.35	7565.56	0.010	0.067
Female	50	138435	22998	17.17	3,658,876,550	628,229,104 (520,292,245–743,849,603)	14.22	20.33	99.54	10671.53	0.010	0.084
**Continents**^**b**^												
Asia	54	240451	32944	12.92	4,419,897,601	571,050,770 (464,531,238–686,410,097)	10.51	15.53	99.69	17317.65	0.010	0.078
Europe	29	190057	23833	14.12	740,813,959	104,602,931 (89,712,570–120,382,268)	12.11	16.25	99.31	4073.09	0.010	0.026
North America	9	20525	3623	19.55	356,003,541	69,598,692 (55,536,552–84,835,644)	15.60	23.83	97.89	378.91	0.010	0.024
Latin America and Caribbean	4	12756	2205	12.88	416,436,111	53,656,262 (15,949,503–106,690,932)	3.83	25.62	99.62	791.78	0.010	0.102
Oceania	4	3760	503	13.78	39,542,980	5,447,166 (4,266,688–6,746,032)	10.79	17.06	86.83	22.79	0.001	0.007
Africa^d^	2	2350	286	N/C	N/C	N/C	N/C	N/C	N/C	N/C	N/C	N/C
**Countries**												
China	10	36887	1405	4.16	1,397,028,553	58,122,991 (46,800,457–70,549,942)	3.35	5.05	92.95	127.61	0.010	0.004
Japan	7	27912	4749	13.81	127,974,958	17,673,342 (10,135,617–26,810,754)	7.92	20.95	99.57	1387.52	0.010	0.063
South Korea	7	43897	2206	5.84	50,593,662	2,953,899 (2,266,596–3,723,694)	4.48	7.36	96.65	179.08	0.010	0.006
Taiwan	1	1238	310	N/C	N/C	N/C	N/C	N/C	N/C	N/C	N/C	N/C
Indonesia	1	278	26	N/C	N/C	N/C	N/C	N/C	N/C	N/C	N/C	N/C
Bangladesh	1	2000	110	N/C	N/C	N/C	N/C	N/C	N/C	N/C	N/C	N/C
India	3	6296	955	N/C	N/C	N/C	N/C	N/C	N/C	N/C	N/C	N/C
Iran	16	102295	18323	18.43	79,360,487	14,626,138 (11,785,032–17,689,453)	14.85	22.29	99.46	2768.58	0.010	0.038
Turkey	4	13332	3356	22.40	78,271,472	17,533,182 (14,503,704–20,765,422)	18.53	26.53	95.65	68.90	0.001	0.009
Israel	2	3008	343	N/C	N/C	N/C	N/C	N/C	N/C	N/C	N/C	N/C
Saudi Arabia	2	3308	1161	N/C	N/C	N/C	N/C	N/C	N/C	N/C	N/C	N/C
Poland	1	850	302	N/C	N/C	N/C	N/C	N/C	N/C	N/C	N/C	N/C
Romania	1	184	57	N/C	N/C	N/C	N/C	N/C	N/C	N/C	N/C	N/C
Russia	2	8877	1290	N/C	N/C	N/C	N/C	N/C	N/C	N/C	N/C	N/C
Albania	1	845	101	N/C	N/C	N/C	N/C	N/C	N/C	N/C	N/C	N/C
Italy	2	2032	304	N/C	N/C	N/C	N/C	N/C	N/C	N/C	N/C	N/C
Greece	1	700	241	N/C	N/C	N/C	N/C	N/C	N/C	N/C	N/C	N/C
Spain	3	5365	640	N/C	N/C	N/C	N/C	N/C	N/C	N/C	N/C	N/C
Switzerland	2	7736	1299	N/C	N/C	N/C	N/C	N/C	N/C	N/C	N/C	N/C
Netherlands	1	502	25	N/C	N/C	N/C	N/C	N/C	N/C	N/C	N/C	N/C
France	2	46377	3206	N/C	N/C	N/C	N/C	N/C	N/C	N/C	N/C	N/C
Germany	1	268	23	N/C	N/C	N/C	N/C	N/C	N/C	N/C	N/C	N/C
Sweden	4	8120	1082	16.15	9,763,565	1,577,196 (938,279–2,338,374)	9.61	23.95	98.34	181.20	0.010	0.038
Finland	1	1700	175	N/C	N/C	N/C	N/C	N/C	N/C	N/C	N/C	N/C
Norway	1	44997	7692	N/C	N/C	N/C	N/C	N/C	N/C	N/C	N/C	N/C
UK	4	12467	1942	14.53	65,397,080	9,500,537 (5,990,373–13,635,291)	9.16	20.85	98.78	246.05	0.010	0.028
Denmark	1	48027	5387	N/C	N/C	N/C	N/C	N/C	N/C	N/C	N/C	N/C
USA	8	19489	3527	21.04	319,929,162	67,313,096 (54,132,014–81,517,950)	16.92	25.48	97.79	316.06	0.010	0.022
Canada	1	1036	96	N/C	N/C	N/C	N/C	N/C	N/C	N/C	N/C	N/C
Argentina	1	839	100	N/C	N/C	N/C	N/C	N/C	N/C	N/C	N/C	N/C
Brazil	1	3934	1231	N/C	N/C	N/C	N/C	N/C	N/C	N/C	N/C	N/C
Uruguay	1	1141	54	N/C	N/C	N/C	N/C	N/C	N/C	N/C	N/C	N/C
Colombia	1	6842	820	N/C	N/C	N/C	N/C	N/C	N/C	N/C	N/C	N/C
Australia	3	2982	381	N/C	N/C	N/C	N/C	N/C	N/C	N/C	N/C	N/C
New Zealand	1	778	122	N/C	N/C	N/C	N/C	N/C	N/C	N/C	N/C	N/C
Nigeria	1	410	108	N/C	N/C	N/C	N/C	N/C	N/C	N/C	N/C	N/C
Côte d’Ivoire	1	1940	178	N/C	N/C	N/C	N/C	N/C	N/C	N/C	N/C	N/C

GORD, gastro-oesophageal reflux disease; CI, confidence interval; LCI, lower confidence interval; HCI, higher confidence interval; N/C, not computable due to inadequate number of studies.

^a^Number of subjects with GORD.

^b^According to United Nations 2017 Revision of World Population Prospects.

^c^Results from 102 studies reported in 96 records.

^d^Inadequate number of studies to provide estimation of prevalence of GORD in Africa.

### Prevalence of GORD according to risk factors

Table 2 displays the prevalence of GORD according to risk factors. The OR and RR of risk factors for GORD are shown in Tables S3 and S4, respectively. Forest plots of the pooled prevalence of GORD stratified by risk factors are presented in Figs. S17–S34. The prevalence of GORD according to gender was reported in 50 studies. The pooled prevalence of GORD in females (17.17% (95% CI 14.22%–120.33%)) was moderately higher than in males (15.69% (95% CI 13.15%–118.42%)). The OR (1.18 (95% CI 1.15–1.20; p < 0.0001)) and RR (1.15 (95% CI 1.13–1.17; p < 0.0001)) in females compared to males showed that females are slightly more at risk of suffering from GORD.

**Table 2:** Pooled prevalence of GORD according to risk factors.

Risk factor	No. of studies	No. of participants	GORD prevalence		95% CI		I^2^	Cochran’s Q	Chi^2^, p	Tau^2^
			n^a^	%	LCI	HCI				
**Age group (years)**										
18–34	15	15065	1733	8.70	4.39	14.23	99.04	1460.27	0.010	0.112
35–59	21	55851	7362	14.53	11.09	18.33	99.24	2638.42	0.010	0.056
≥60	17	17663	2383	13.12	9.59	17.11	98.10	840.21	0.010	0.052
**Alcohol intake**										
None/Low	21	41655	4998	15.95	11.01	21.60	99.50	4028.78	0.010	0.111
Moderate/High	21	24445	3117	15.56	11.63	19.93	98.64	1470.65	0.010	0.066
**BMI**										
<18.5	6	2506	110	6.64	3.40	10.82	88.55	43.66	0.001	0.026
18.5–29.9	14	10244	1830	17.20	11.80	23.37	98.25	628.66	0.010	0.070
≥30.0	12	3423	934	22.63	17.33	28.41	92.95	156.10	0.010	0.048
**Education level**										
Low	21	24609	3582	16.78	12.32	21.77	98.83	1620.47	0.010	0.079
Medium	15	23428	1747	11.52	7.33	16.48	98.98	1375.18	0.010	0.077
High	18	16159	1433	8.98	5.56	13.09	98.46	1037.44	0.010	0.072
**Income level**										
Low	6	11034	671	11.69	4.74	20.96	98.60	357.66	0.010	0.091
Medium	4	7704	368	8.42	1.54	19.12	98.92	278.77	0.010	0.093
High	6	2615	151	7.68	3.86	12.58	92.78	69.21	0.001	0.036
**Marriage status**										
Single	12	11657	1150	12.85	6.59	20.71	98.98	1075.90	0.010	0.128
Married	12	28768	4166	15.98	10.48	22.35	99.40	1836.17	0.010	0.080
Divorced/separated/widowed	6	1538	423	22.95	13.19	34.38	95.35	107.57	0.010	0.090
**NSAIDs/aspirin use**										
Users	10	3574	741	24.47	18.17	31.35	94.38	160.09	0.010	0.054
Non-users	10	14419	2192	17.34	13.36	21.72	97.15	316.08	0.010	0.030
**Area of domicile**										
Rural	8	12387	870	11.70	6.26	18.46	98.59	495.59	0.010	0.070
Suburban	2	1376	48	N/C	N/C	N/C	N/C	N/C	N/C	N/C
Urban	8	11528	1660	13.43	6.68	21.95	99.28	969.49	0.010	0.100
**Smoking habits**										
Current smokers	28	28574	3738	18.40	14.57	22.57	98.54	1844.55	0.010	0.072
Ex-smokers	5	990	144	16.83	7.49	28.72	94.26	69.63	0.001	0.093
Non-smokers	28	54787	6921	15.55	11.63	19.91	99.43	4699.24	0.010	0.093
**Spicy food**										
Low/none	3	3397	274	N/C	N/C	N/C	N/C	N/C	N/C	N/C
Moderate/high	3	2813	280	N/C	N/C	N/C	N/C	N/C	N/C	N/C
**Sweet food**										
Low/none	2	459	136	N/C	N/C	N/C	N/C	N/C	N/C	N/C
Moderate/high	2	2177	613	N/C	N/C	N/C	N/C	N/C	N/C	N/C
**Meat/fish**										
Low/none	3	3443	313	N/C	N/C	N/C	N/C	N/C	N/C	N/C
Moderate/high	3	2234	568	N/C	N/C	N/C	N/C	N/C	N/C	N/C
**Carbonated drinks**										
Low/none	5	6837	944	14.54	6.49	24.91	99.01	403.33	0.010	0.082
Moderate/high	5	2644	452	18.60	9.55	29.68	97.51	160.71	0.010	0.081
**Fatty food**										
Low/none	2	1355	118	N/C	N/C	N/C	N/C	N/C	N/C	N/C
Moderate/high	2	395	42	N/C	N/C	N/C	N/C	N/C	N/C	N/C
**Fried food**										
Low/none	3	2799	204	N/C	N/C	N/C	N/C	N/C	N/C	N/C
Moderate/high	3	1984	378	N/C	N/C	N/C	N/C	N/C	N/C	N/C
**Coffee/tea**										
Low/none	14	7104	1018	16.92	12.69	21.61	95.62	296.68	0.010	0.045
Moderate/high	14	17174	3387	21.02	16.32	26.13	98.28	756.60	0.010	0.050

BMI, body mass index; NSAID, nonsteroidal anti-inflammatory drug; N/C, not computable due to inadequate number of studies.

^a^Number of subjects with GORD.

The pooled prevalence of GORD according to age groups displayed an increase with increasing age between the age groups of 18–34 years (8.70%; 95% CI 4.39%–114.23%) and 35–59 years (14.53% (95% CI 11.09%–118.33%)). However, there was a slight decrease between the age groups of 35–59 years and ≥60 years (13.12% (95% CI 9.59%–117.11%)). The OR (1.17 (95% CI 1.11–1.24; p < 0.0001) in those aged 35–59 years compared with those aged 18–34 years; 1.20 (95% CI 1.12–1.28; p < 0.0001) in those aged ≥60 years compared with those aged 18–34 years; 1.03 (95% CI 0.98–1.08; P = 0.2896) in those aged ≥60 years compared with those aged 35–59 years) was extremely modest. This was also the case in the RR (1.15 (95% CI 1.09–1.20; p < 0.0001) in those aged 35–59 years compared with those aged 18–34 years; 1.17 (95% CI 1.11–1.24) in those aged ≥60 years compared with those aged 18–34 years; 1.02 (95% CI 0.98–1.07; P = 0.2892) in those aged ≥60 years compared with those aged 35–59 years).

The pooled prevalence of GORD according to alcohol intake showed a similar GORD prevalence in those who do not drink alcohol or have a low intake of alcohol (15.95% (95% CI 11.01%–121.60%)) compared with those who have a moderate to high intake of alcohol (15.56% (95% CI 11.63%–119.93%)). The OR and RR of GORD in those with a moderate to high intake of alcohol compared with those who do not drink alcohol or have a low intake of alcohol was also not significantly different (OR = 1.07 (95% CI 1.02–1.12; P = 0.0044), and RR = 1.06 (95% CI 1.02–1.11; P = 0.0044)).

Stratified pooled prevalence of GORD by BMI showed an increase in GORD prevalence as BMI increased. The lowest prevalence of GORD was for those with a BMI < 18.5 (6.64% (95% CI 3.40%–110.82%)), whereas the highest prevalence of GORD was seen in those with a BMI ≥30.0 (22.63% (95% CI 17.33%–128.41%)). This positive correlation between prevalence of GORD and BMI was also shown by a significant increase in OR and RR in subjects with a higher BMI compared with subjects with a lower BMI.

The pooled prevalence of GORD according to education level was also investigated. The prevalence of GORD was highest in subjects with low education level (16.78% (95% CI 12.32%–121.77%)), followed by those with medium education level (11.52% (95% CI 7.33%–116.48%)), and the lowest GORD prevalence was seen in those with a high education level (8.98% (95% CI 5.56%–113.09%)). The impact of education level on the prevalence of GORD was also shown in the OR and RR of GORD in those with low education level compared with those with medium and high education level (OR = 2.11 (95% CI 1.99–2.24; p < 0.0001) and 1.75 (95% CI 1.64–1.87; p < 0.0001), respectively; RR = 1.95 (95% CI 1.85–2.06; p < 0.0001) and 1.64 (95% CI 1.55–1.74; p < 0.0001), respectively). However, this was not supported by the OR and RR of GORD in those with medium education level compared with those with high education level (OR = 0.82 (95% CI 0.77–0.89; p < 0.0001) and RR = 0.84 (95% CI 0.77–0.90; p < 0.0001)).

When the pooled prevalence of GORD was stratified according to marriage status, the highest prevalence of GORD was found in divorced/separated/widowed individuals (22.95% (95% CI 13.19%–134.38%)) followed by married individuals (15.98% (95% CI 10.48%–122.35%)), and the lowest GORD prevalence was seen in single individuals (12.85% (95% CI 6.59%–120.71%)). The OR and RR in divorced/separated/widowed and married individuals were also significantly greater when compared with single individuals.

The pooled prevalence of GORD according to NSAIDs/aspirin use showed a significantly greater prevalence of GORD in subjects using NSAIDs/aspirin (24.47% (95% CI 18.17%–131.35%)) compared with those who do not (17.34% (95% CI 13.36%–121.72%)). The OR and RR in those using NSAIDs/aspirin was 1.46 (95% CI 1.33–1.60; p < 0.0001) and 1.36 (95% CI 1.27–1.47; p < 0.0001), respectively.

The area of domicile also had a significant effect on the prevalence of GORD. The pooled prevalence of GORD in subjects living in an urban area was the highest (13.43% (95% CI 6.68%–121.95%)) followed by subjects living in a rural area (11.70% (95% CI 6.26%–118.46%)). The OR and RR in subjects living in an urban area compared with those living in a rural area was OR = 2.227 (95% CI 2.04–2.43; p < 0.0001) and RR = 2.05 (95% CI 1.90–2.22; p < 0.0001)). Only 2 studies reported the prevalence of GORD in individuals living in a suburban area, therefore, meta-analysis was not conducted on this sub-group (Table 2).

The pooled prevalence of GORD according to smoking habits showed that subjects who currently smoke had a higher prevalence of GORD (18.40% (95% CI 14.57%–122.57%)) compared with ex-smokers (16.83% (95% CI 7.49%–128.72%)) and non-smokers (15.55% (11.63%-19.91%)). However, the OR and RR in current smokers compared with non-smokers was insignificant (OR = 1.04 (95% CI 1.00–1.09; P = 0.0652) and RR = 1.04 (95% CI 1.00–1.08; P = 0.0650)).

Subjects with a low income had a significantly higher prevalence of GORD (11.69% (95% CI 4.74%–120.96%)) than those with a medium income (8.42% (95% CI 1.54%–119.12%)) and those with a high income (7.68% (95% CI 3.86%–112.58%)). The OR and RR of GORD in those with a low income compared with medium income level was 1.29 (95% CI 1.33–1.47) and 1.27 (95% CI 1.12–1.44), respectively. However, the OR and RR in those with a low income compared with a high income was insignificant (OR = 1.06 (95% CI 0.88–1.27); RR = 1.05 (95% CI 0.89–1.25)), and subjects with a medium income actually had low OR and RR compared with those with a high income (OR = 0.82 (95% CI 0.67–0.99); RR = 0.83 (95% CI 0.69–0.99)).

The effect of dietary intake of certain food and drinks (spicy food, sweet food, meat/fish, carbonated drinks, fatty food, fried food, and coffee/tea) on the pooled prevalence of GORD was also investigated. However, a limited number of studies have been conducted on the effects of spicy food, sweet food, meat/fish, fatty food and fried food, therefore, meta-analysis was not conducted on these sub-groups (Table 2).

Subjects with a moderate/high intake of carbonated drinks had a higher pooled prevalence of GORD than those with low/none intake (18.60% (95% CI 9.55%–129.68%) vs 14.54% (95% CI 6.49%–124.91%), respectively). The OR and RR associated with a moderate/high intake of carbonated drinks was 1.29 (95% CI 1.14–1.46; P = 0.0001) and 1.24 (95% CI 1.12–1.37; p < 0.0001), respectively.

Pooling prevalence of GORD according to intake of coffee/tea revealed a higher GORD prevalence in subjects with a moderate/high intake of coffee/tea (21.02% (95% CI 16.32%–126.13%)) than those with a low/none intake (16.92% (95% CI 12.69%–121.61%)). The OR and RR in those with a moderate/high intake of coffee/tea was 1.47 (95% CI 1.36–1.59) and 1.38 (95% CI 1.29–1.47), respectively.

### Prevalence of GORD according to study design parameters

Potential sources of heterogeneity were investigated by stratifying pooled prevalence of GORD by study design parameters including criteria used to define GORD, duration of symptoms investigated, instrument used for data collection, and method of data collection. The results of these analyses are shown in Table 3 and the forest plots of pooled prevalence of GORD stratified by study design parameters are presented in Figs. S35–S58. The majority of studies used a definition of at least weekly heartburn and/or acid regurgitation to define GORD (66 studies). From these studies, the pooled prevalence of GORD was 13.45% (95% CI 11.79%–115.20). The lowest prevalence of GORD was achieved when the Montreal definition was used (12.07% (95% CI 7.53%–117.46%)), and a definition of GERDQ score ≥8 produced the highest prevalence (17.03% (95% CI 9.24%–126.52%)).

**Table 3:** Pooled prevalence of GORD according to study design.

Study design parameter	No. of studies	No. of participants	GORD prevalence		95% CI		I^2^	Cochran’s Q	Chi^2^, p	Tau^2^
			n^a^	%	LCI	HCI				
**Criteria used to define GORD**										
At least weekly heartburn and/or acid regurgitation	66	275040	34745	13.45	11.79	15.20	99.40	11783.76	0.010	0.046
Montreal definition	10	124993	16835	12.07	7.53	17.46	99.82	4912.40	0.010	0.058
GERDQ score ≥ 7	1	278	26	N/C	N/C	N/C	N/C	N/C	N/C	N/C
GERDQ score ≥ 8	8	24929	4786	17.03	9.24	26.52	99.67	2096.59	0.010	0.106
GERDQ score ≥ 12	1	1238	310	N/C	N/C	N/C	N/C	N/C	N/C	N/C
Diagnosed by endoscopy	5	21061	2445	12.87	4.78	23.79	99.75	1606.16	0.010	0.099
Previously diagnosed by a physician	1	8143	2238	N/C	N/C	N/C	N/C	N/C	N/C	N/C
Currently undergoing treatment for GORD	3	13626	2882	N/C	N/C	N/C	N/C	N/C	N/C	N/C
FSSG score ≥ 8	1	9643	2210	N/C	N/C	N/C	N/C	N/C	N/C	N/C
FSSG score> 10	1	1130	316	N/C	N/C	N/C	N/C	N/C	N/C	N/C
QUEST score ≥ 6	2	6628	1271	N/C	N/C	N/C	N/C	N/C	N/C	N/C
RDQ score ≥ 12	1	3338	83	N/C	N/C	N/C	N/C	N/C	N/C	N/C
GORD-SMQ score> 9	1	2603	701	N/C	N/C	N/C	N/C	N/C	N/C	N/C
QUEST score ≥ 4	1	410	108	N/C	N/C	N/C	N/C	N/C	N/C	N/C
**Duration of symptoms**										
1 week	9	17558	3228	12.96	6.06	21.82	99.50	1404.15	0.010	0.109
1 month	4	22601	962	5.20	1.51	10.64	99.29	422.66	0.010	0.040
3 months	9	54860	6165	10.28	7.89	12.94	98.83	1192.54	0.010	0.026
6 months	2	20741	2152	N/C	N/C	N/C	N/C	N/C	N/C	N/C
12 months	38	171051	29199	15.23	13.25	17.32	99.17	4441.08	0.010	0.031
**Instrument used for data collection**										
GERDQ	10	26445	5122	16.97	10.14	25.09	99.58	2150.78	0.010	0.100
Mayo Reflux Questionnaire	18	78401	8643	16.96	12.95	21.39	99.54	3736.01	0.010	0.058
FSSG	2	10773	2526	N/C	N/C	N/C	N/C	N/C	N/C	N/C
DIGEST questionnaire	1	5581	443	N/C	N/C	N/C	N/C	N/C	N/C	N/C
QUEST	4	8038	1638	22.52	17.27	28.25	98.23	56.39	0.001	0.017
RDQ	4	6267	279	5.17	2.46	8.76	96.30	81.05	0.010	0.019
GHQ 28	1	1000	123	N/C	N/C	N/C	N/C	N/C	N/C	N/C
BDQ	5	7718	747	8.26	3.03	15.52	98.94	378.63	0.010	0.062
GSRS	2	2095	457	N/C	N/C	N/C	N/C	N/C	N/C	N/C
GERD-SMQ	1	2603	701	N/C	N/C	N/C	N/C	N/C	N/C	N/C
CPQ	1	672	78	N/C	N/C	N/C	N/C	N/C	N/C	N/C
SSA-P	1	1395	216	N/C	N/C	N/C	N/C	N/C	N/C	N/C
Montreal instrument	1	845	101	N/C	N/C	N/C	N/C	N/C	N/C	N/C
**Method of data collection**										
Self-completed questionnaire	22	116062	13478	13.12	9.97	16.63	99.58	5015.29	0.010	0.054
Face-to-face interview	31	204028	30148	16.98	13.74	20.49	99.74	13672.67	0.010	0.077
Telephone interview	17	101892	11612	9.57	7.80	11.49	98.77	1300.40	0.010	0.017
Postal questionnaire	20	82039	12901	15.04	12.85	17.37	98.33	1136.83	0.010	0.020
Interview-administered questionnaire	9	11376	1369	13.49	8.96	18.76	98.26	460.11	0.010	0.046
Endoscopy	5	21061	2445	12.87	4.78	23.79	99.75	1606.16	0.010	0.099

N/C, not computable due to inadequate number of studies; GerdQ, gastroesophageal reflux disease questionnaire; RDQ, Reflux Disease Questionnaire; BDQ, Bowel Disease Questionnaire; FSSG, Frequency Scale for the Symptoms of GORD; GHQ-28, General Health Questionnaire-28; GSRS, Gastrointestinal Symptom Rating Scale; DIGEST, Domestic/International Gastroenterology Surveillance Study; SSA-P, Subjective Symptom Assessment Profile; GERD-SMQ, GORD Symptom and Medication Questionnaire; CPQ, Chest Pain Questionnaire; QUEST, Quality of life and Utility Evaluation Survey Technology.

^a^Number of subjects with GORD.

The majority of studies assessed GORD symptoms during the previous 12 months (38 studies). The highest prevalence of GORD was also seen in studies which assessed GORD symptoms during the previous 12 months (15.23% (95% CI 13.25%–117.32%)), and the lowest prevalence was observed in studies which assessed GORD symptoms during the previous 1 month (5.20% (95% CI 1.51%–110.64%)).

The pooled prevalence of GORD according to the instrument used for data collection ranged from 5.17% (95% CI 2.46%–18.76%) in studies using the Reflux Disease Questionnaire (RDQ), to 26.90% (95% CI 25.24%–128.69%) in studies using the Quality of life and Utility Evaluation Survey Technology (QUEST).

The most frequently used method of data collection were face-to-face interviews (31 studies) which gave a pooled GORD prevalence of 16.98% (95% CI 13.74%–120.49%). This was also the highest prevalence of GORD when stratified by method of data collection. The lowest GORD prevalence was seen when telephone interviews were used to collect data (9.57% (95% CI 7.80%–111.49%)).

## Discussion

This comprehensive systematic review has demonstrated the significant global burden of GORD with approximately 1.03 billion individuals suffering from the condition globally. It has also confirmed substantial variations in the pooled prevalence of GORD between regions and countries. The region with the highest pooled prevalence of GORD was North America (19.55%) and the lowest pooled prevalence was in Latin America and the Caribbean (South America) (12.88%) (Fig. 3). This is in contrast with the systematic review conducted by El-Serag *et al*. 2013, which found the prevalence of GORD in South America to be significantly greater at 23.0%^20^. However, their systematic review was limited to a single study from the region. One explanation for this distribution may be the high rate of obesity in North America^123^. This systematic review has identified that obese individuals are significantly more at risk of suffering from GORD and females are also more at risk than males. The prevalence of GORD was also higher in those who had a high intake of food and drinks associated with obesity, such as fatty food and carbonated drinks, and was significantly higher in those with a moderate to high intake of coffee/tea. These factors may also explain the high prevalence of GORD in North America and Europe where intake of these foods is high (Fig. 3). Additionally, individuals living in an urban area have been identified as having a higher prevalence of GORD compared with those living in a rural area. Therefore, high levels of urbanisation may contribute to increased prevalence of GORD in Western regions, such as North America and Europe, compared to regions where rural areas predominate, such as in Asia and Latin America and the Caribbean. Moreover, individuals had significantly higher odds of suffering from GORD than married or single individuals, which may further explain the high prevalence of GORD in Europe and North America where divorce rates are high^124^.

Other factors which were associated with an increased risk of GORD were education level, age, and intake of NSAIDs/aspirin. The odds of GORD were significantly higher in those with a low level of education compared with those with a medium or high level of education. Similarly, the prevalence of GORD in individuals with a lower income was significantly greater than those with a medium income. These trends demonstrate the relationship between socioeconomic status and risk of GORD. A potential explanation for this trend may be an increased awareness of health issues in educated individuals compared with those with low or no education. Additionally, individuals with a higher income level may be able to afford a wider range of treatment options, as well as healthier food options, which may prevent or alleviate GORD symptoms. However, those with a medium level of education were not more at risk of suffering from GORD than those with a higher education level. Individuals with a medium income level were also not more at risk than those with a high-income level. Furthermore, the prevalence of GORD was higher in those aged 35–59 years than those aged 18–34 years but was slightly lower in those aged ≥60 years compared with those aged 35–59 years. GORD prevalence was also significantly higher in those who use NSAIDs/aspirin. Intake of these classes of drugs have been associated with an increase in gastric acid secretion, reduction in lower oesophageal sphincter pressure, and a delay in gastric emptying, leading to an increase in the risk of GORD^125–127^. Moreover, those with a moderate/high intake of spicy food had a significantly higher prevalence of GORD than those with low/no intake, potentially due to a slowed rate of digestion and irritation of the oesophagus caused by intake of spices^128,129^.

Interestingly, although current smokers had a higher prevalence of GORD than non-smokers and ex-smokers, the OR and RR was not significant, and was actually higher in ex-smokers compared with current smokers and non-smokers.

As one of the most comprehensive and up-to-date systematic reviews on the global prevalence and risk factors of GORD, this review has several strengths. We pooled data according to region, sub-region, and country to analyse the distribution of GORD around the world. We also identified two studies from Africa; a region from which studies have not been included in previous systematic reviews. Furthermore, we have comprehensively investigated how risk factors affect the prevalence of GORD, including more risk factors than previous systematic reviews. Articles published in a language other than English (n = 5) were translated and included in this review.

This review also has some limitations. Significant heterogeneity was demonstrated between studies in most analyses. Potential sources of heterogeneity were investigated by stratifying the pooled prevalence of GORD by study design parameters; however, significant heterogeneity was still present between studies. Substantial variations in GORD prevalence also existed within the parameters analysed. From the 102 studies included in this review, a total of 14 different definitions were used to define GORD, as well as 13 different instruments used to collect data from subjects. Although many of the instruments assessed similar symptoms, frequency of symptoms, and severity of symptoms, differences in the design may affect how subjects interpret the components of instruments. Additionally, many authors were required to translate the instruments to the language spoken in the location of interest, potentially leading to translation issues and further interpretation issues. Also, different cut-off scores were used to diagnose GORD for the same instrument e.g. for the GERDQ instrument, cut-off scores of ≥7, ≥8, and ≥12 were used by different studies, leading to a wide range in GORD prevalence for the same instrument. These factors highlight the need for studies to adhere to a consistent and official definition of GORD to allow for more reliable and comparable estimates of GORD prevalence. A potential candidate for this is the Montreal definition (mild symptoms occurring on ≥2 d of the week or moderate to severe symptoms occurring on ≥1 d of the week) which was approved by a consensus panel of 44 experts in 2006, yet over a decade later, this review identified only 10 studies using this definition^130^. Similarly, to diagnose GORD by the set definition, a standard instrument may be adopted by investigators.

In conclusion, this systematic review is the most comprehensive review conducted on the prevalence and risk factors of GORD to date, and the first to include studies from all regions of the UN geoscheme. Significant variations in GORD prevalence were found between regions and countries, and we have demonstrated that lifestyle, socioeconomic, and sociodemographic factors may contribute to these variations. These results may have long-reaching implications in clinical practice, research, and industry. The findings of this review will assist clinicians in recognising GORD symptoms in those most at risk, as well as identifying changes in lifestyle factors which may alleviate symptoms. Secondly, being aware of individuals most at risk will allow researchers to focus efforts in developing treatments more suited to high risk groups. Similarly, this will also allow governments and policy makers to target marketing campaigns to locations where prevalence of GORD is high, thereby increasing awareness of treatment options available to those with the condition.

## Supplementary information

Supplementary information.
