# Supplementary material for: Global Prevalence and Risk Factors of Gastro-oesophageal Reflux Disease (GORD): Systematic Review with Meta-analysis
Source: Sci Rep. 2020 Apr 2;10:5814. doi: 10.1038/s41598-020-62795-1 (PMC7118109; doi:10.1038/s41598-020-62795-1)
Supplement: Supplementary file 1 — Supplementary information. [file 41598_2020_62795_MOESM1_ESM.pdf]

Global Prevalence and Risk Factors of Gastro-oesophageal Reflux Disease (GORD): Systematic Review with Meta-analysis

Jorabar Singh Nirwan, Syed Shahzad Hasan, Zaheer-Ud-Din Babar,

Barbara R. Conway, Muhammad Usman Ghori *

Department of Pharmacy, School of Applied Sciences, University of Huddersfield, Huddersfield, UK HD1 3DH.

***Corresponding author:**

Dr Muhammad Usman Ghori

Email: m.ghori@hud.ac.uk
Phone: +44 (0) 1484 473295
Fax: +44 (0) 1484 472182

Table S1. Quality of studies assessment using Newcastle-Ottawa Quality Assessment Scale.

		Quality of studies			
		Selection			Outcome
Reference	Country	Question 1	Question 2	Question 3	Question 1
**East Asia**					
Cai 2015	China	*	*	*	*
Chen 2005	China	*	*	*	*
Cheung 2007	China	*	*	*	*
Guozong 2000	China	*	*		**
He 2010	China	*	*	*	*
Hu 2002	China	*	*	*	*
Ma 2009	China	*	*	*	*
Tan 2016	China	*	*	*	*
Wong 2003	China	*	*	*	*
Zou 2011	China	*	*	*	*
Fujiwara 2005	Japan	*	*	*	*
Kaji 2010	Japan	*	*		*
Kusano 2008	Japan	*	*		**
Murao 2011	Japan	*	*		**
Murase 2014	Japan	*	*		*
Niigaki 2013	Japan	*	*	*	**
Stanghellini 1999	Japan	*	*		*
Jeong 2008	Korea	*	*	*	*
Kang 2007	Korea	*	*		*
Kim 2007	Korea	*	*	*	**
Lee 2001	Korea	*	*		**
Lee 2009	Korea	*	*		*
Min 2014	Korea	*	*		*
Yang 2008	Korea	*	*		*
Hung 2011	Taiwan	*	*		*
**Southeast Asia**					
Abdullah 2016	Indonesia	*	*		*
**South Asia**					
Shaha 2012	Bangladesh	*	*		*
Bhatia 2011	India	*	*		*
Kumar 2014	India	*	*		*
Wang 2016	India	*	*	*	*
**West Asia**					
Aletaha 2007	Iran	*	*	*	*
Darvishmoghadam 2016	Iran	*	*		*
Ehsani 2007	Iran	*	*		*
Islami 2014	Iran	*	*	*	*
Khodamoradi 2017	Iran	*	*	*	*

Mansour-Ghanaei 2013	Iran	*	*		*
Mostaghni 2009	Iran	*	*	*	*
Nasseri-Moghaddam 2008	Iran	*	*	*	*
Nouraie 2007	Iran	*	*	*	*
Nouraie 2007	Iran	*	*	*	*
Pourhoseingholi 2012	Iran	*	*	*	*
Rogha 2006	Iran	*	*		*
Saberi-Firoozi 2007	Iran	*	*	*	*
Solhpour 2008	Iran				
Vossoughinia 2014	Iran	*	*	*	*
Yadegarfar 2018	Iran	*	*		*
Bor 2017	Turkey	*	*		*
Kitapcioglu 2007	Turkey	*	*	*	*
Mungan 2012	Turkey	*	*		*
Yonem 2013	Turkey	*	*		*
Moshkowitz 2011	Israel	*	*		*
Sperber 2007	Israel	*	*	*	*
Almadi 2014	Saudi Arabia	*	*		*
Alsuwat 2018	Saudi Arabia	*	*		*
**Eastern Europe**					
Ziolkowski 2012	Poland	*	*	*	*
Chirila 2016	Romania	*	*	*	*
Bor 2016	Russia	*	*		*
Lazebnik 2009	Russia	*	*	*	*
**Southern Europe**					
Cela 2013	Albania	*	*	*	*
Stanghellini 1999	Italy	*	*		*
Zagari 2008	Italy	*	*	*	*
Papatheodoridis 2005	Greece	*	*	*	*
Diaz-Rubio 2004	Spain	*	*	*	*
Ponce 2006	Spain	*	*		*
Rey 2009	Spain	*	*		*
**Western Europe**					
Schwenkglenks 2004	Switzerland	*	*	*	*
Stanghellini 1999	Switzerland	*	*		*
Stanghellini 1999	Netherlands	*	*		*
Bretagne 2006	France	*	*	*	*
Bruley Des Varannes 2006	France	*	*	*	*
Bollschweiler 2008	Germany	*	*	*	*
**Northern Europe**					
Djarv 2012	Sweden	*	*	*	*
Ronkainen 2005	Sweden	*	*	*	*
Terry 2000	Sweden	*	*		*
Wiklund 2006	Sweden	*	*	*	*

Stanghellini 1999	Nordic (Denmark, Finland, Norway, Sweden)	*	*		*
Isolauri 1995	Finland	*	*	*	*
Ness-Jensen 2011	Norway	*	*		*
Mohammad 2003	UK	*	*	*	*
Mohammed 2005	UK	*	*	*	*
Murray 2003	UK	*	*		*
Penston 1996	UK	*			*
Rasmussen 2015	Denmark	*	*	*	*
**North America**					
Crews 2016	USA	*	*		*
Dean 2010	USA	*	*		*
Farup 2001	USA	*	*	*	*
Jung 2007	USA	*	*	*	*
Locke 1997	USA	*	*	*	*
Locke 1999	USA	*	*	*	*
Stanghellini 1999	USA	*			*
Yuen 2010	USA	*	*		*
Stanghellini 1999	Canada	*	*		*
**Latin America and the Caribbean**					
Chiocca 2005	Argentina	*	*	*	*
Oliveira 2005	Brazil	*	*	*	*
Dacoll 2012	Uruguay	*	*		*
Paramo-Hernandez 2016	Colombia	*	*	*	*
**Oceania**					
Eslick 2009	Australia	*	*	*	*
Pandeya 2012	Australia	*	*	*	*
Talley 1998	Australia	*	*	*	*
Haque 2000	New Zealand	*	*	*	*
**Africa**					
Nwokediuko 2009	Nigeria	*	*		*
Lohoues-Kouacou 2013	Côte d'Ivoire	*	*	*	*

Table S2. Details of studies included in this systematic review.

Reference	Country	Sample size	Prevalence of GORD n[a]	%	Method of data collection (instrument)	GORD criteria	Duration of symptoms	Risk factors assessed
**East Asia**								
Cai 2015	China	2950	141	4.78	Self-completed questionnaire (GerdQ)	GerdQ score at least 8	Previous 1 week	Gender
Chen 2005	China	3338	83	2.49	Face-to-face interviews (RDQ)	RDQ score at least 12	Previous 1 month	Age, BMI, education, gender, marriage status, area of living
Cheung 2007	China	1649	79	4.79	Telephone interview (BDQ)	Heartburn and/or acid regurgitation at least once a week	N/A	N/A
Guozong 2000	China	4992	288	5.77	Endoscopy and self-completed questionnaire	Endoscopically proven esophagitis	Previous 12 months	N/A
He 2010	China	16078	496	3.08	Self-completed questionnaire	Montreal definition	Previous 1 month	Age, alcohol, BMI, education, gender, income level, area of living, smoking
Hu 2002	China	1649	79	4.79	Telephone interview (BDQ)	Heartburn and/or acid regurgitation at least once a week	N/A	Gender
Ma 2009	China	919	57	6.20	Self-completed questionnaire (RDQ)	Heartburn and/or acid regurgitation at least once a week	Previous 1 week	Age, alcohol, BMI, education, gender, income level, marriage status, area of living, smoking
Tan 2016	China	2074	79	3.81	Telephone interview	Montreal definition	N/A	N/A
Wong 2003	China	2209	55	2.49	Telephone interview (Mayo Reflux Questionnaire)	Heartburn and/or acid regurgitation at least once a week	Previous 12 months	Gender
Zou 2011	China	1029	48	4.66	Self-completed questionnaire (RDQ)	Montreal definition	Previous 1 month	N/A
Fujiwara 2005	Japan	6035	399	6.61	Self-completed questionnaire	Heartburn and/or acid regurgitation at least once a week	Previous 12 months	Age, gender
Kaji 2010	Japan	2680	207	7.72	Self-completed questionnaire	Heartburn and/or acid regurgitation at least once a week	Previous 3 months	N/A
Kusano 2008	Japan	2426	644	26.55	Face-to-face interviews and endoscopy	Heartburn and/or acid regurgitation at least once	N/A	N/A

						a week and/or reflux esophagitis		
Murao 2011	Japan	2853	667	23.38	Endoscopy and face-to-face interviews (QUEST)	Endoscopically proven esophagitis and/or QUEST score at least 6	N/A	Alcohol, diet, gender, smoking
Murase 2014	Japan	9643	2210	22.92	Self-completed questionnaire (FSSG)	FSSG score at least 8 and/or currently undergoing treatment for GORD	N/A	Gender
Niigaki 2013	Japan	3775	604	16.00	Endoscopy and self-completed questionnaire (QUEST)	Endoscopically proven esophagitis and/or QUEST score at least 6 and/or those currently undergoing treatment for GORD	N/A	Gender
Stanghellini 1999	Japan	500	18	3.60	Face-to-face interviews (DIGEST questionnaire)	Heartburn and/or acid regurgitation at least once a week	Previous 3 months	N/A
Jeong 2008	Korea	1417	50	3.53	Face-to-face interviews (BDQ)	Heartburn and/or acid regurgitation at least once a week	N/A	Age
Kang 2007	Korea	2442	200	8.19	Self-completed questionnaire	Heartburn and/or acid regurgitation at least once a week	Previous 12 months	Alcohol, diet, gender, smoking
Kim 2007	Korea	25536	1161	4.55	Face-to-face interviews (Mayo Reflux Questionnaire)	Heartburn and/or acid regurgitation at least once a week	N/A	N/A
Lee 2001	Korea	7015	242	3.45	Endoscopy and interview-administered questionnaire	Endoscopically proven esophagitis	N/A	Age, alcohol, smoking
Lee 2009	Korea	1443	123	8.52	Self-completed questionnaire	Heartburn and/or acid regurgitation at least once a week	Previous 12 months	N/A
Min 2014	Korea	5000	356	7.12	Telephone interview	Montreal definition	Previous 3 months	Alcohol, education, gender, marriage status, smoking
Yang 2008	Korea	1044	74	7.09	Telephone interview	Heartburn and/or acid regurgitation at least once a week	Previous 12 months	N/A
Hung 2011	Taiwan	1238	310	25.04	Self-completed questionnaire (GerdQ)	GerdQ score at least 12	N/A	Age, alcohol, BMI, gender, smoking
**Southeast Asia**								
Abdullah 2016	Indonesia	278	26	9.35	Self-completed questionnaire (GerdQ)	GerdQ score at least 7	Previous 1 week	BMI, diet, education, gender, income level, marriage status, smoking
**South Asia**								

Shaha 2012	Bangladesh	2000	110	5.50	Interview-administered questionnaire	Heartburn and/or acid regurgitation at least once a week	Previous 12 months	Age, BMI, education, gender, area of living, smoking
Bhatia 2011	India	3224	245	7.60	Postal questionnaire	Heartburn and/or acid regurgitation at least once a week	N/A	Alcohol, diet, gender, smoking
Kumar 2014	India	2000	472	23.60	Face-to-face interviews	Heartburn and/or acid regurgitation at least once a week	N/A	N/A
Wang 2016	India	1072	238	22.20	Interview-administered questionnaire (GerdQ)	GerdQ score at least 8	N/A	Age, BMI, education, gender, area of living, smoking
				**West Asia**				
Aletaha 2007	Iran	1000	123	12.30	Face-to-face interviews (GHQ 28)	Heartburn and/or acid regurgitation at least once a week	Previous 12 months	N/A
Darvishmoghadam 2016	Iran	2265	635	28.04	Face-to-face interviews (Mayo Reflux Questionnaire)	Heartburn and/or acid regurgitation at least once a week	Previous 12 months	Alcohol, BMI, diet, education, gender
Ehsani 2007	Iran	700	132	18.86	Face-to-face interviews	Heartburn and/or acid regurgitation at least once a week	N/A	N/A
Islami 2014	Iran	50001	9944	19.89	Face-to-face interviews	Montreal definition	Previous 12 months	N/A
Khodamoradi 2017	Iran	9264	2325	25.10	Face-to-face interviews	Heartburn and/or acid regurgitation at least once a week	Previous 12 months	Gender
Mansour-Ghanaei 2013	Iran	1473	134	9.10	Telephone interview (GerdQ)	GerdQ score at least 8	Previous 1 week	Age, BMI, diet, gender, NSAIDs/aspirin use, smoking
Mostaghni 2009	Iran	717	237	33.05	Face-to-face interviews	Heartburn and/or acid regurgitation at least once a week	Previous 12 months	Diet, gender, marriage status, NSAIDs/aspirin use, smoking
Nasseri-Moghaddam 2008	Iran	2057	374	18.18	Self-completed questionnaire (Mayo Reflux Questionnaire)	Heartburn and/or acid regurgitation at least once a week	Previous 12 months	Diet, education, gender, marriage status, NSAIDs/aspirin use, smoking
Nouraie 2007	Iran	1202	105	8.74	Telephone interview	Heartburn and/or acid regurgitation at least once a week	N/A	N/A
Nouraie 2007	Iran	2561	542	21.16	Face-to-face interviews	Heartburn and/or acid regurgitation at least once a week	Previous 6 months	Age, education, gender, area of living, smoking

Pourhoseingholi 2012	Iran	18180	1610	8.86	Face-to-face interviews (Mayo Reflux Questionnaire)	Heartburn and/or acid regurgitation at least once a week	Previous 6 months	Age, education, gender, marriage status
Rogha 2006	Iran	2400	600	25.00	Face-to-face interviews	Heartburn and/or acid regurgitation at least once a week	Previous 12 months	N/A
Saberi-Firoozi 2007	Iran	1978	304	15.37	Interview-administered questionnaire	Heartburn and/or acid regurgitation at least once a week	Previous 12 months	Education, area of living
Solhpour 2008	Iran	5733	522	9.11	Face-to-face interviews (Mayo Reflux Questionnaire)	Heartburn and/or acid regurgitation at least once a week	Previous 3 months	Gender
Vossoughinia 2014	Iran	1634	420	25.70	Postal questionnaire (Mayo Reflux Questionnaire)	Heartburn and/or acid regurgitation at least once a week	Previous 12 months	N/A
Yadegarfar 2018	Iran	1130	316	27.96	Face-to-face interviews (FSSG)	FSSG score > 10	N/A	N/A
Bor 2017	Turkey	3214	733	22.81	Face-to-face interviews (Mayo Reflux Questionnaire)	Heartburn and/or acid regurgitation at least once a week	N/A	Alcohol, BMI, gender, marriage status
Kitapcioglu 2007	Turkey	630	126	20.00	Face-to-face interviews (Mayo Reflux Questionnaire)	Heartburn and/or acid regurgitation at least once a week	Previous 12 months	NSAIDs/aspirin use
Mungan 2012	Turkey	8143	2238	27.48	Face-to-face interviews (GerdQ)	GerdQ score at least 8 and/or previously diagnosed by a physician	Previous 1 week	Age, gender
Yonem 2013	Turkey	1345	259	19.26	Face-to-face interviews	Montreal definition	Previous 12 months	Age, alcohol, diet, gender, income level, NSAIDs/aspirin use, smoking
Moshkowitz 2011	Israel	2027	252	12.43	Telephone interview	Heartburn and/or acid regurgitation at least once a week	N/A	Age, education, gender, income level
Sperber 2007	Israel	981	91	9.28	Telephone interview (RDQ)	Heartburn and/or acid regurgitation at least once a week	Previous 12 months	N/A
Almadi 2014	Saudi Arabia	1265	574	45.38	Face-to-face interviews (GerdQ)	GerdQ score at least 8	N/A	Gender, smoking
Alsuwat 2018	Saudi Arabia	2043	587	28.73	Self-completed questionnaire and face-to-face interviews (GerdQ)	GerdQ score at least 8	N/A	Education, gender, marriage status, area of living
**Eastern Europe**								
Ziolkowski 2012	Poland	850	302	35.53	Face-to-face interviews	Heartburn and/or acid regurgitation at least once a week	N/A	Gender
Chirila 2016	Romania	184	57	30.98	Interview-administered questionnaire	Montreal definition	N/A	Alcohol, BMI, diet, gender, smoking

Bor 2016	Russia	1065	251	23.57	Face-to-face interviews (Mayo Reflux Questionnaire)	Heartburn and/or acid regurgitation at least once a week	Previous 12 months	Alcohol, BMI, diet, education, gender, marriage status, NSAIDs/aspirin use, smoking
Lazebnik 2009	Russia	7812	1039	13.30	Telephone interview (Mayo Reflux Questionnaire)	Heartburn and/or acid regurgitation at least once a week	Previous 12 months	Age, alcohol, gender, NSAIDs/aspirin use, smoking
**Southern Europe**								
Cela 2013	Albania	845	101	11.95	Interview-administered questionnaire (Montreal instrument)	Montreal definition	Previous 12 months	Alcohol, BMI, diet, education, gender, income level, smoking
Stanghellini 1999	Italy	999	59	5.91	Face-to-face interviews (DIGEST questionnaire)	Heartburn and/or acid regurgitation at least once a week	Previous 3 months	N/A
Zagari 2008	Italy	1033	245	23.72	Interview-administered questionnaire	Heartburn and/or acid regurgitation at least once a week	Previous 12 months	Age, alcohol, BMI, diet, gender, NSAIDs/aspirin use, smoking
Papatheodoridis 2005	Greece	700	241	34.43	Face-to-face interviews (GSRS)	Heartburn and/or acid regurgitation at least once a week	Previous 1 week	N/A
Diaz-Rubio 2004	Spain	2500	245	9.80	Telephone interview (Mayo Reflux Questionnaire)	Heartburn and/or acid regurgitation at least once a week	Previous 12 months	Age, education, gender, area of living
Ponce 2006	Spain	2156	335	15.54	Postal questionnaire	Heartburn and/or acid regurgitation at least once a week	Previous 1 month	N/A
Rey 2009	Spain	709	60	8.46	Interview-administered questionnaire (Mayo Reflux Questionnaire)	Heartburn and/or acid regurgitation at least once a week	Previous 12 months	N/A
**Western Europe**								
Schwenkglenks 2004	Switzerland	7222	1274	17.64	Telephone interview	Heartburn and/or acid regurgitation at least once a week	N/A	Age, BMI, gender, smoking
Stanghellini 1999	Switzerland	514	25	4.86	Face-to-face interviews (DIGEST questionnaire)	Heartburn and/or acid regurgitation at least once a week	Previous 3 months	N/A
Stanghellini 1999	Netherlands	502	25	4.98	Face-to-face interviews (DIGEST questionnaire)	Heartburn and/or acid regurgitation at least once a week	Previous 3 months	N/A
Bretagne 2006	France	5395	419	7.77	Postal questionnaire	Heartburn and/or acid regurgitation at least once a week	N/A	N/A

Bruley Des Varannes 2006	France	40982	2787	6.80	Face-to-face interviews	Heartburn and/or acid regurgitation at least once a week	N/A	N/A
Bollschweiler 2008	Germany	268	23	8.58	Postal questionnaire	Heartburn and/or acid regurgitation at least once a week	N/A	N/A
**Northern Europe**								
Djarv 2012	Sweden	4910	472	9.61	Postal questionnaire	Heartburn and/or acid regurgitation at least once a week	N/A	N/A
Ronkainen 2005	Sweden	1000	259	25.90	Telephone interview and postal questionnaire (QUEST)	Heartburn and/or acid regurgitation at least once a week	Previous 3 months	Gender
Terry 2000	Sweden	815	135	16.56	Face-to-face interviews	Heartburn and/or acid regurgitation at least once a week	Previous 12 months	N/A
Wiklund 2006	Sweden	1395	216	15.48	Postal questionnaire (GSRS and SSA-P)	Heartburn and/or acid regurgitation at least once a week	Previous 1 week	N/A
Stanghellini 1999	Nordic (Denmark, Finland, Norway, Sweden)	1010	67	6.63	Face-to-face interviews (DIGEST questionnaire)	Heartburn and/or acid regurgitation at least once a week	Previous 3 months	N/A
Isolauri 1995	Finland	1700	175	10.29	Postal questionnaire	Heartburn and/or acid regurgitation at least once a week	Previous 1 week	N/A
Ness-Jensen 2011	Norway	44997	7692	17.09	Postal questionnaire	Heartburn and/or acid regurgitation at least once a week	Previous 12 months	Gender
Mohammad 2003	UK	3920	706	18.01	Postal questionnaire	Heartburn and/or acid regurgitation at least once a week	Previous 12 months	N/A
Mohammed 2005	UK	1533	322	21.00	Postal questionnaire	Heartburn and/or acid regurgitation at least once a week	Previous 12 months	N/A
Murray 2003	UK	4902	783	15.97	Self-completed questionnaire	Heartburn and/or acid regurgitation at least once a week	Previous 3 months	N/A
Penston 1996	UK	2112	131	6.20	Interview-administered questionnaire	Heartburn and/or acid regurgitation at least once a week	Previous 12 months	N/A
Rasmussen 2015	Denmark	48027	5387	11.22	Self-completed questionnaire and telephone interview	Montreal definition	N/A	Age, gender
**North America**								
Crews 2016	USA	205	68	33.17	Self-completed questionnaire (Mayo Reflux Questionnaire)	Heartburn and/or acid regurgitation at least once a week and/or those	Previous 12 months	N/A

						currently undergoing treatment for GORD		
Dean 2010	USA	2603	701	26.93	Self-completed questionnaire (GERD-SMQ)	GERD SMQ score higher than 9	Previous 3 months	N/A
Farup 2001	USA	9181	1284	13.99	Telephone interview	Heartburn and/or acid regurgitation at least once a week	Previous 3 months	Age, gender
Jung 2007	USA	2273	411	18.08	Postal questionnaire (BDQ)	Heartburn and/or acid regurgitation at least once a week	Previous 12 months	Alcohol, education, gender, marriage status, smoking
Locke 1997	USA	1511	299	19.79	Postal questionnaire (Mayo Reflux Questionnaire)	Heartburn and/or acid regurgitation at least once a week	Previous 12 months	N/A
Locke 1999	USA	1524	304	19.95	Postal questionnaire (Mayo Reflux Questionnaire)	Heartburn and/or acid regurgitation at least once a week	Previous 12 months	Alcohol, diet, gender, smoking
Stanghellini 1999	USA	1020	153	15.00	Face-to-face interviews (DIGEST questionnaire)	Heartburn and/or acid regurgitation at least once a week	Previous 3 months	N/A
Yuen 2010	USA	1172	307	26.19	Self-completed questionnaire	Heartburn and/or acid regurgitation at least once a week	N/A	N/A
Stanghellini 1999	Canada	1036	96	9.27	Face-to-face interviews (DIGEST questionnaire)	Heartburn and/or acid regurgitation at least once a week	Previous 3 months	N/A
**Latin America and the Caribbean**								
Chiocca 2005	Argentina	839	100	11.92	Postal questionnaire (Mayo Reflux Questionnaire)	Heartburn and/or acid regurgitation at least once a week	N/A	Gender
Oliveira 2005	Brazil	3934	1231	31.29	Face-to-face interviews	Heartburn and/or acid regurgitation at least once a week	N/A	Age, alcohol, BMI, education, gender, marriage status, smoking
Dacoll 2012	Uruguay	1141	54	4.73	Self-completed questionnaire (GerdQ)	GerdQ score at least 8	N/A	Education, gender
Paramo-Hernandez 2016	Colombia	6842	820	11.98	Telephone interview (GerdQ)	GerdQ score at least 8	N/A	N/A
**Oceania**								
Eslick 2009	Australia	672	78	11.61	Postal questionnaire (CPQ)	Heartburn and/or acid regurgitation at least once a week	Previous 12 months	N/A
Pandeya 2012	Australia	1580	175	11.08	Postal questionnaire	Heartburn and/or acid regurgitation at least once a week	Previous 12 months	Age, alcohol, BMI, diet, education, gender, NSAIDs/aspirin use, smoking

Talley 1998	Australia	730	128	17.53	Postal questionnaire (BDQ)	Heartburn and/or acid regurgitation at least once a week	Previous 12 months	N/A
Haque 2000	New Zealand	778	122	15.68	Postal questionnaire (Mayo Reflux Questionnaire)	Heartburn and/or acid regurgitation at least once a week	Previous 12 months	N/A
**Africa**								
Nwokediuko 2009	Nigeria	410	108	26.34	Self-completed questionnaire (QUEST)	Montreal definition and/or QUEST score at least 4	N/A	Alcohol, diet, gender, NSAIDs/aspirin use, smoking
Lohoues-Kouacou 2013	Côte d'Ivoire	1940	178	9.18	Face-to-face interviews	Heartburn and/or acid regurgitation at least once a week	N/A	N/A

N/A, information not available; GerdQ, gastroesophageal reflux disease questionnaire; RDQ, Reflux Disease Questionnaire; BDQ, Bowel Disease Questionnaire; FSSG, Frequency Scale for the Symptoms of GORD; GHQ-28, General Health Questionnaire-28; GSRS, Gastrointestinal Symptom Rating Scale; DIGEST, Domestic/International Gastroenterology Surveillance Study; SSA-P, Subjective Symptom Assessment Profile; GERD-SMQ, GERD Symptom and Medication Questionnaire; CPQ, Chest Pain Questionnaire; QUEST, Quality of life and Utility Evaluation Survey Technology.

[a] Number of subjects with GORD.

Table S3. Odds ratios of the risk factors for GORD.

Risk factor	Compared to	Odds ratio	95% CI	Significance level
**Gender**				
Females	Males	1.179	1.154-1.204	P < 0.0001
**Age (years)**				
35-59	18-34	1.168	1.105-1.235	P < 0.0001
≥60	18-34	1.200	1.123-1.282	P < 0.0001
≥60	35-59	1.027	0.977-1.080	P= 0.2896
**Alcohol intake**				
Moderate/High	None/Low	1.072	1.022-1.124	P= 0.0044
**BMI**				
18.5-29.9	<18.5	4.737	3.888-5.773	P < 0.0001
≥30	<18.5	5.350	4.383-6.529	P < 0.0001
≥30	18.5-29.9	1.725	1.576-1.889	P < 0.0001
**Education level**				
Low	Medium	2.114	1.991-2.246	P < 0.0001
Low	High	1.751	1.641-1.868	P < 0.0001
Medium	High	0.820	0.770-0.891	P < 0.0001
**Income level**				
Low	Medium	1.291	1.133-1.471	P= 0.0001

Low	High	1.057	0.881-1.267	P= 0.5532
Medium	High	0.819	0.674-0.995	P= 0.0440
**Marriage status**				
Married	Single	1.547	1.444-1.658	P < 0.0001
Divorced/separated/widowed	Single	3.466	3.052-3.937	P < 0.0001
Divorced/separated/widowed	Married	2.240	1.994-2.518	P < 0.0001
**NSAIDs/aspirin use**				
Users	Non-users	1.459	1.330-1.601	P < 0.0001
**Area of living**				
Urban	Rural	2.227	2.043-2.428	P < 0.0001
Rural	Suburban	2.090	1.554-2.810	P < 0.0001
Urban	Suburban	4.654	3.473-6.236	P < 0.0001
**Smoking status**				
Current smokers	Ex-smokers	0.884	0.739-1.059	P= 0.1803
Current smokers	Non-smokers	1.041	0.998-1.086	P= 0.0652
Ex-smokers	Non-smokers	1.177	0.985-1.407	P= 0.0732
**Diet**				
**Spicy food**				
Moderate/high	Low/none	1.260	1.058-1.500	P= 0.0095
**Sweet food**				
Moderate/high	Low/none	0.931	0.746-1.161	P= 0.5253

**Meat/fish**				
Moderate/high	Low/none	3.409	2.934-3.962	P < 0.0001
**Carbonated drinks**				
Moderate/high	Low/none	1.287	1.139-1.455	P= 0.0001
**Fatty food**				
Moderate/high	Low/none	1.247	0.860-1.808	P= 0.2437
**Fried food**				
Moderate/high	Low/none	2.994	2.498-3.589	P < 0.0001
**Coffee/tea**				
Moderate/high	Low/none	1.469	1.361-1.585	P < 0.0001

Table S4. Relative risks of the risk factors for GORD.

Risk factor	Compared to	Relative risk	95% CI	Significance level
**Gender**				
Females	Males	1.149	1.128-1.170	P < 0.0001
**Age (years)**				
35-59	18-34	1.146	1.091-1.204	P < 0.0001
≥60	18-34	1.173	1.107-1.243	P < 0.0001
≥60	35-59	1.024	0.981-1.069	P= 0.2892
**Alcohol intake**				
Moderate/High	None/Low	1.063	1.019-1.109	P= 0.0044
**BMI**				
18.5-29.9	<18.5	4.070	3.374-4.909	P < 0.0001
≥30	<18.5	6.216	5.137-7.523	P < 0.0001
≥30	18.5-29.9	1.527	1.426-1.636	P < 0.0001
**Education level**				
Low	Medium	1.952	1.849-2.061	P < 0.0001
Low	High	1.641	1.549-1.739	P < 0.0001
Medium	High	0.841	0.786-0.899	P < 0.0001
**Income level**				
Low	Medium	1.273	1.125-1.441	P= 0.0001
Low	High	1.053	0.887-1.250	P= 0.5536

Medium	High	0.827	0.688-0.995	P= 0.0435
**Marriage status**				
Married	Single	1.468	1.380-1.561	P < 0.0001
Divorced/separated/widowed	Single	2.788	2.528-3.075	P < 0.0001
Divorced/separated/widowed	Married	1.899	1.743-2.070	P < 0.0001
**NSAIDs/aspirin use**				
Users	Non-users	1.364	1.266-1.470	P < 0.0001
**Area of living**				
Urban	Rural	2.050	1.896-2.217	P < 0.0001
Rural	Suburban	2.013	1.514-2.678	P < 0.0001
Urban	Suburban	4.128	3.115-5.470	P < 0.0001
**Smoking status**				
Current smokers	Ex-smokers	0.899	0.771-1.049	P= 0.1769
Current smokers	Non-smokers	1.036	0.998-1.075	P= 0.0650
Ex-smokers	Non-smokers	1.151	0.989-1.341	P= 0.0701
**Diet**				
**Spicy food**				
Moderate/high	Low/none	1.234	1.053-1.447	P= 0.0095
**Sweet food**				
Moderate/high	Low/none	0.950	0.813-1.111	P= 0.5225
**Meat/fish**				

Moderate/high	Low/none	2.797	2.463-3.176	P < 0.0001
**Carbonated drinks**				
Moderate/high	Low/none	1.238	1.117-1.372	P < 0.0001
**Fatty food**				
Moderate/high	Low/none	1.221	0.874-1.705	P= 0.2411
**Fried food**				
Moderate/high	Low/none	2.614	2.227-3.069	P < 0.0001
**Coffee/tea**				
Moderate/high	Low/none	1.376	1.291-1.468	P < 0.0001

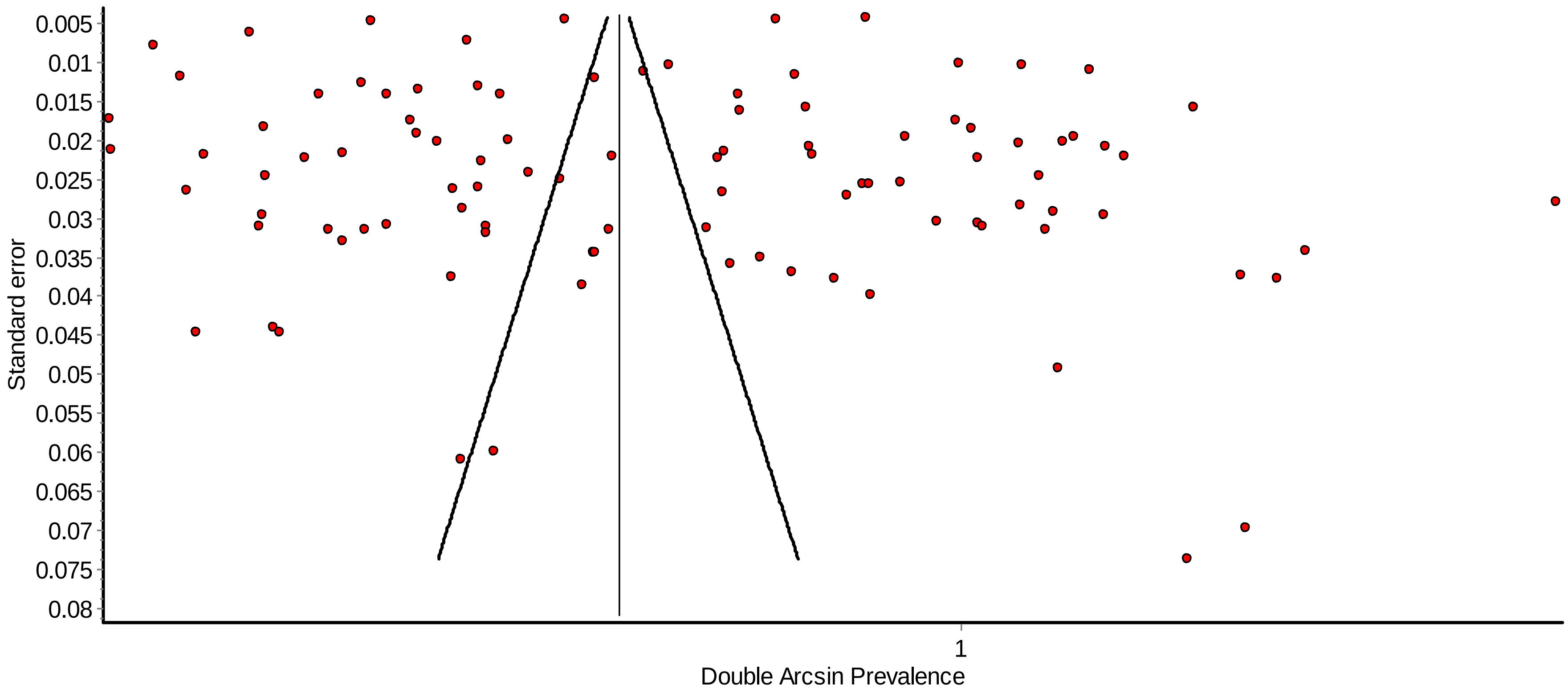

Figure S1. Funnel plot showing all studies included in the analysis for global prevalence of GORD.

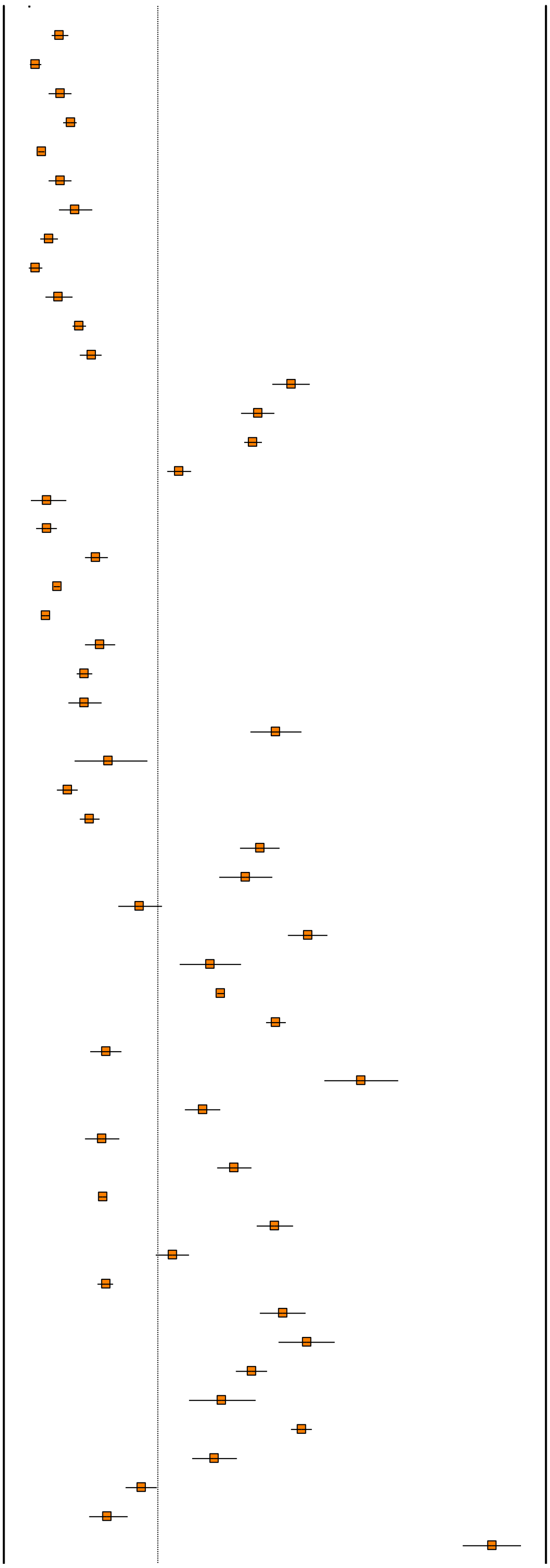

Study	Prev (95% CI)	% Weight
Cai et al., 2015	0.05 (0.04, 0.06)	1.0
Chen et al., 2005	0.02 (0.02, 0.03)	1.0
Cheung et al., 2007	0.05 (0.04, 0.06)	1.0
Guozong et al., 2000	0.06 (0.05, 0.06)	1.0
He et al., 2010	0.03 (0.03, 0.03)	1.0
Hu et al., 2002	0.05 (0.04, 0.06)	1.0
Ma et al., 2009	0.06 (0.05, 0.08)	1.0
Tan et al., 2016	0.04 (0.03, 0.05)	1.0
Wong et al., 2003	0.02 (0.02, 0.03)	1.0
Zou et al., 2011	0.05 (0.03, 0.06)	1.0
Fujiwara et al., 2005	0.07 (0.06, 0.07)	1.0
Kaji et al., 2010	0.08 (0.07, 0.09)	1.0
Kusano et al., 2008	0.27 (0.25, 0.28)	1.0
Murao et al., 2011	0.23 (0.22, 0.25)	1.0
Murase et al., 2014	0.23 (0.22, 0.24)	1.0
Niigaki et al., 2013	0.16 (0.15, 0.17)	1.0
Stanghellini, 1999a	0.04 (0.02, 0.05)	1.0
Jeong et al., 2008	0.04 (0.03, 0.05)	1.0
Kang et al., 2007	0.08 (0.07, 0.09)	1.0
Kim et al., 2007	0.05 (0.04, 0.05)	1.0
Lee et al., 2001	0.03 (0.03, 0.04)	1.0
Lee et al., 2009	0.09 (0.07, 0.10)	1.0
Min et al., 2014	0.07 (0.06, 0.08)	1.0
Yang et al., 2008	0.07 (0.06, 0.09)	1.0
Hung et al., 2011	0.25 (0.23, 0.27)	1.0
Abdullah et al., 2016	0.09 (0.06, 0.13)	0.9
Shaha et al., 2012	0.06 (0.05, 0.07)	1.0
Bhatia et al., 2011	0.08 (0.07, 0.09)	1.0
Kumar and Shivalli, 2014	0.24 (0.22, 0.25)	1.0
Wang et al., 2016	0.22 (0.20, 0.25)	1.0
Aletaha et al., 2007	0.12 (0.10, 0.14)	1.0
Darvishmoghadam et al., 2016	0.28 (0.26, 0.30)	1.0
Ehsani et al., 2007	0.19 (0.16, 0.22)	1.0
Islami et al., 2014	0.20 (0.20, 0.20)	1.0
Khodamoradi et al., 2017	0.25 (0.24, 0.26)	1.0
Mansour-Ghanaei et al., 2013	0.09 (0.08, 0.11)	1.0
Mostaghni et al., 2009	0.33 (0.30, 0.37)	1.0
Nasseri-Moghaddam et al., 2008	0.18 (0.17, 0.20)	1.0
Nouraie et al., 2007a	0.09 (0.07, 0.10)	1.0
Nouraie et al., 2007b	0.21 (0.20, 0.23)	1.0
Pourhoseingholi et al., 2012	0.09 (0.08, 0.09)	1.0
Rogha et al., 2006	0.25 (0.23, 0.27)	1.0
Saberi-Firoozi et al., 2007	0.15 (0.14, 0.17)	1.0
Solhpour et al., 2008	0.09 (0.08, 0.10)	1.0
Vossoughinia et al., 2014	0.26 (0.24, 0.28)	1.0
Yadegarfar et al., 2018	0.28 (0.25, 0.31)	1.0
Bor et al., 2017	0.23 (0.21, 0.24)	1.0
Kitapcioglu et al., 2007	0.20 (0.17, 0.23)	1.0
Mungan, 2012	0.27 (0.27, 0.28)	1.0
Yonem et al., 2013	0.19 (0.17, 0.21)	1.0
Moshkowitz et al., 2011	0.12 (0.11, 0.14)	1.0
Sperber et al., 2007	0.09 (0.08, 0.11)	1.0
Almadi et al., 2014	0.45 (0.43, 0.48)	1.0

Figure S2. Continued.

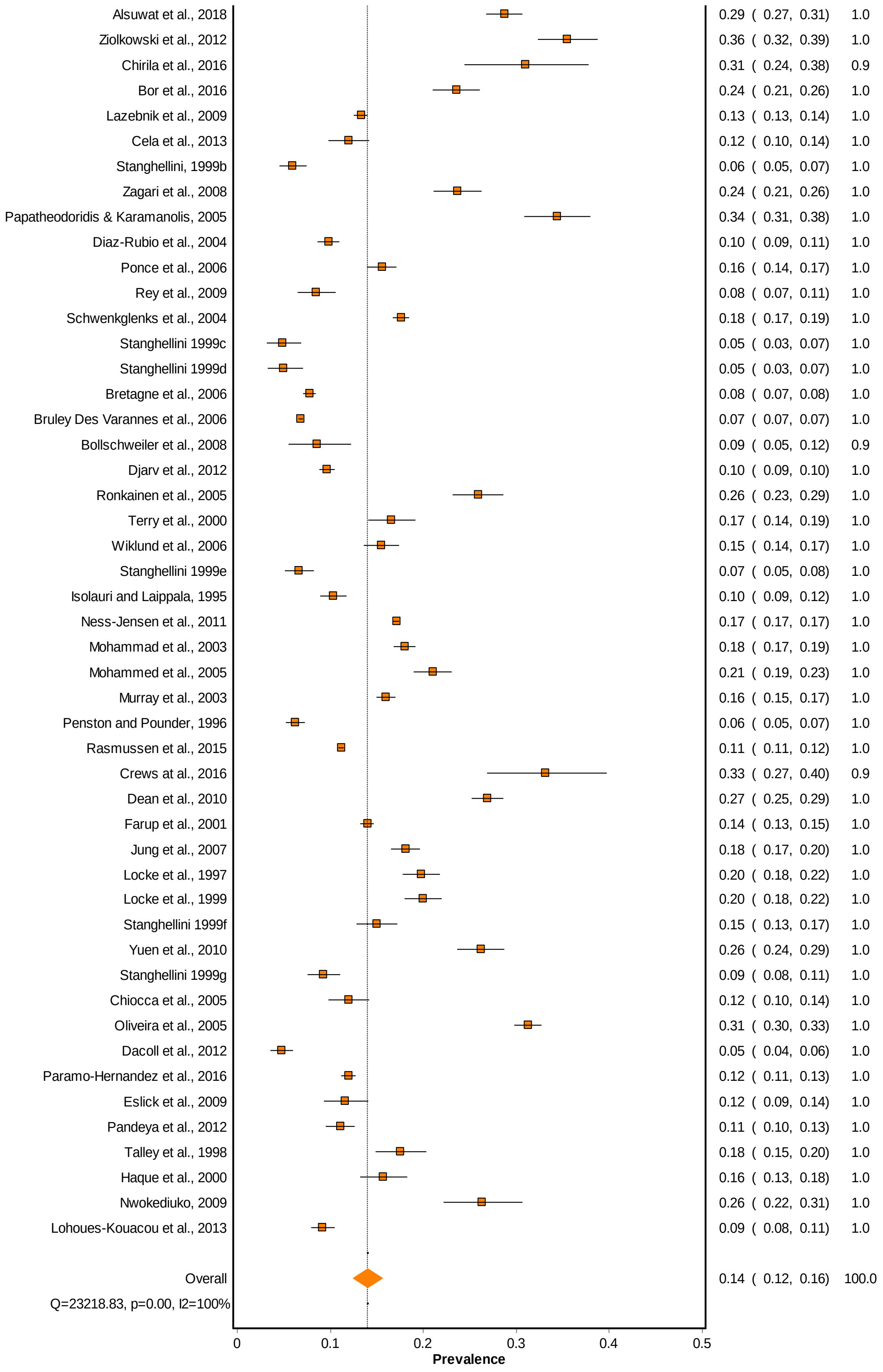

Figure S2. Forest plot showing global prevalence of GORD with studies pooled using a random effects model.

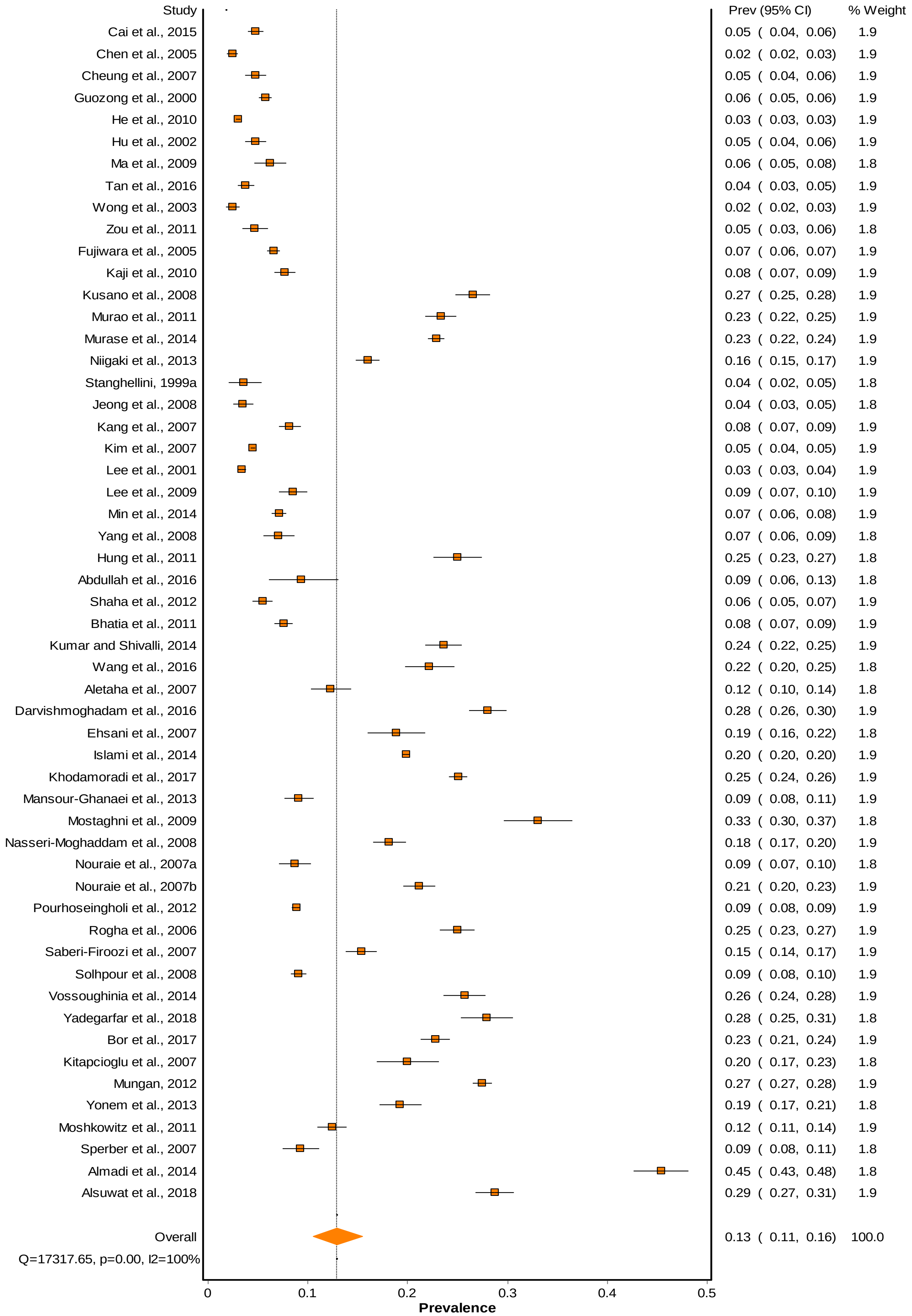

Figure S3. Forest plot showing prevalence of GORD in Asia with studies pooled using a random effects model.

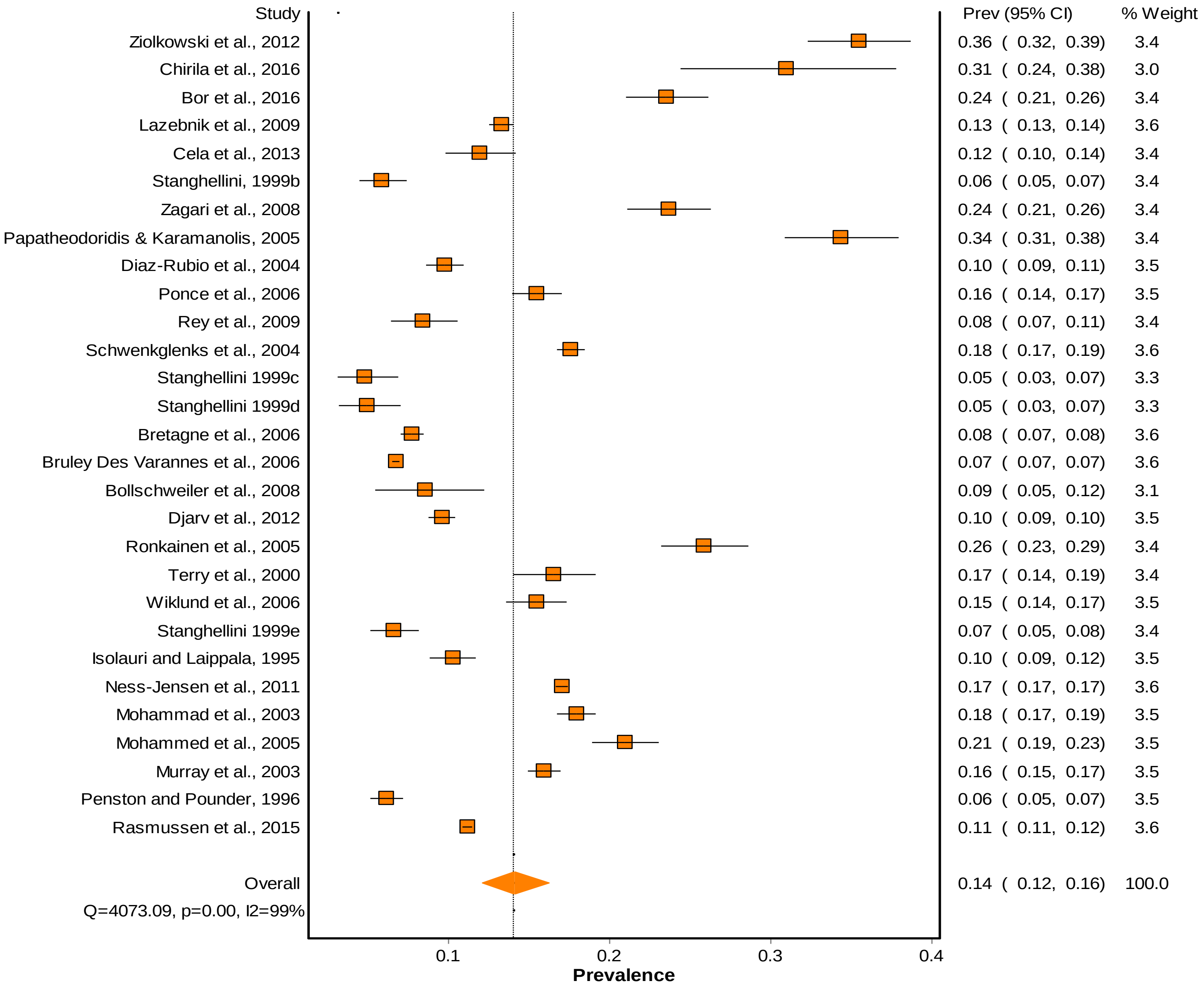

Figure S4. Forest plot showing prevalence of GORD in Europe with studies pooled using a random effects model.

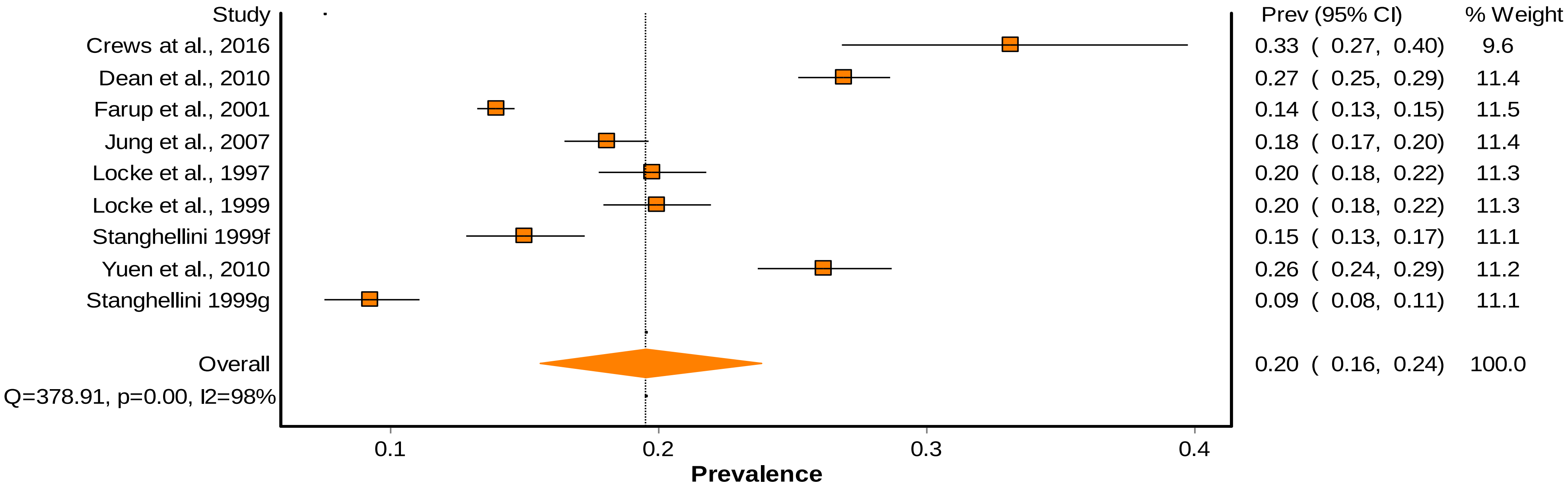

Figure S5. Forest plot showing prevalence of GORD in North America with studies pooled using a random effects model.

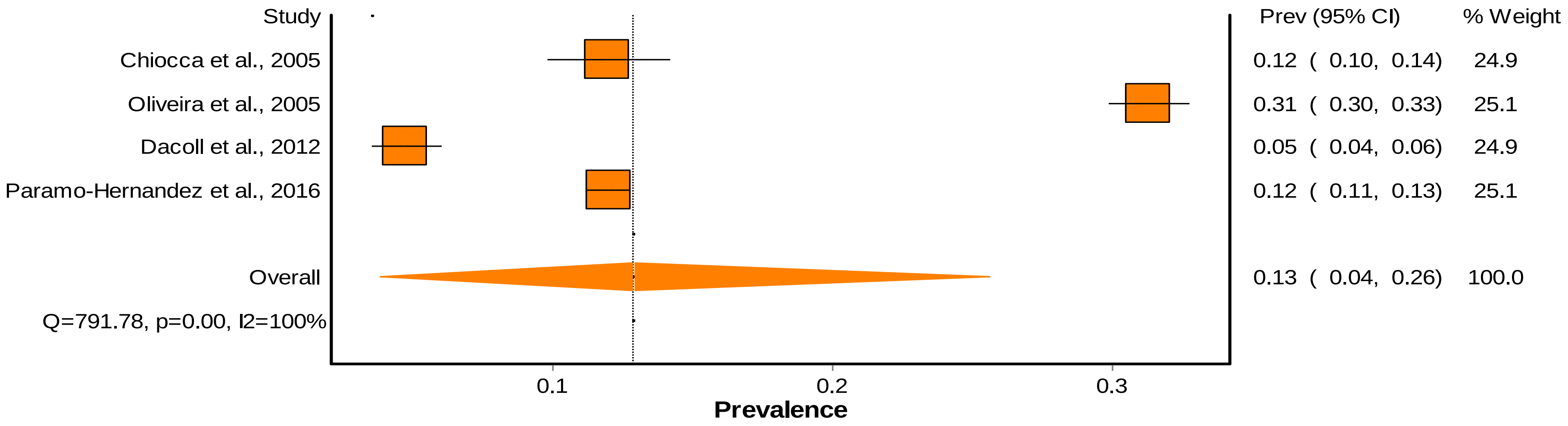

Figure S6. Forest plot showing prevalence of GORD in Latin America and the Caribbean with studies pooled using a random effects model.

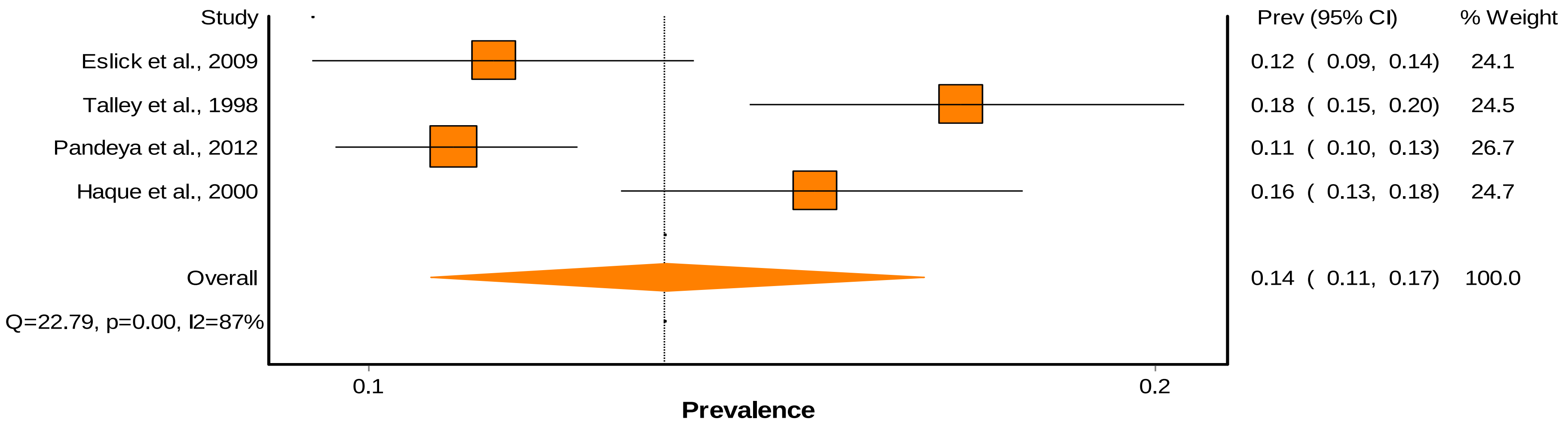

Figure S7. Forest plot showing prevalence of GORD in Oceania with studies pooled using a random effects model.

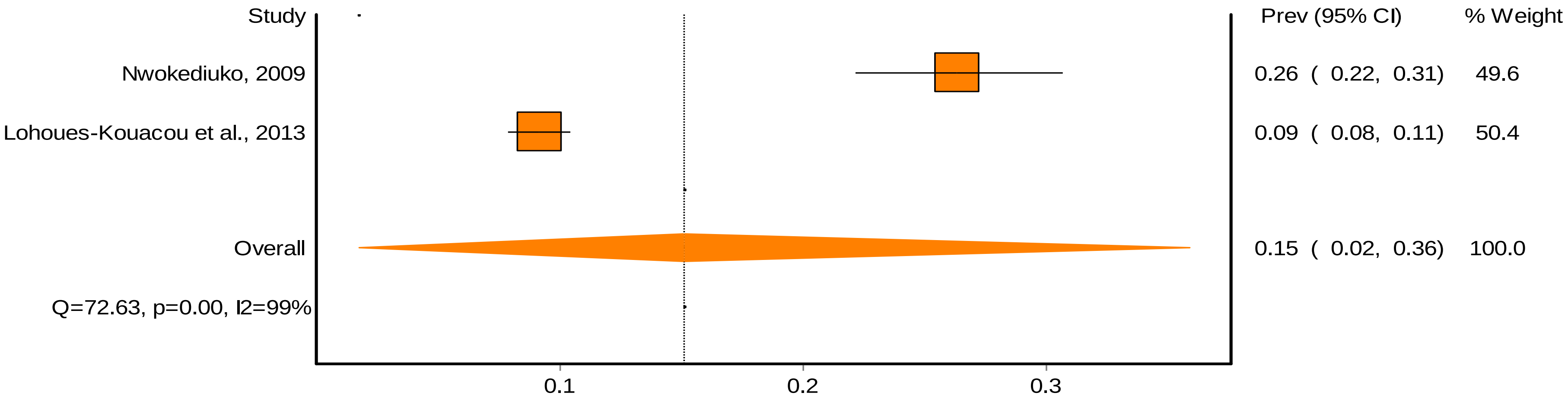

Figure S8. Forest plot showing prevalence of GORD in Africa with studies pooled using a random effects model.

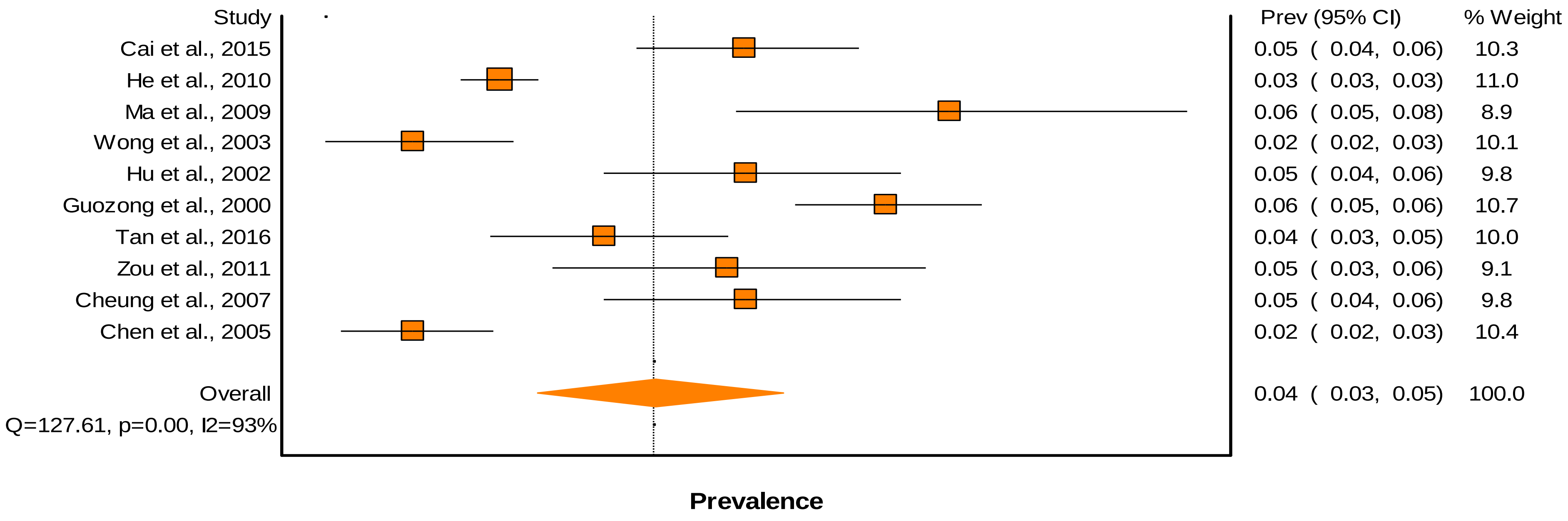

Figure S9. Forest plot showing the prevalence of GORD in China with studies pooled using a random effects model.

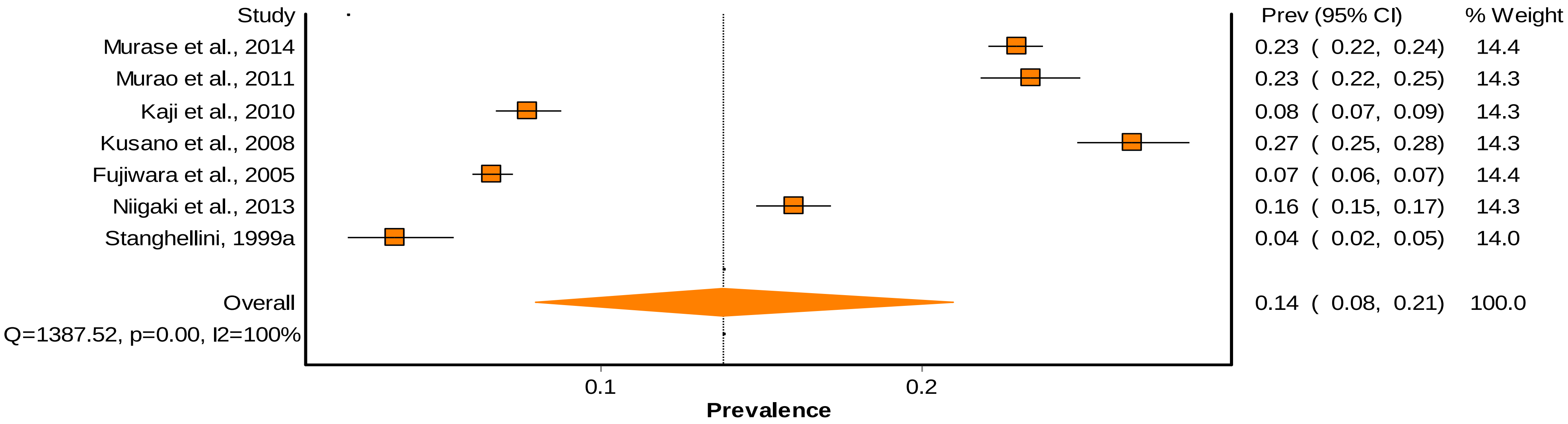

Figure S10. Forest plot showing the prevalence of GORD in Japan with studies pooled using a random effects model.

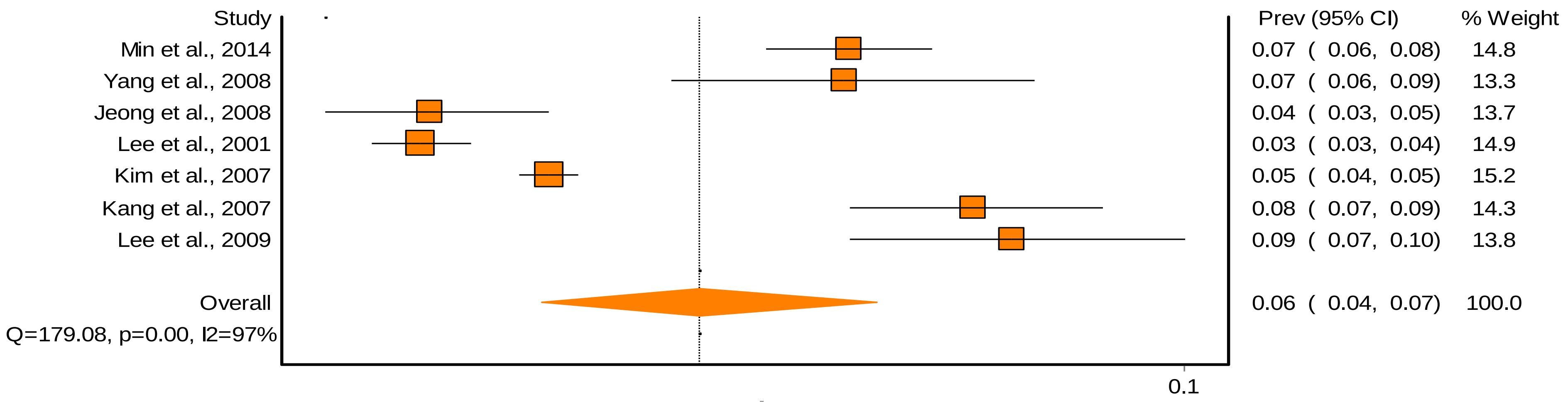

Figure S11. Forest plot showing the prevalence of GORD in South Korea with studies pooled using a random effects model.

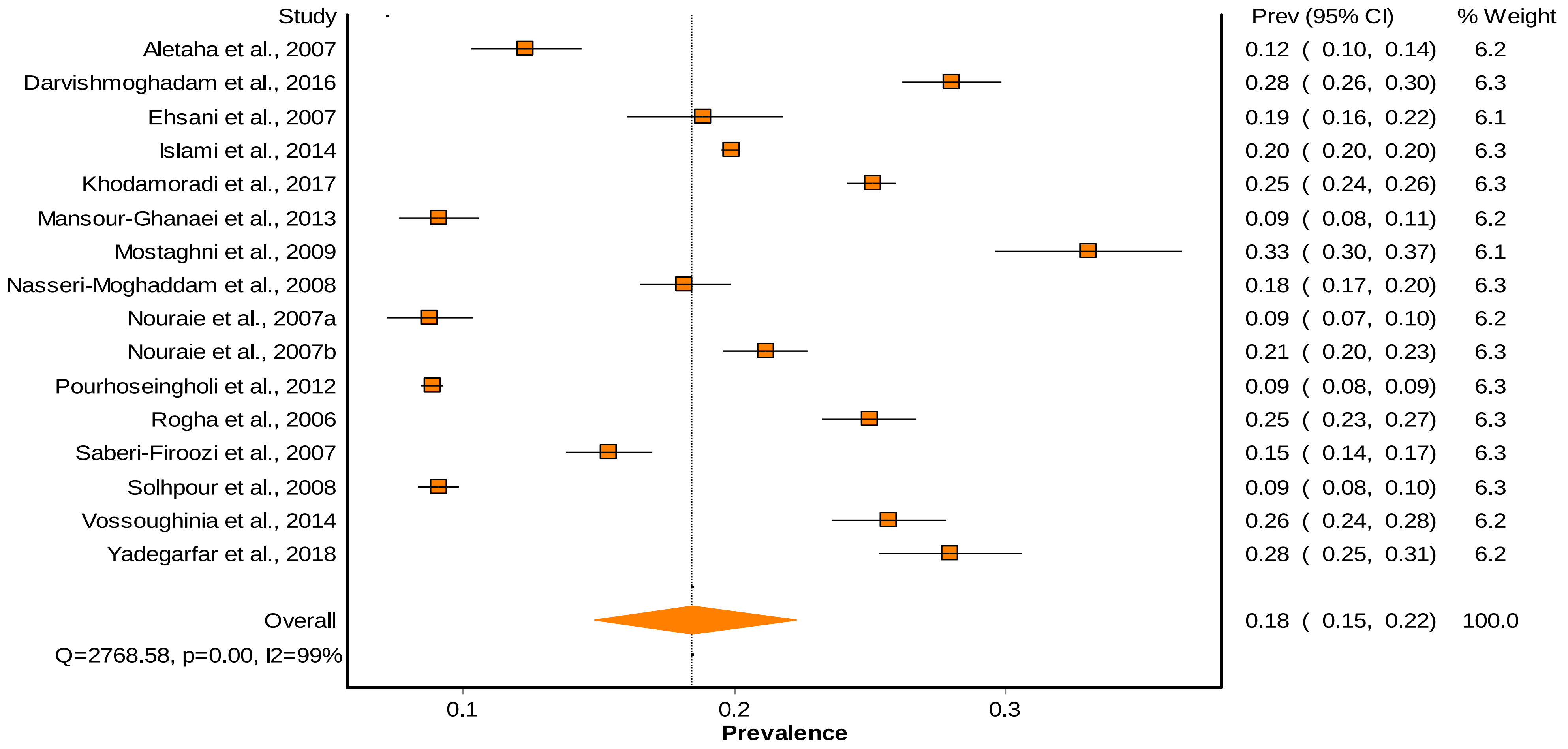

Figure S12. Forest plot showing the prevalence of GORD in Iran with studies pooled using a random effects model.

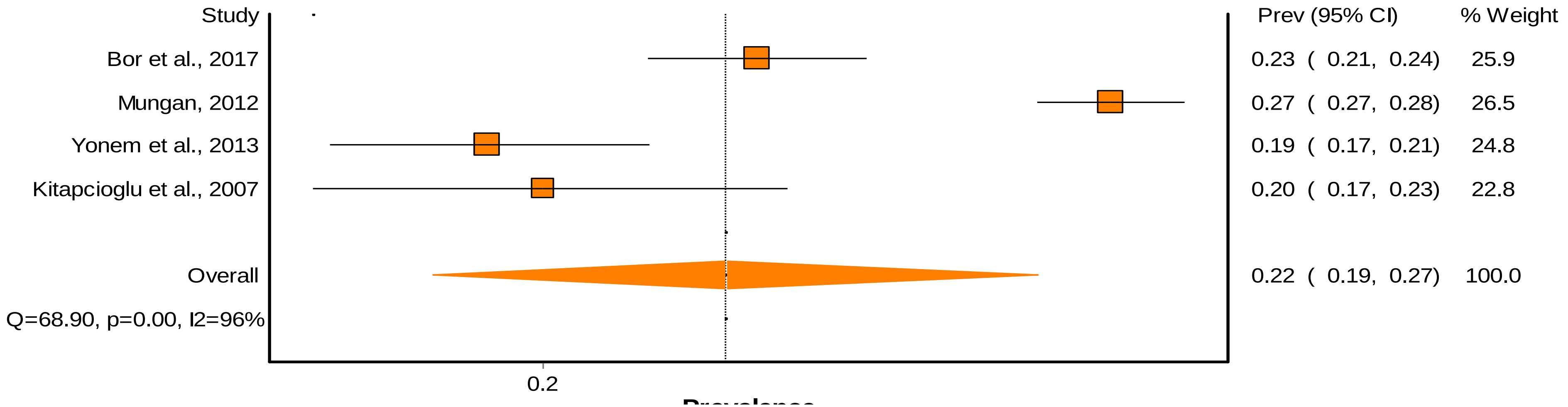

Figure S13. Forest plot showing the prevalence of GORD in Turkey with studies pooled using a random effects model.

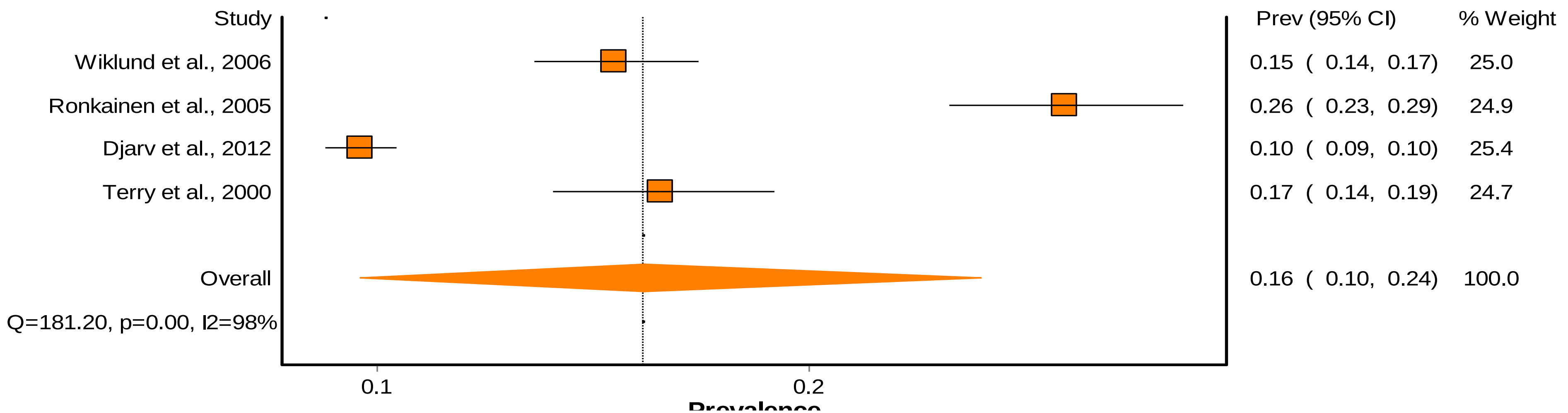

Figure S14. Forest plot showing the prevalence of GORD in Sweden with studies pooled using a random effects model.

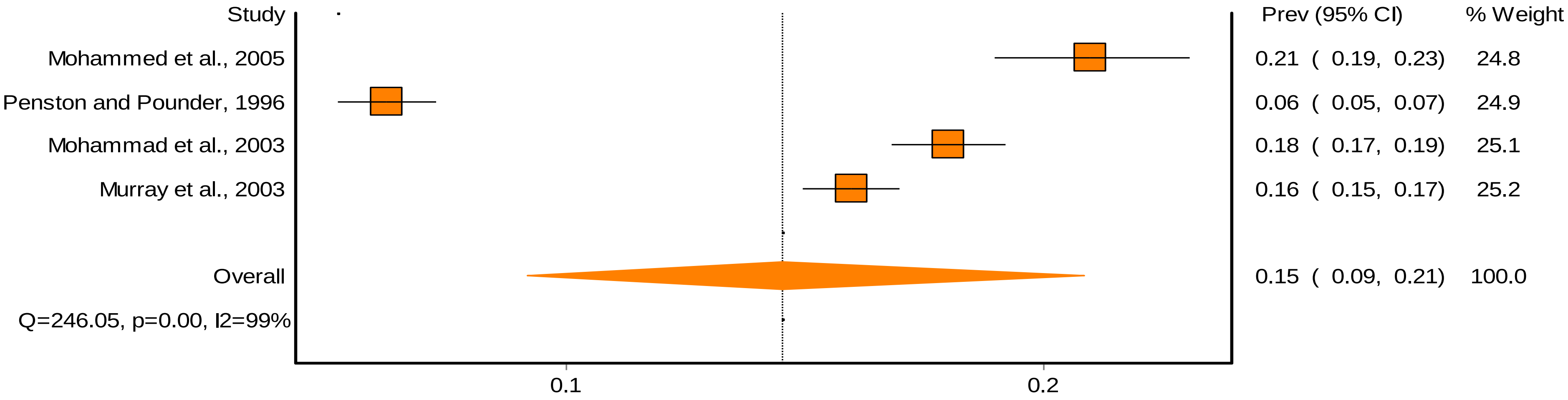

Figure S15. Forest plot showing the prevalence of GORD in UK with studies pooled using a random effects model.

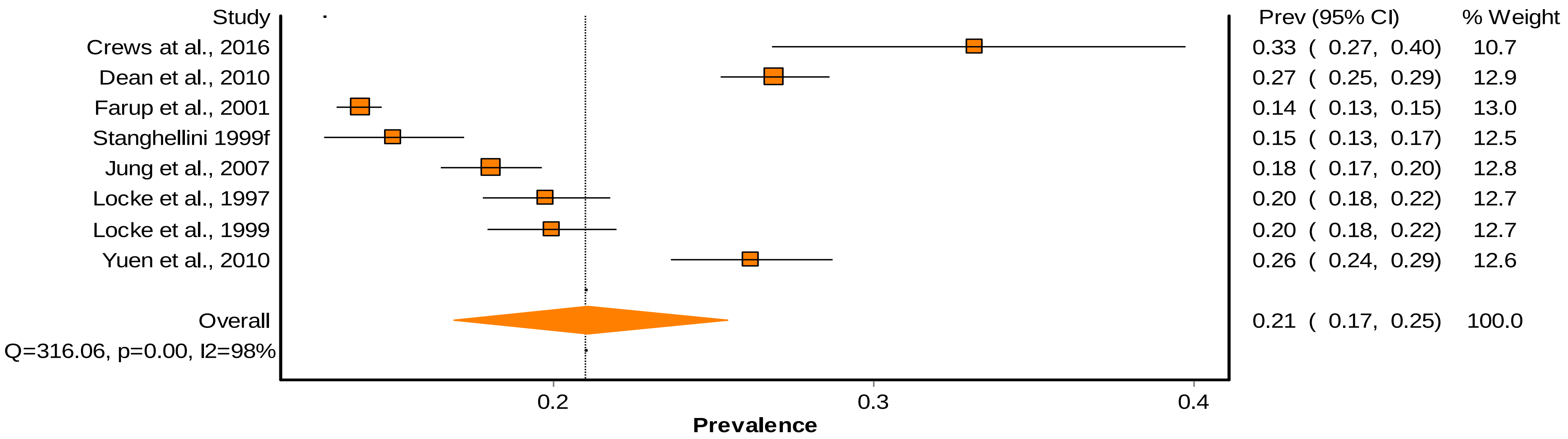

Figure S16. Forest plot showing the prevalence of GORD in USA with studies pooled using a random effects model.

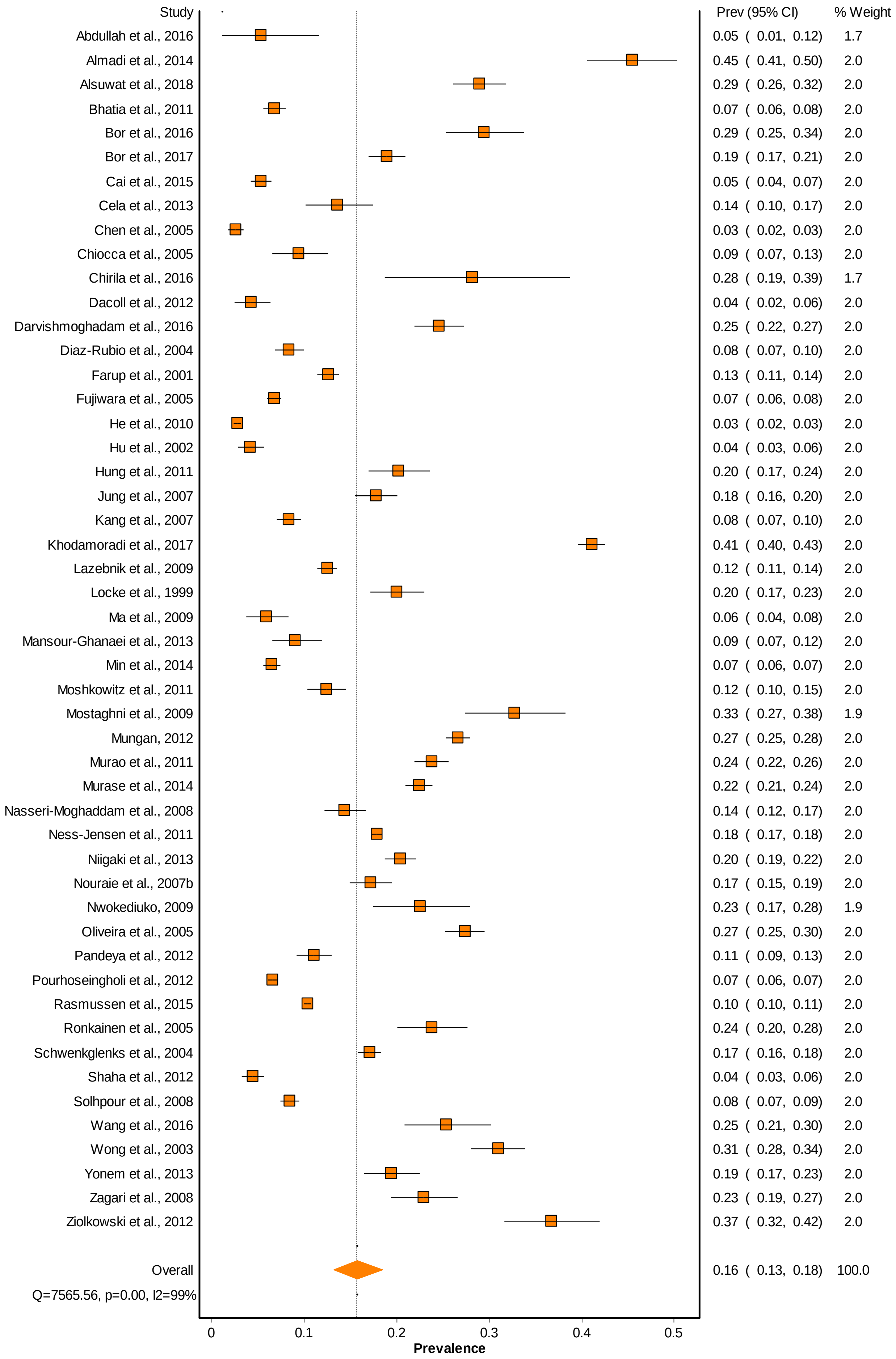

Figure S17. Forest plot showing global prevalence of GORD in males with studies pooled using a random effects model.

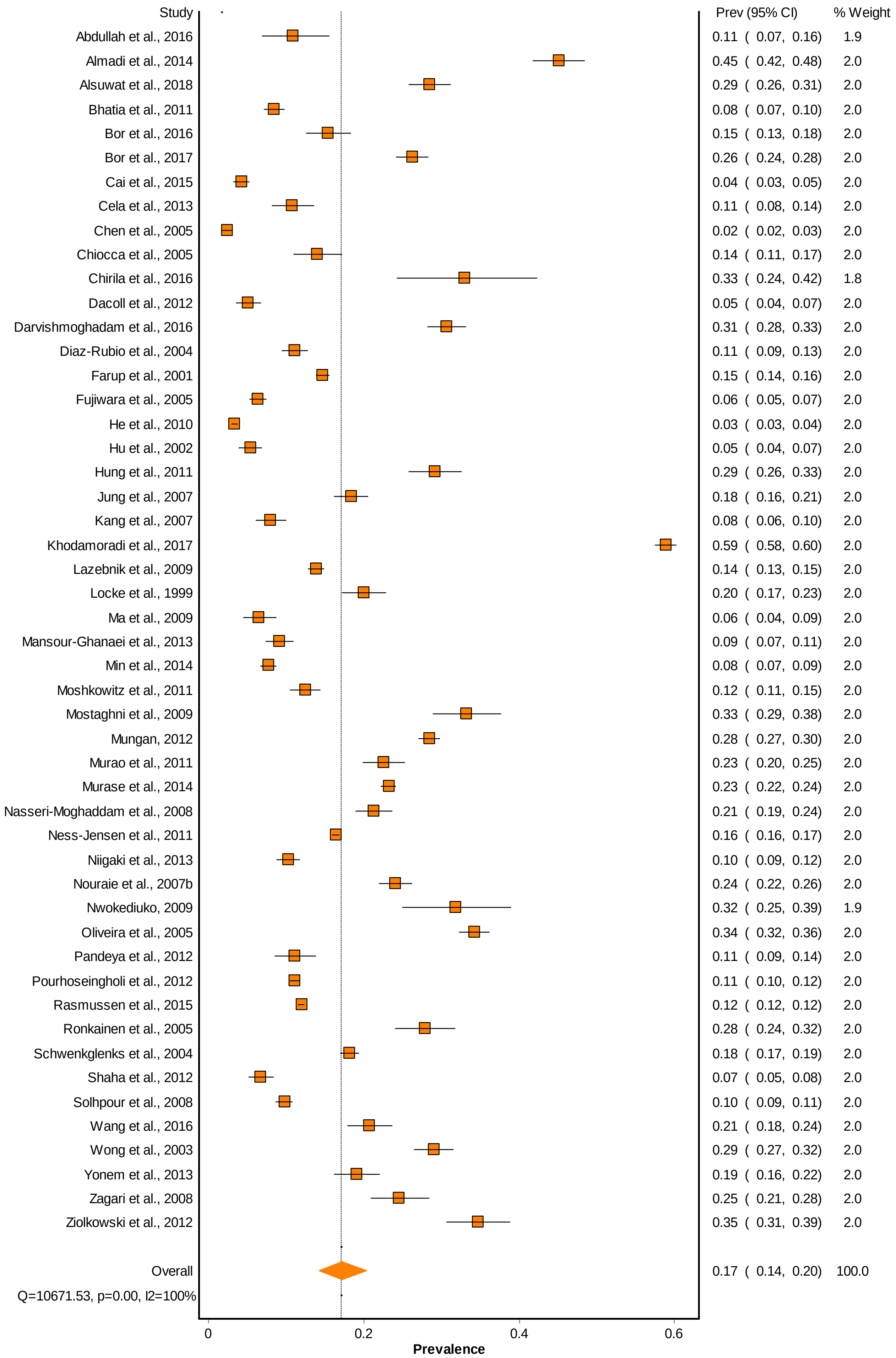

Figure S18. Forest plot showing global prevalence of GORD in females with studies pooled using a random effects model.

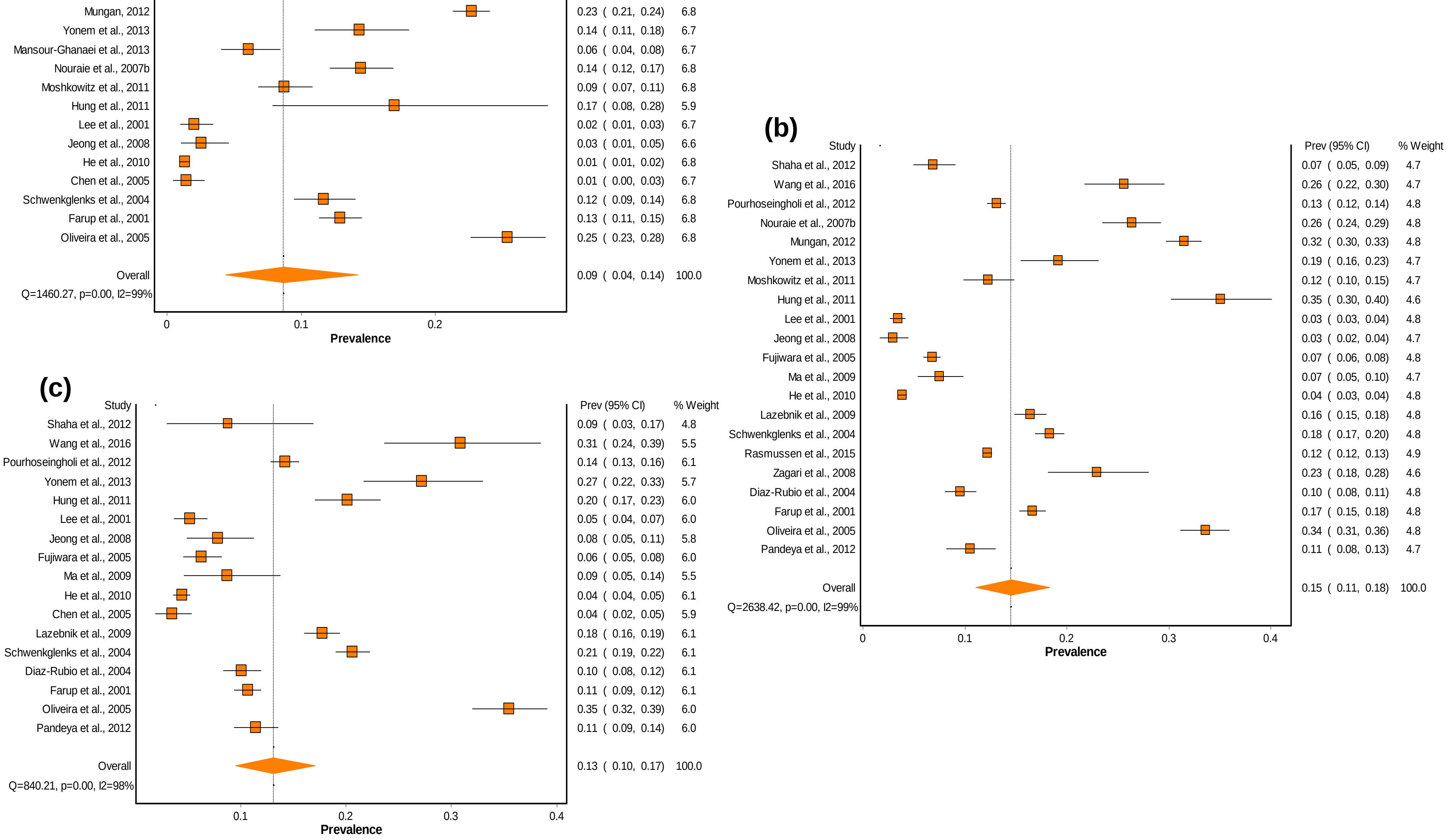

Figure S19. Forest plots showing global prevalence of GORD according to age groups with studies pooled using a random effects model.
(a) 18-34 years.
(b) 35-59 years.
(c) ≥60 years.

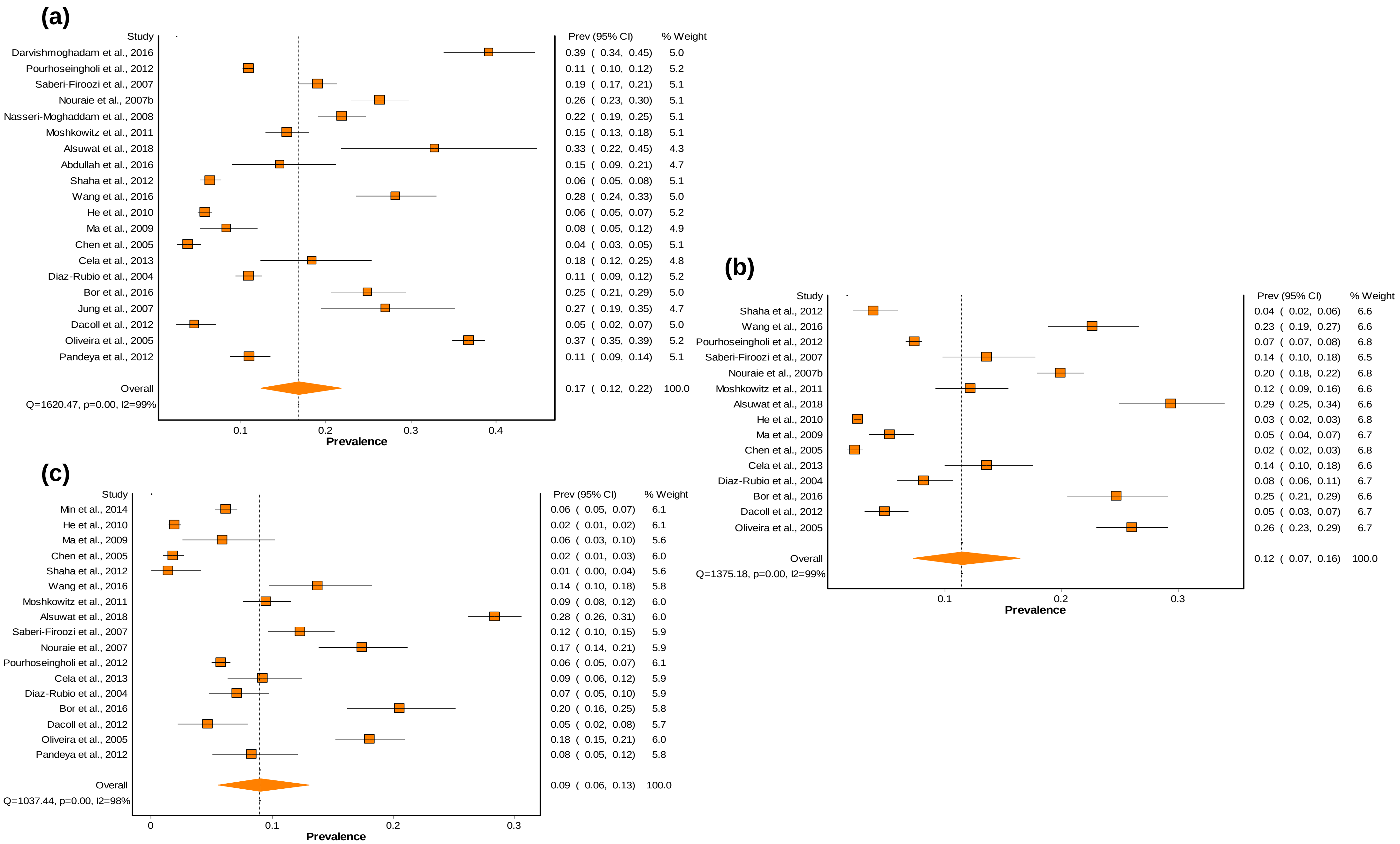

Figure S20. Forest plots showing global prevalence of GORD according to education level with studies pooled using a random effects model.
(a) Low education level.
(b) Medium education level.
(c) High education level.

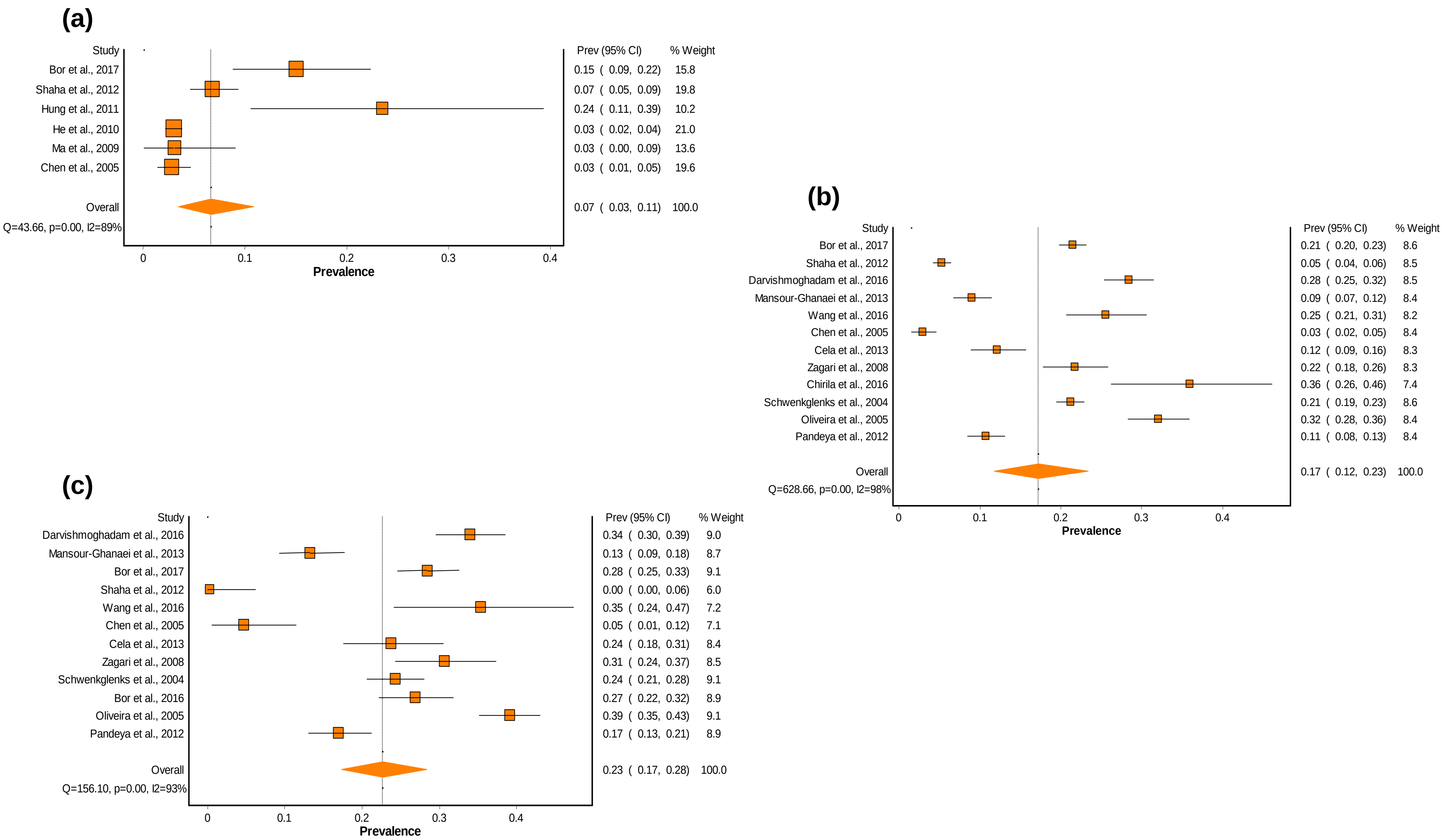

Figure S21. Forest plots showing global prevalence of GORD according to BMI level with studies pooled using a random effects model.
(a) <18.5
(b) 18.5-29.9
(c) ≥30

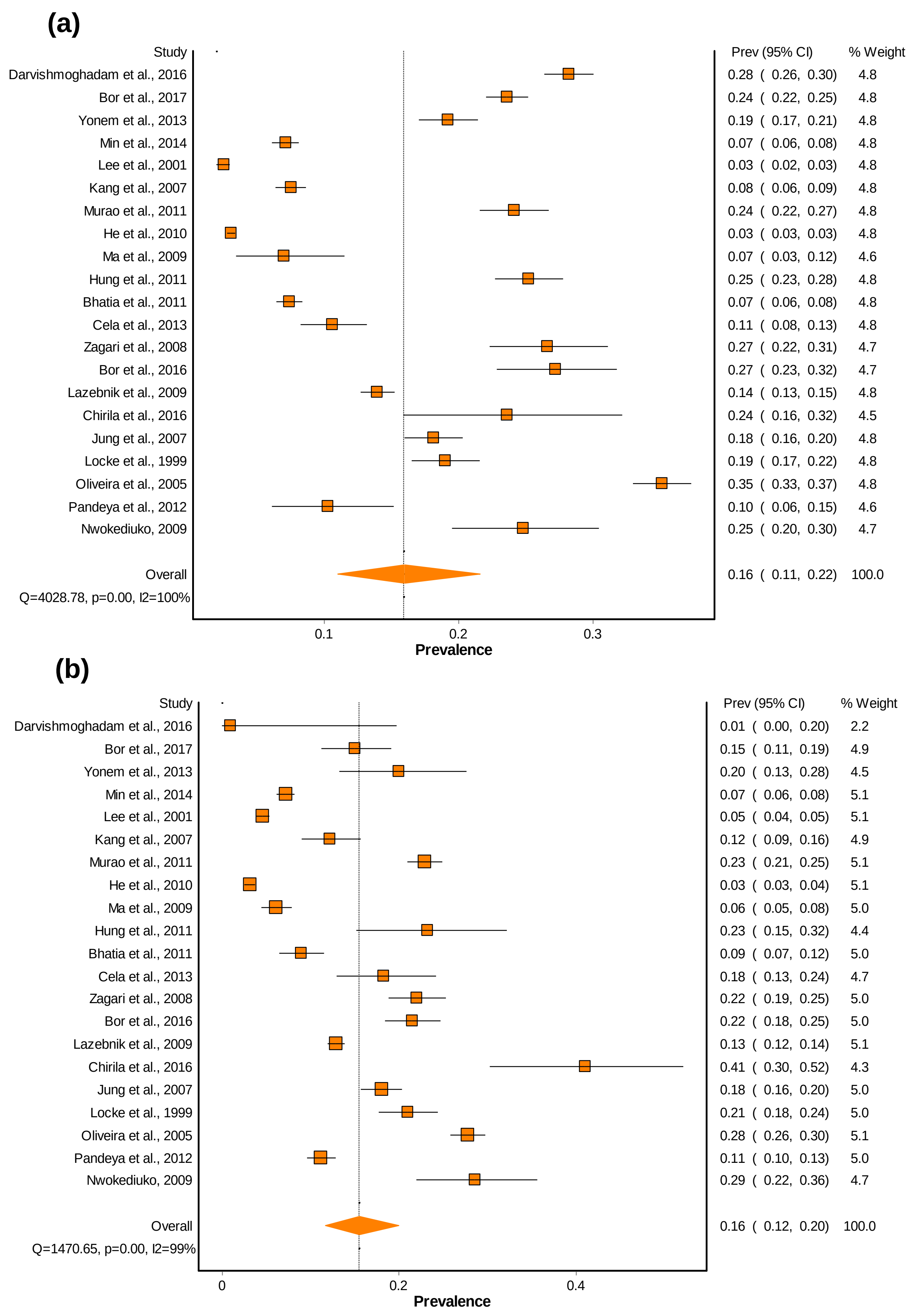

Figure S22. Forest plots showing global prevalence of GORD according to alcohol intake with studies pooled using a random effects model.
(a) None-low intake.
(b) Moderate-high intake.

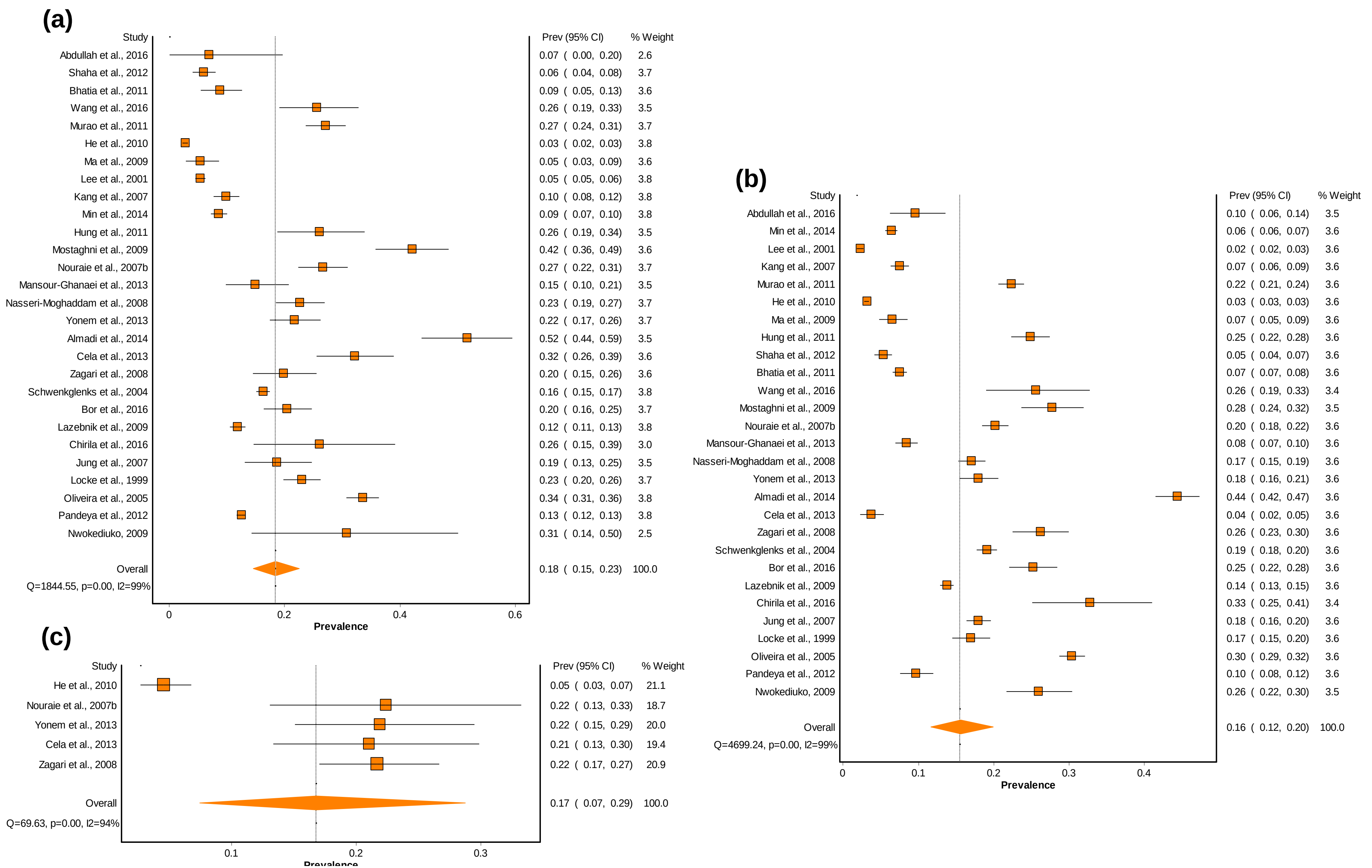

Figure S23. Forest plots showing global prevalence of GORD according to smoking habits with studies pooled using a random effects model.
(a) Current smokers.
(b) Non-smokers.
(c) Ex-smokers.

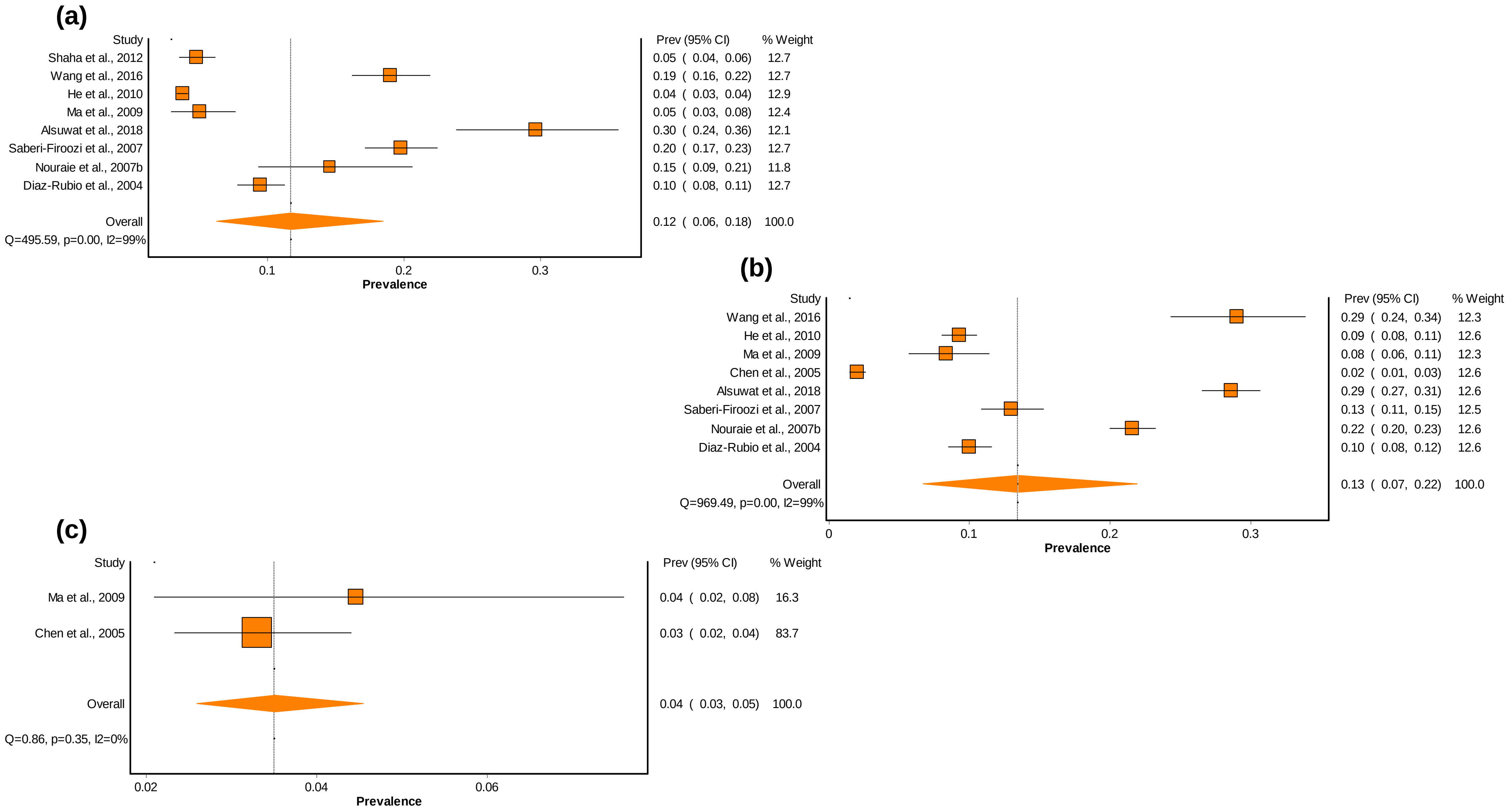

Figure S24. Forest plots showing global prevalence of GORD according to area of domicile with studies pooled using a random effects model.
(a) Rural.
(b) Urban.
(c) Suburban.

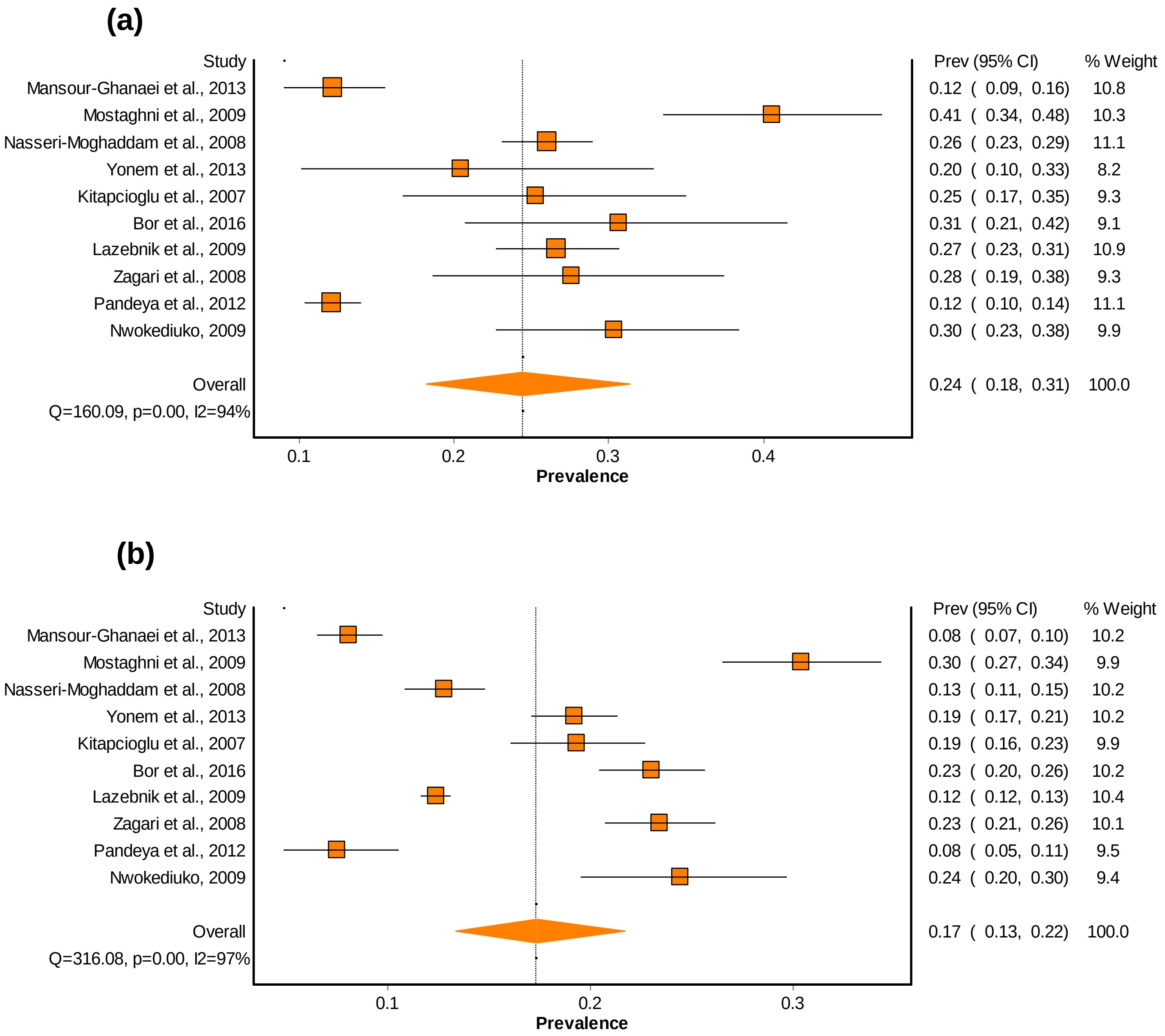

Figure S25. Forest plots showing global prevalence of GORD according to NSAIDs/aspirin use with studies pooled using a random effects model.
(a) NSAIDs/aspirin users.
(b) NSAIDs/aspirin non-users.

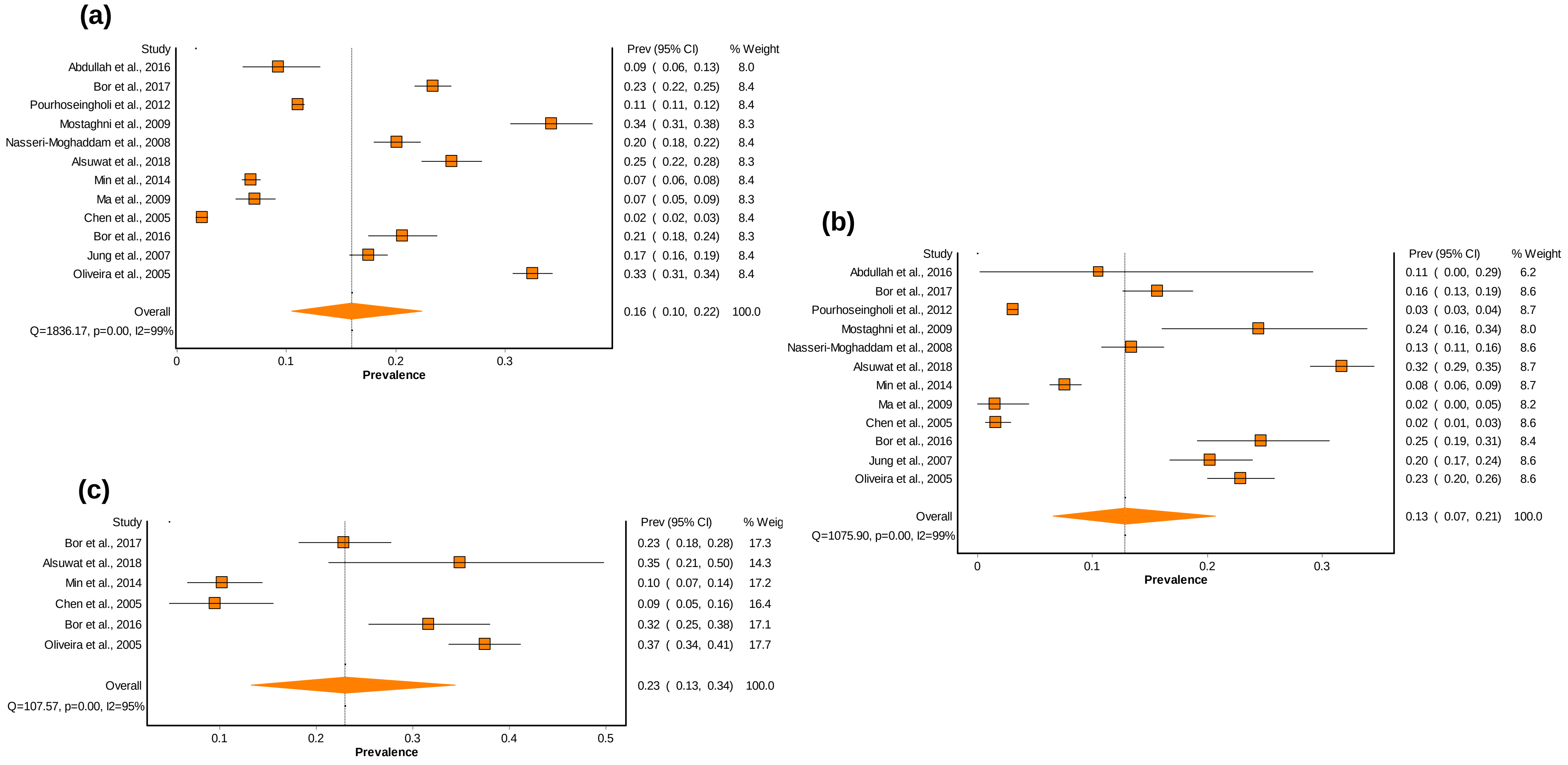

Figure S26. Forest plots showing global prevalence of GORD according to marriage status with studies pooled using a random effects model.
(a) Married.
(b) Single.
(c) Divorced.

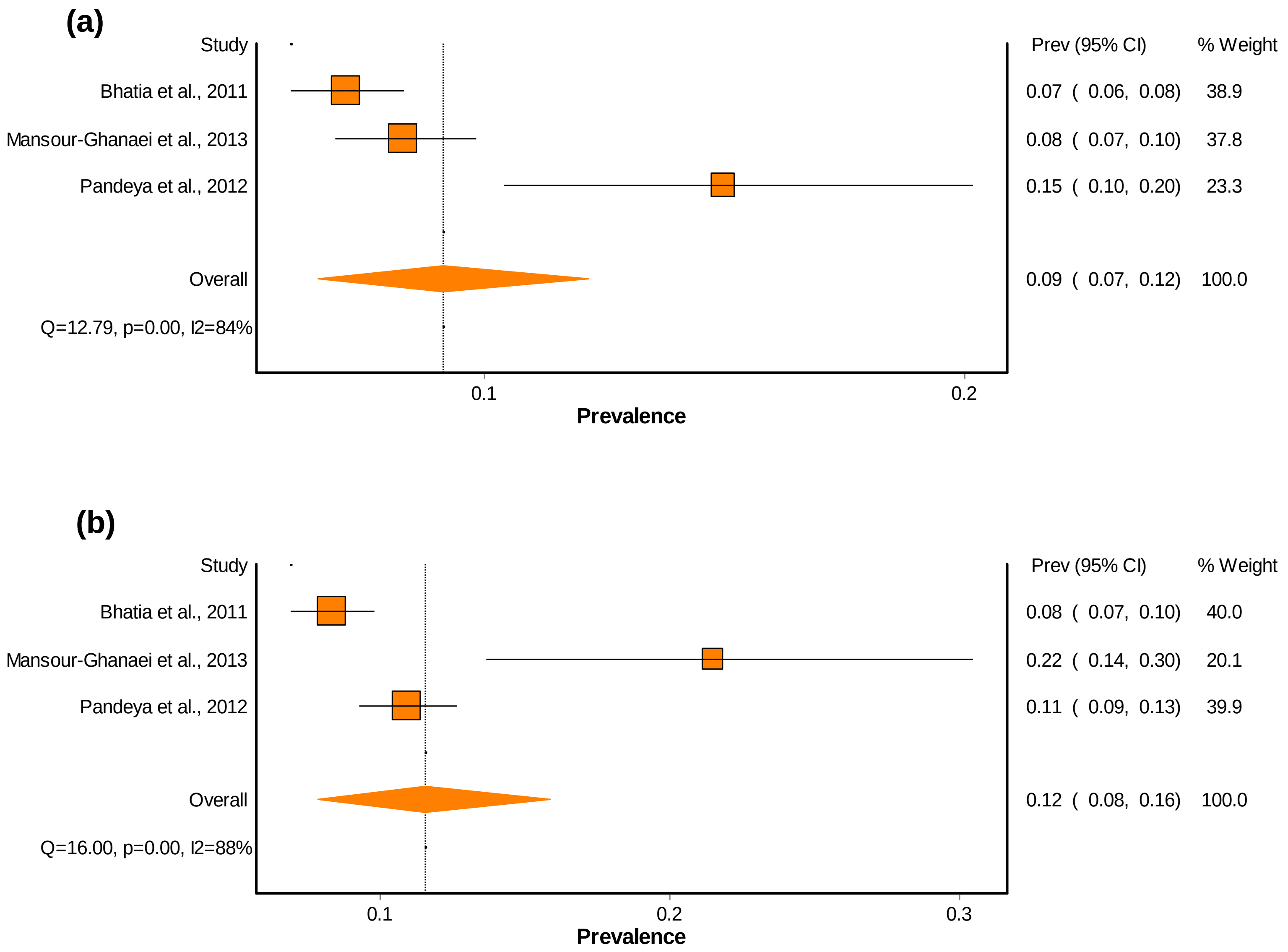

Figure S27. Forest plot showing global prevalence of GORD according to intake of spicy food with studies pooled using a random effects model.
(a) Low intake.
(b) High intake.

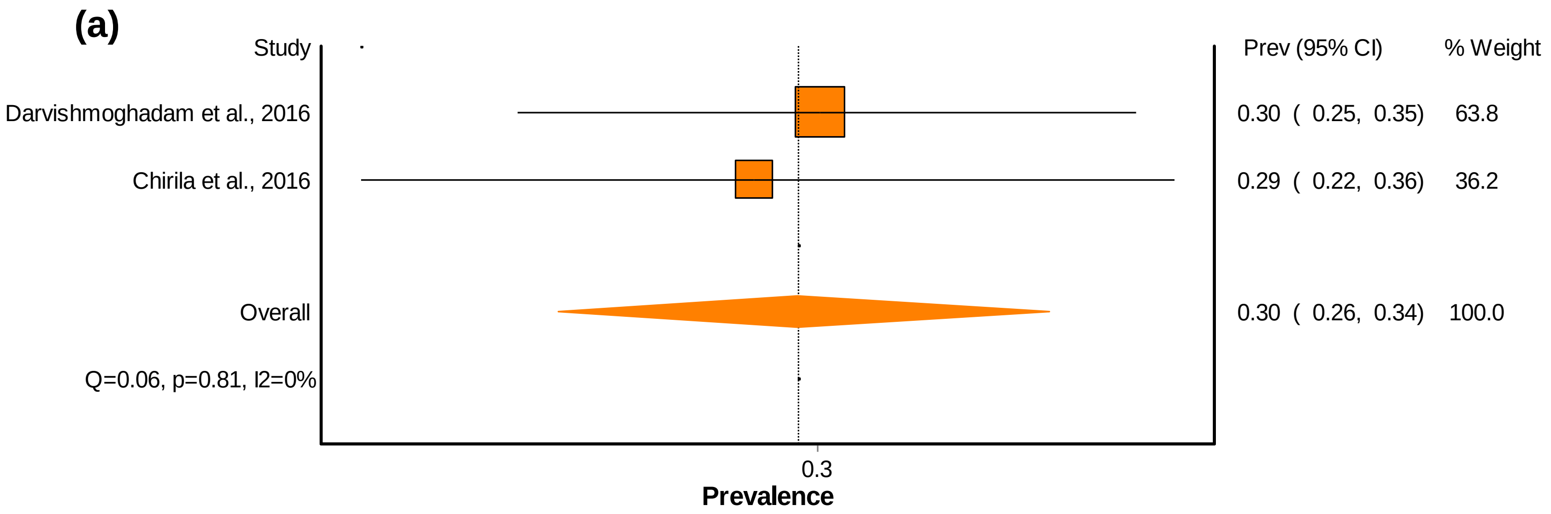

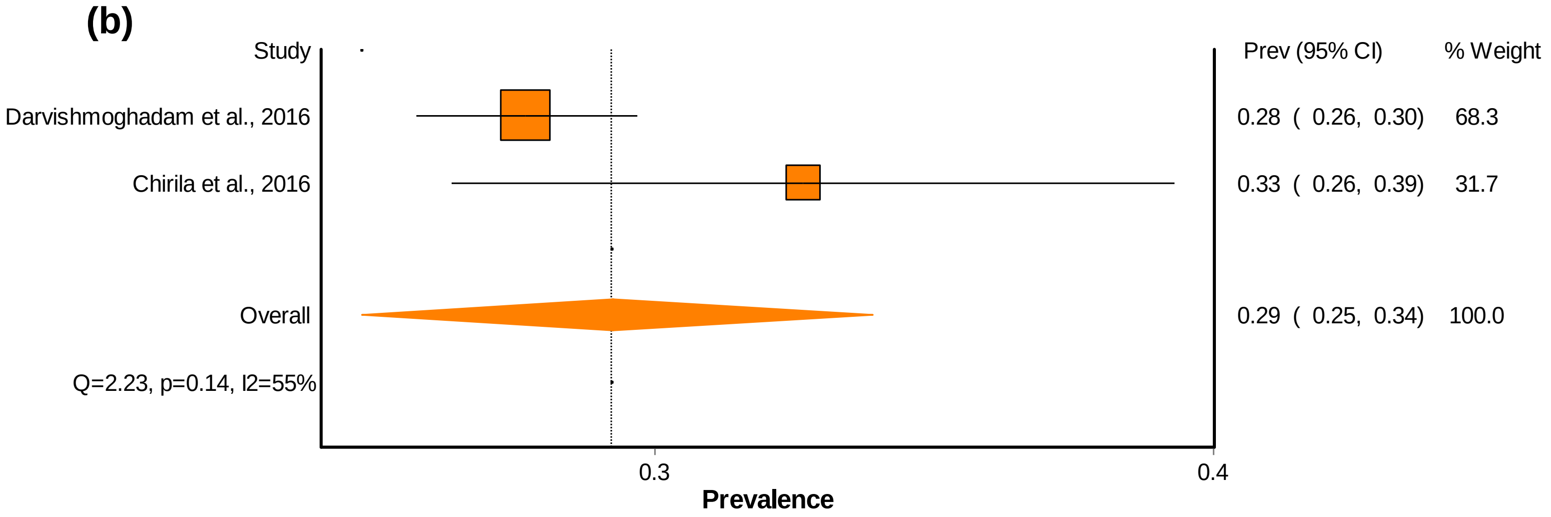

Figure S28. Forest plot showing global prevalence of GORD according to intake of sweet food with studies pooled using a random effects model.
(a) Low intake.
(b) High intake.

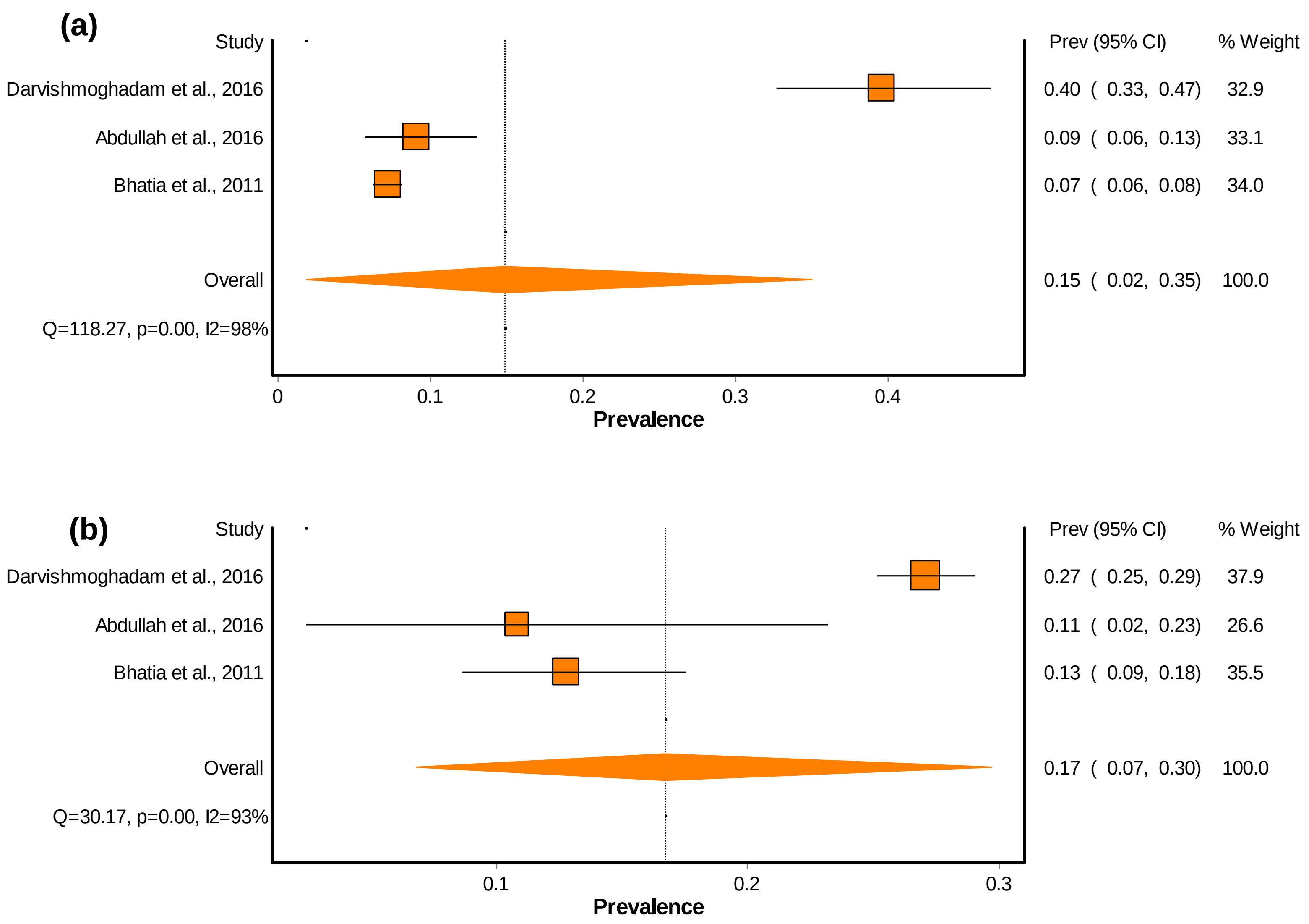

Figure S29. Forest plot showing global prevalence of GORD according to intake of meat/fish with studies pooled using a random effects model.
(a) Low intake.
(b) High intake.

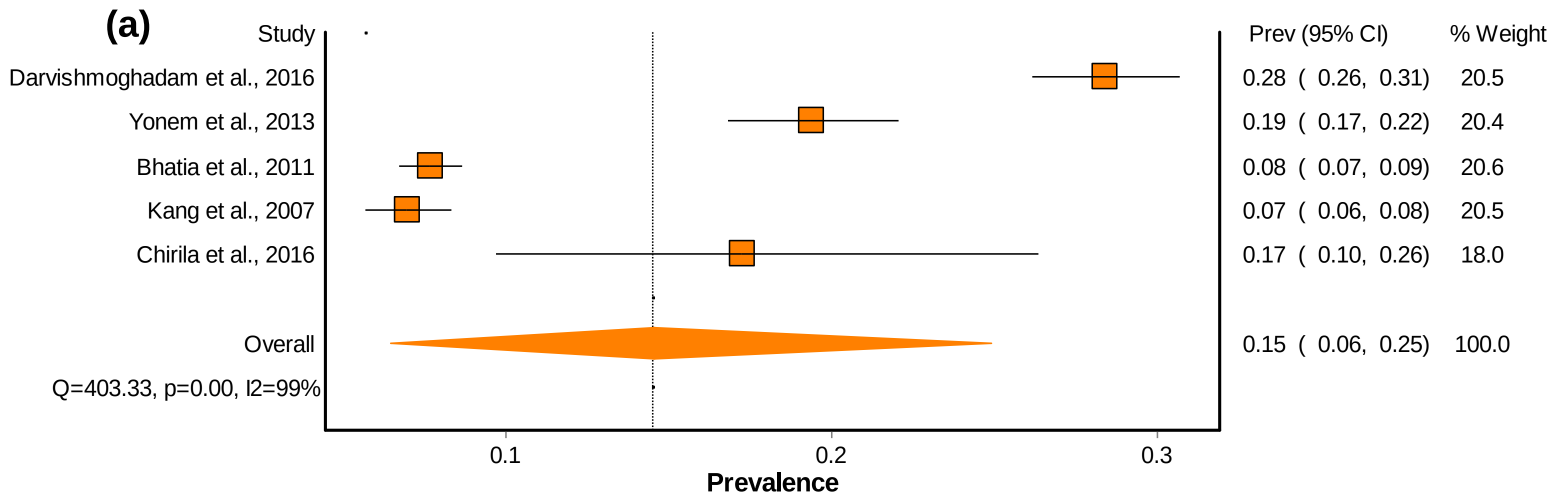

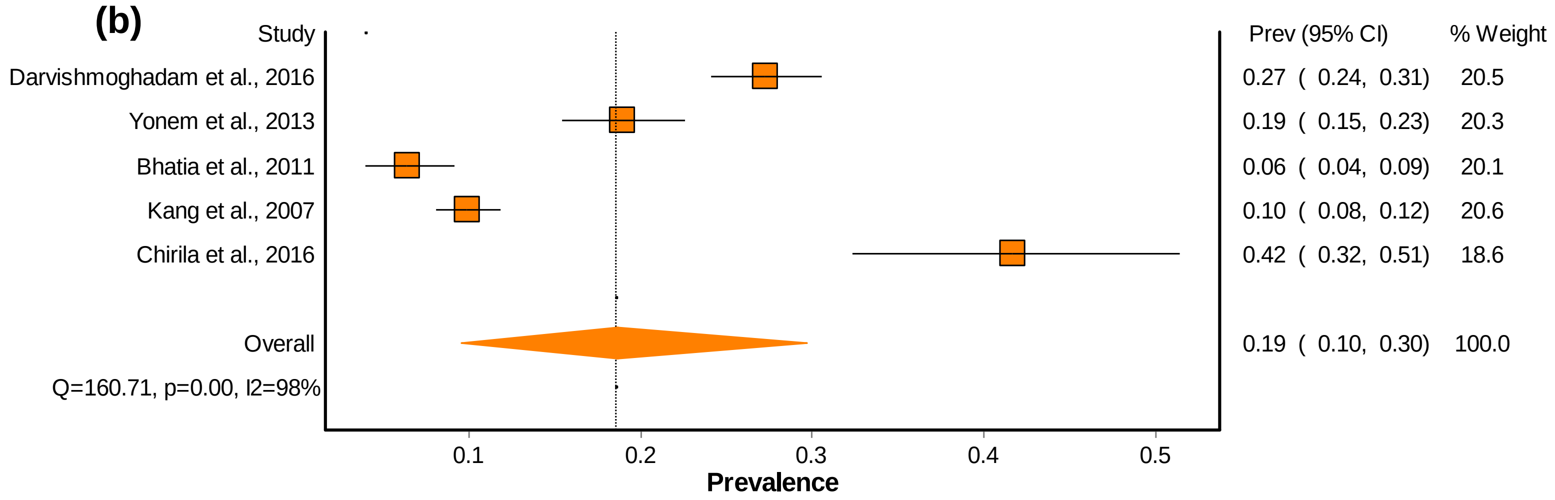

Figure S30. Forest plot showing global prevalence of GORD according to intake of carbonated drinks with studies pooled using a random effects model.
(a) Low intake.
(b) High intake.

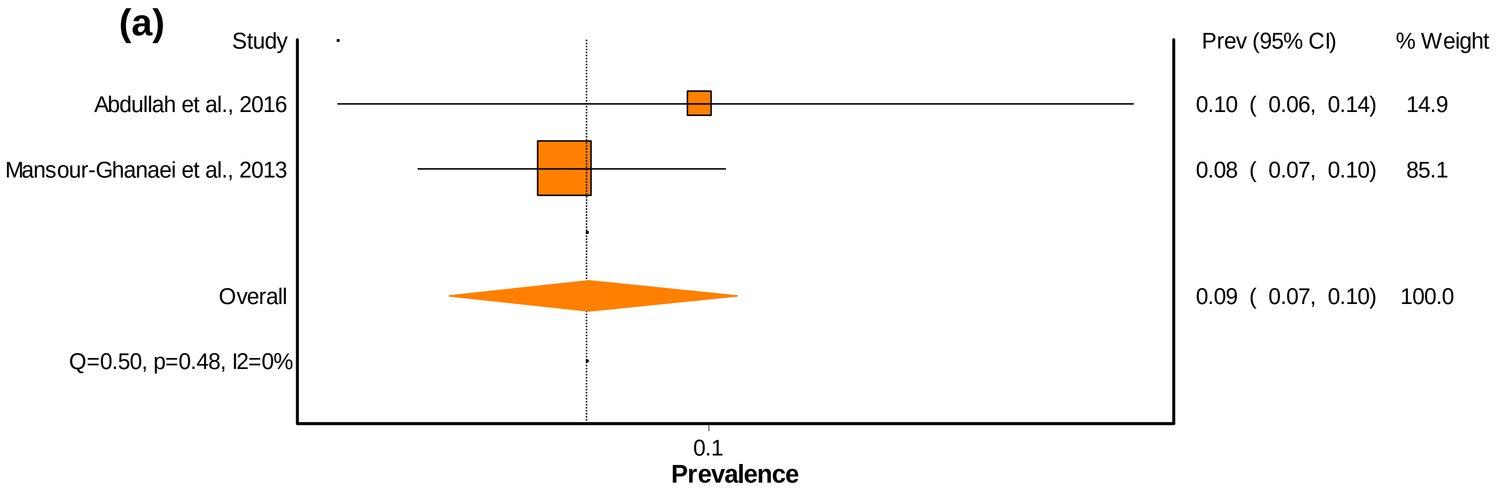

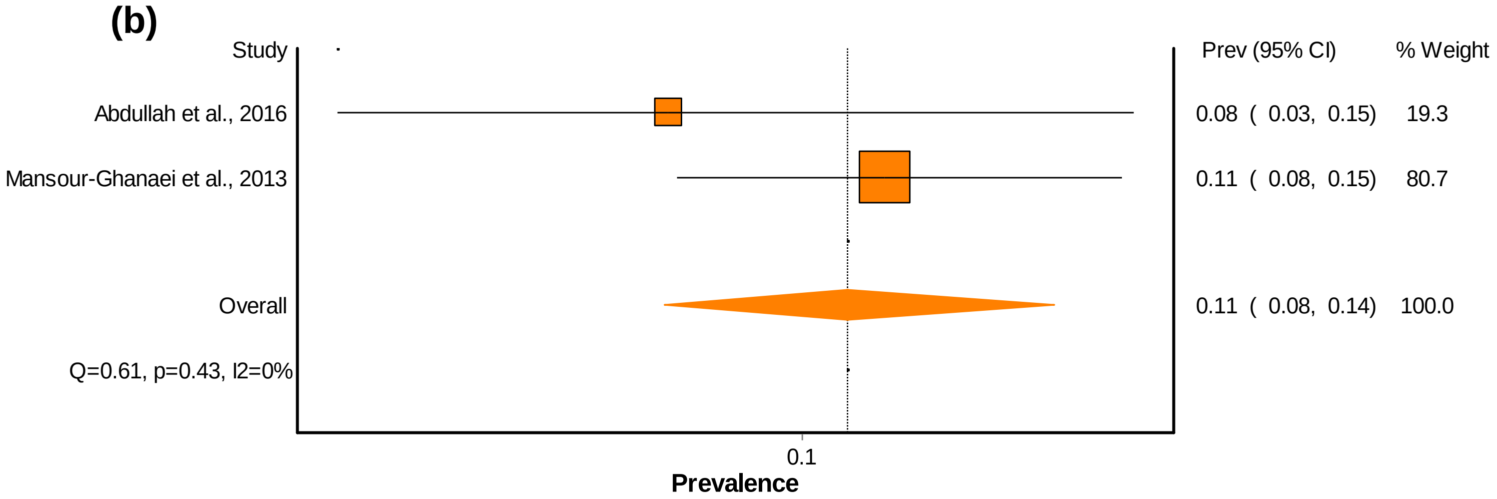

Figure S31. Forest plot showing global prevalence of GORD according to intake of fatty food with studies pooled using a random effects model.
(a) Low intake.
(b) High intake.

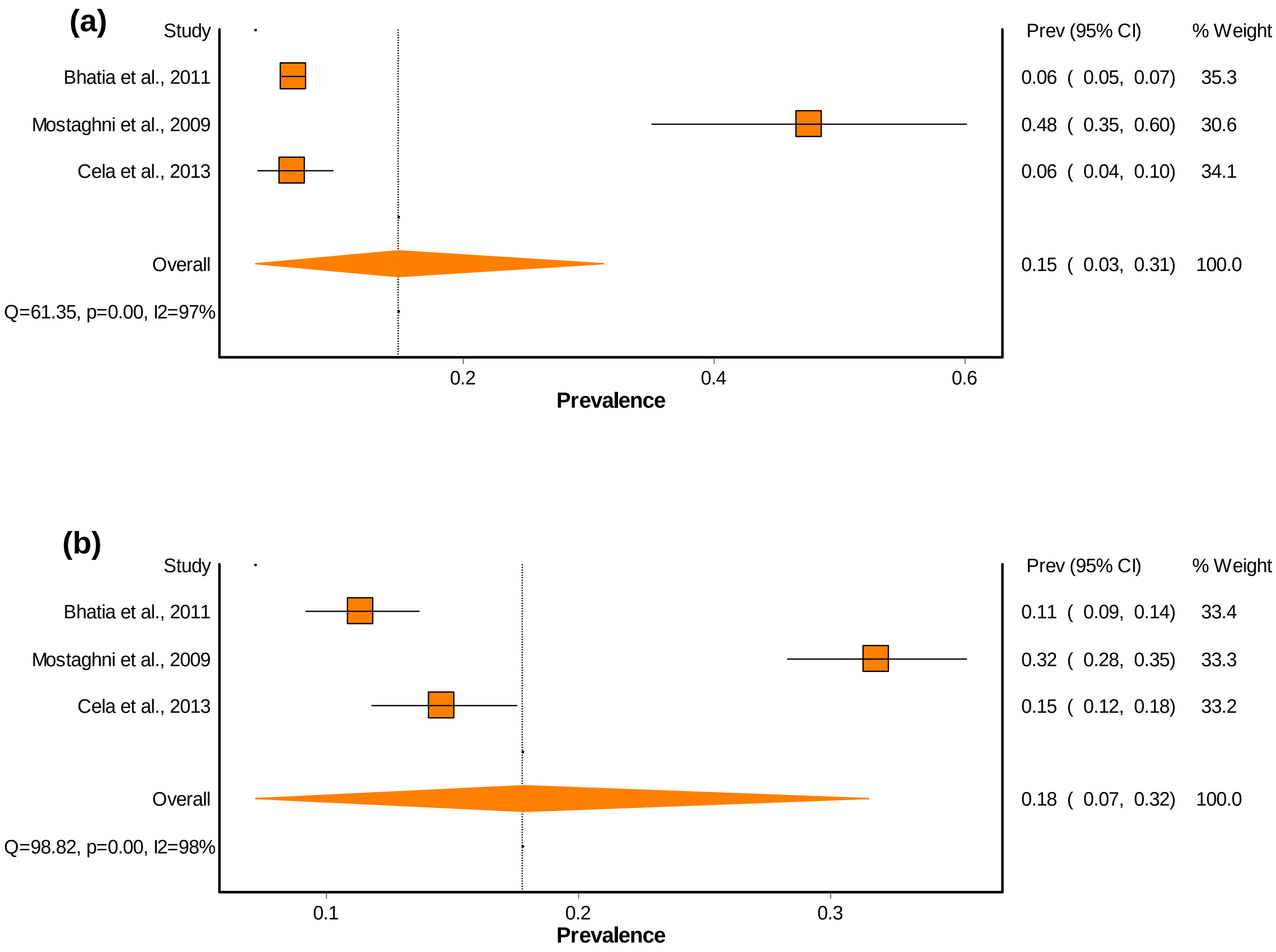

Figure S32. Forest plot showing global prevalence of GORD according to intake of fried food with studies pooled using a random effects model.
(a) Low intake.
(b) High intake.

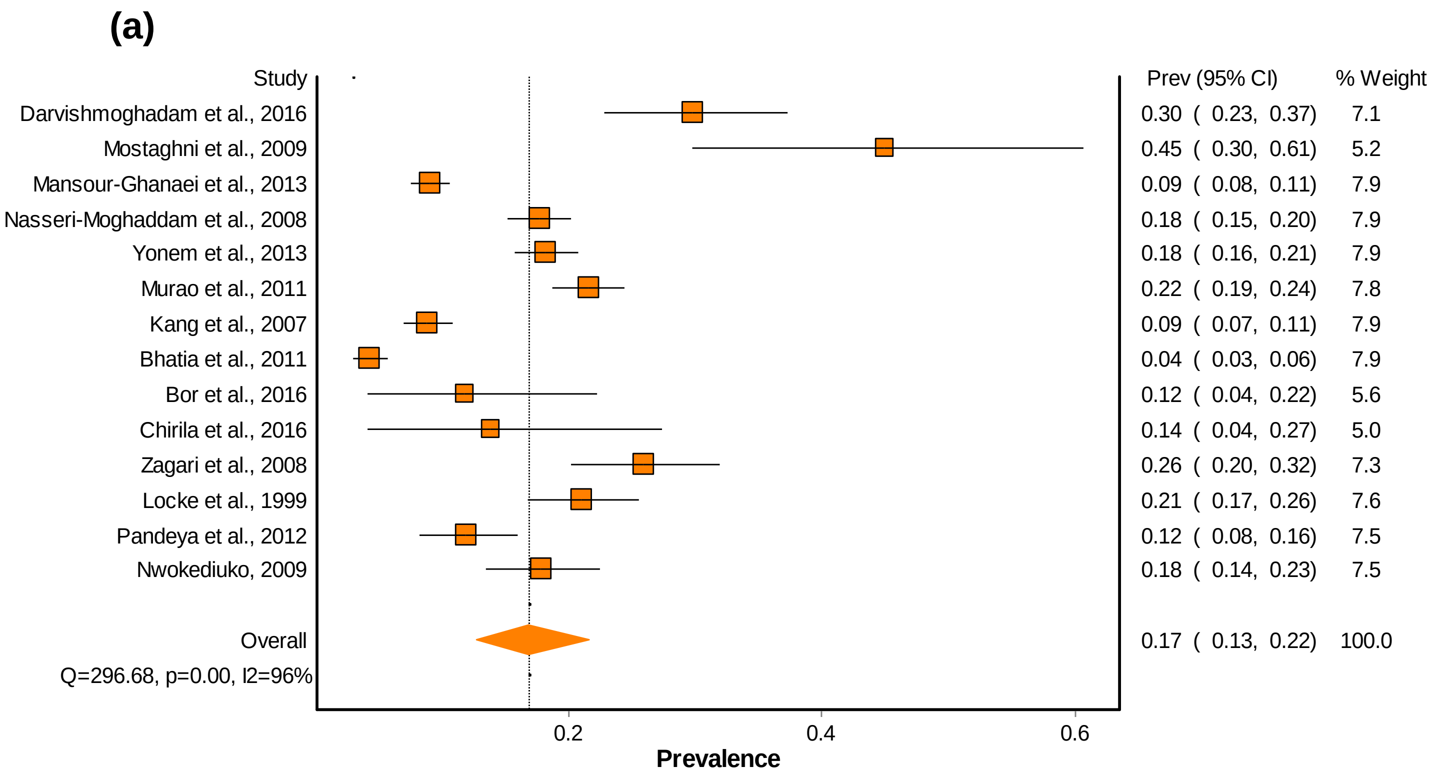

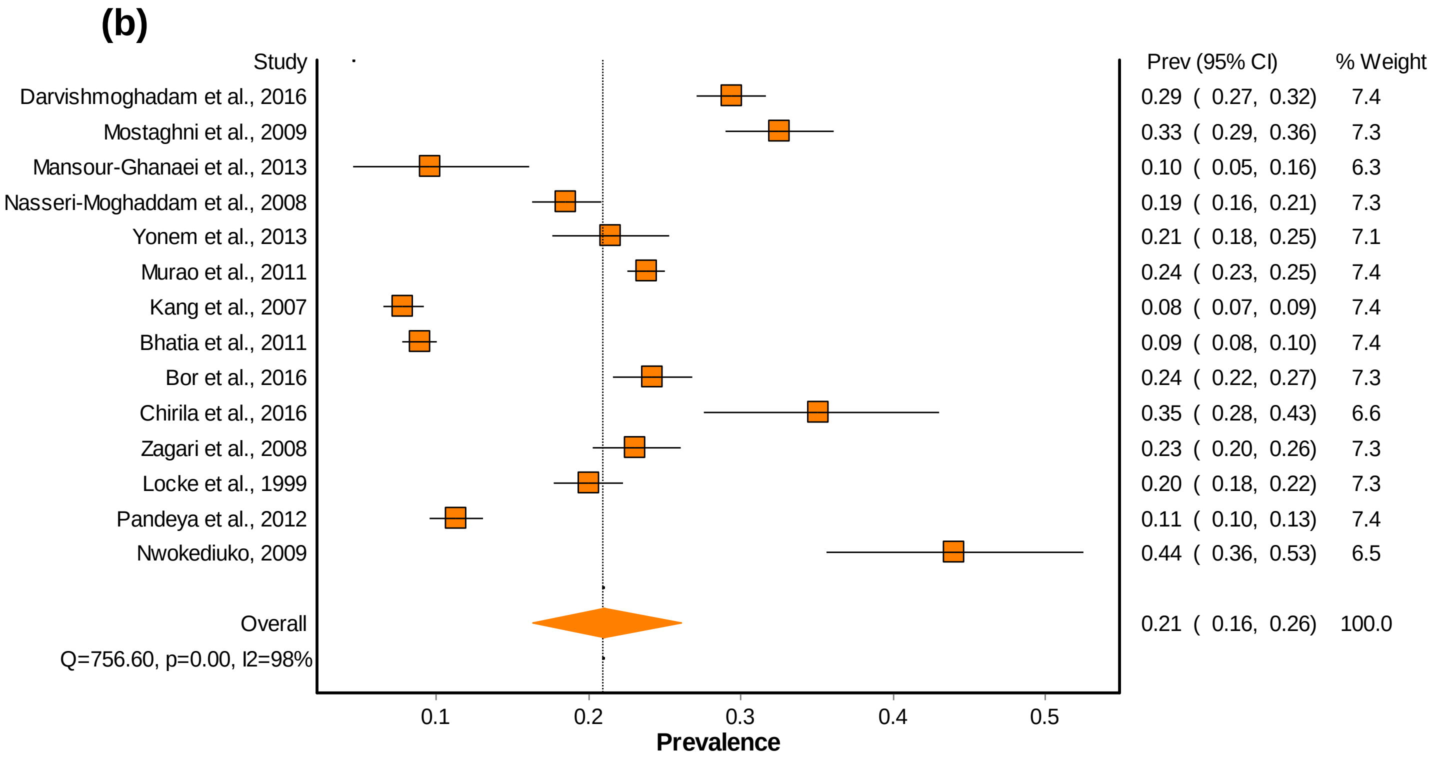

Figure S33. Forest plot showing global prevalence of GORD according to intake of coffee/tea with studies pooled using a random effects model.
(a) Low intake.
(b) High intake.

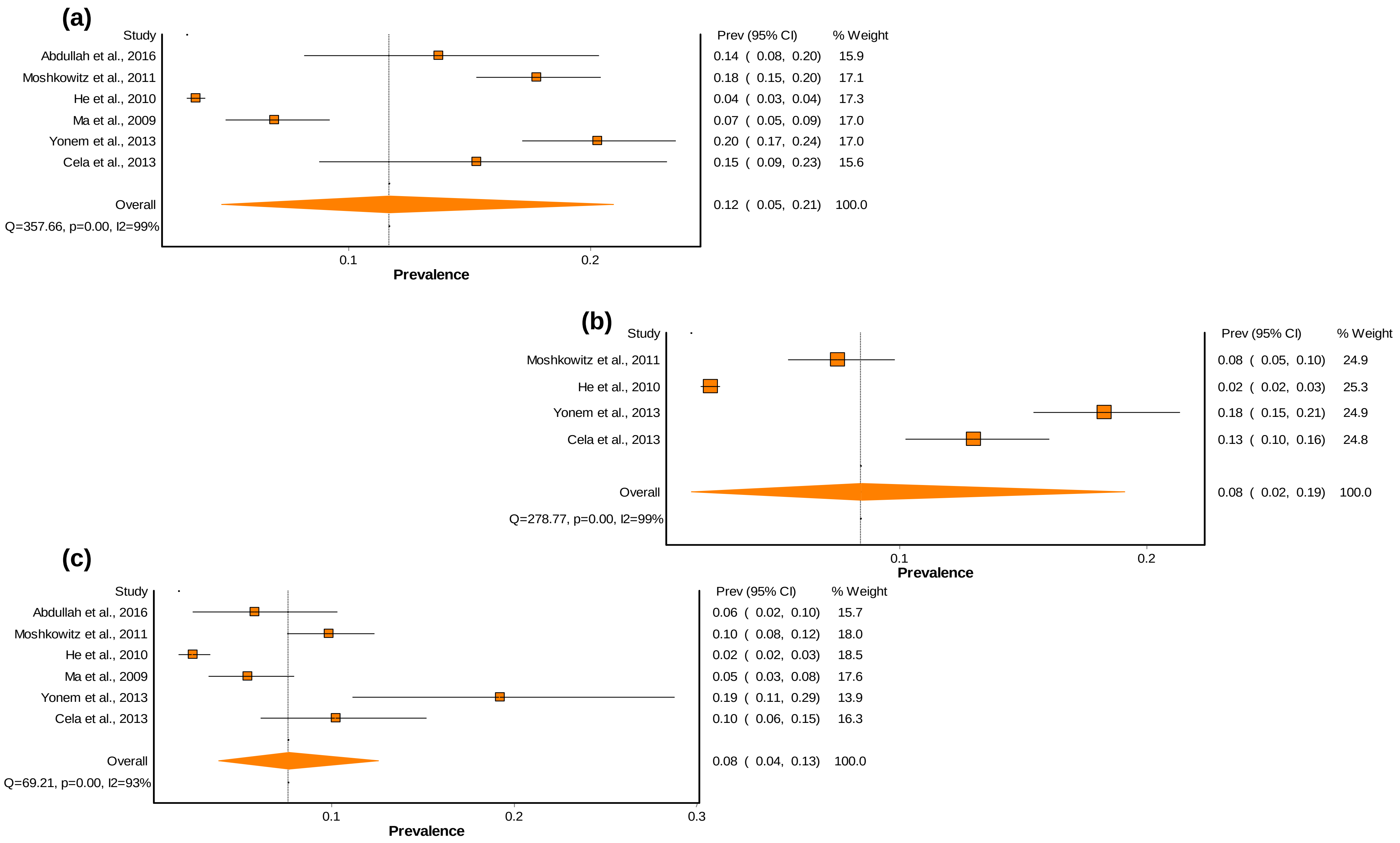

Figure S34. Forest plot showing global prevalence of GORD according to income level with studies pooled using a random effects model.
(a) Low income.
(b) Medium income.
(c) High income.

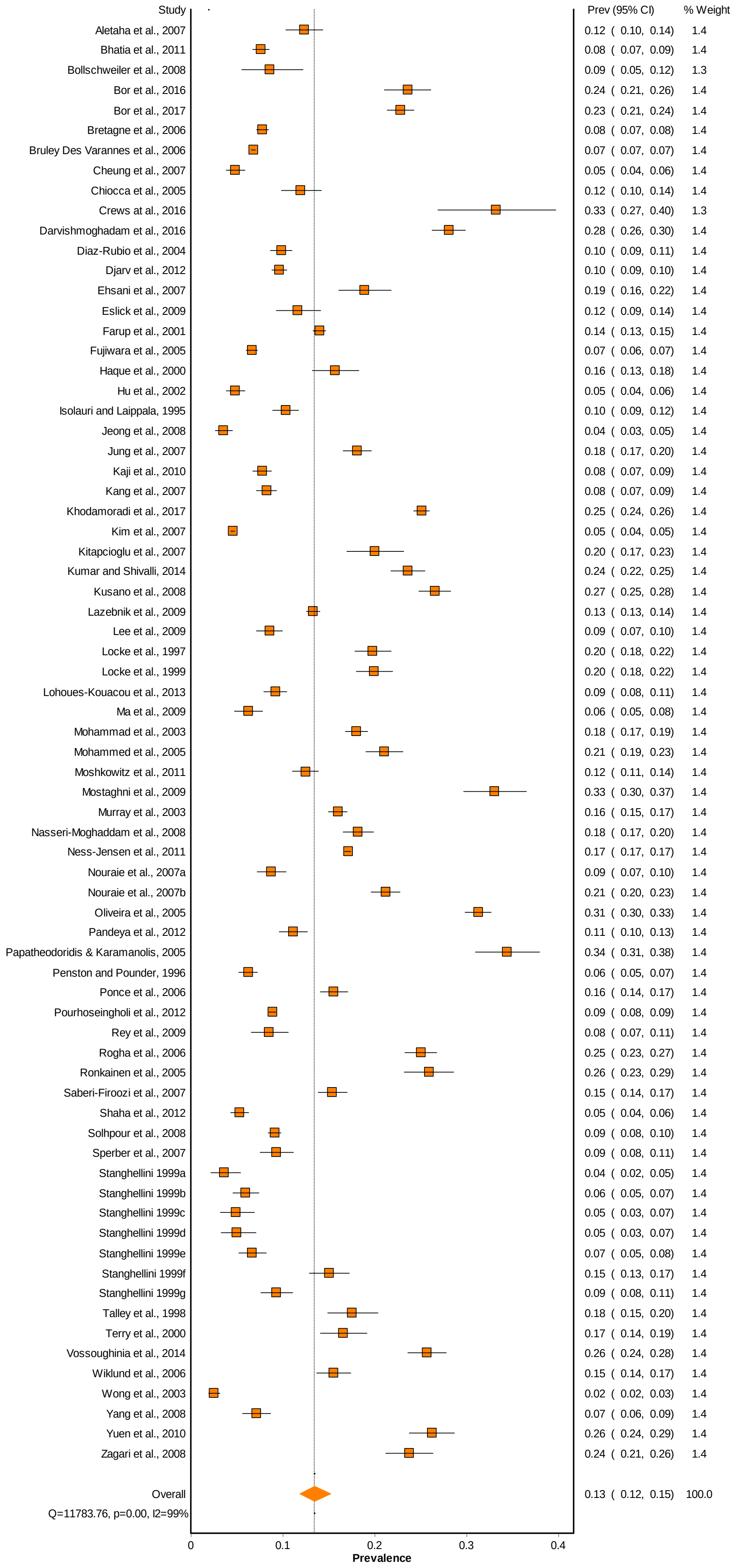

Figure S35. Forest plot showing global prevalence of GORD according to studies using a GORD definition of heartburn and/or acid regurgitation at least once a week with studies pooled using a random effects model.

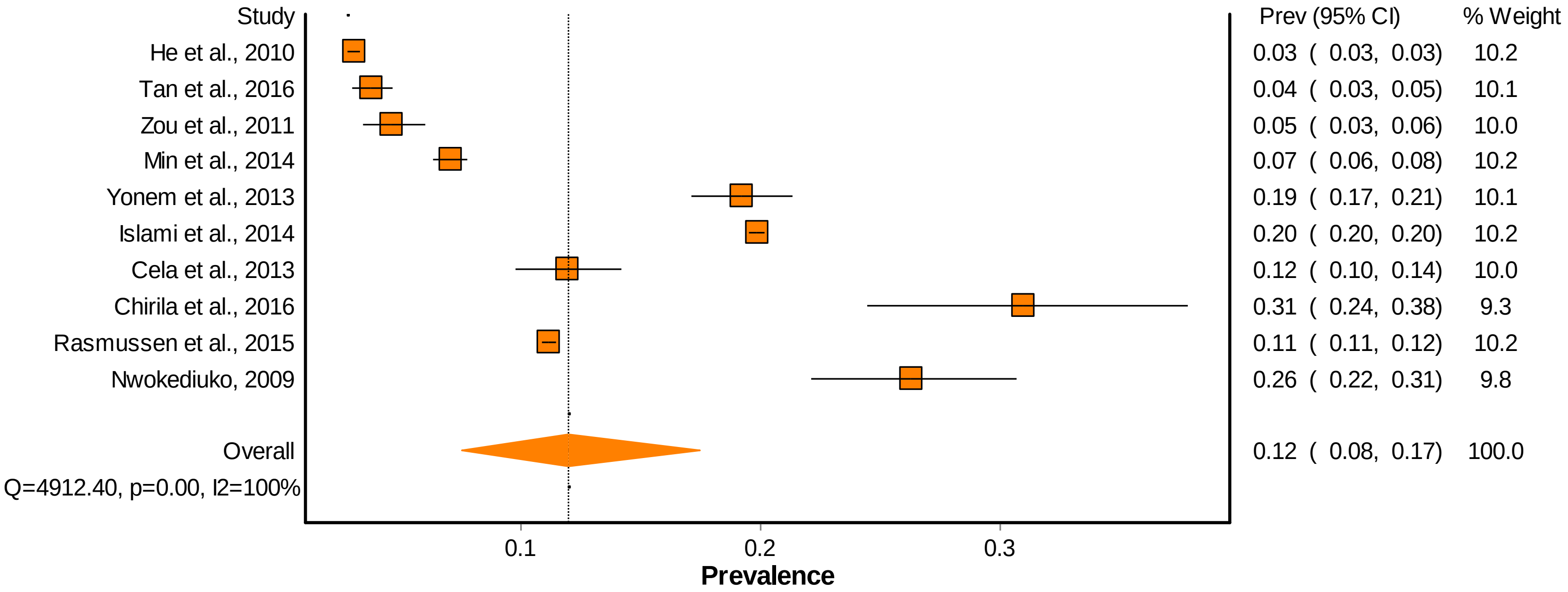

Figure S36. Forest plot showing global prevalence of GORD according to studies using the Montreal definition to define GORD with studies pooled using a random effects model.

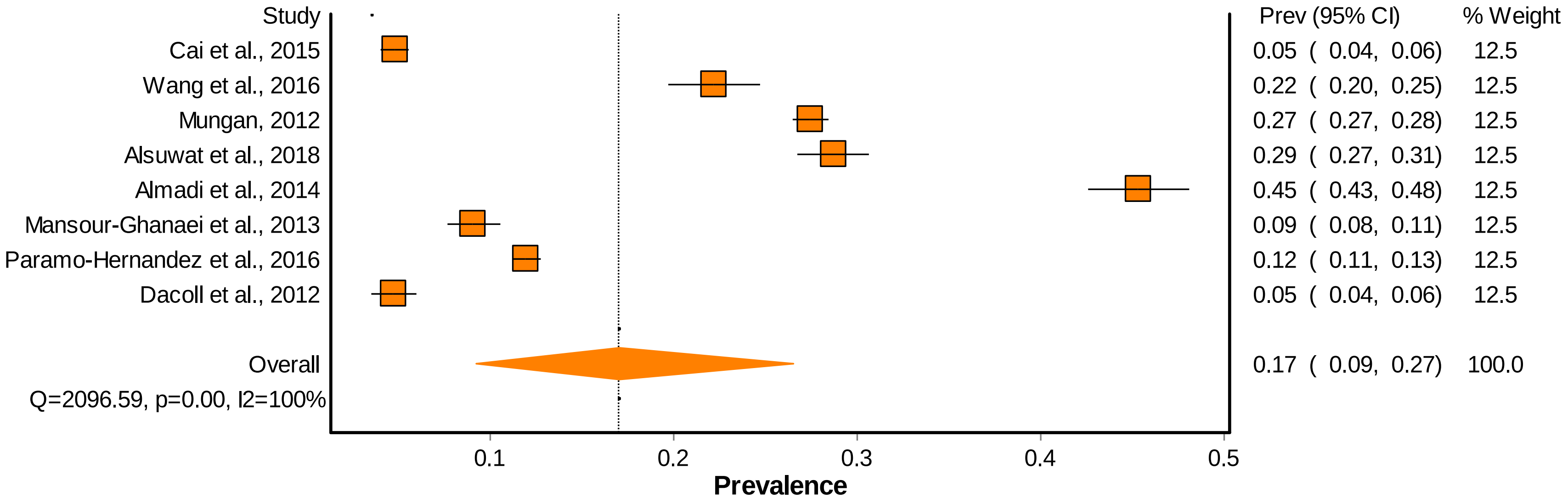

Figure S37. Forest plot showing global prevalence of GORD according to studies using a GERDQ score of at least 8 to define GORD with studies pooled using a random effects model.

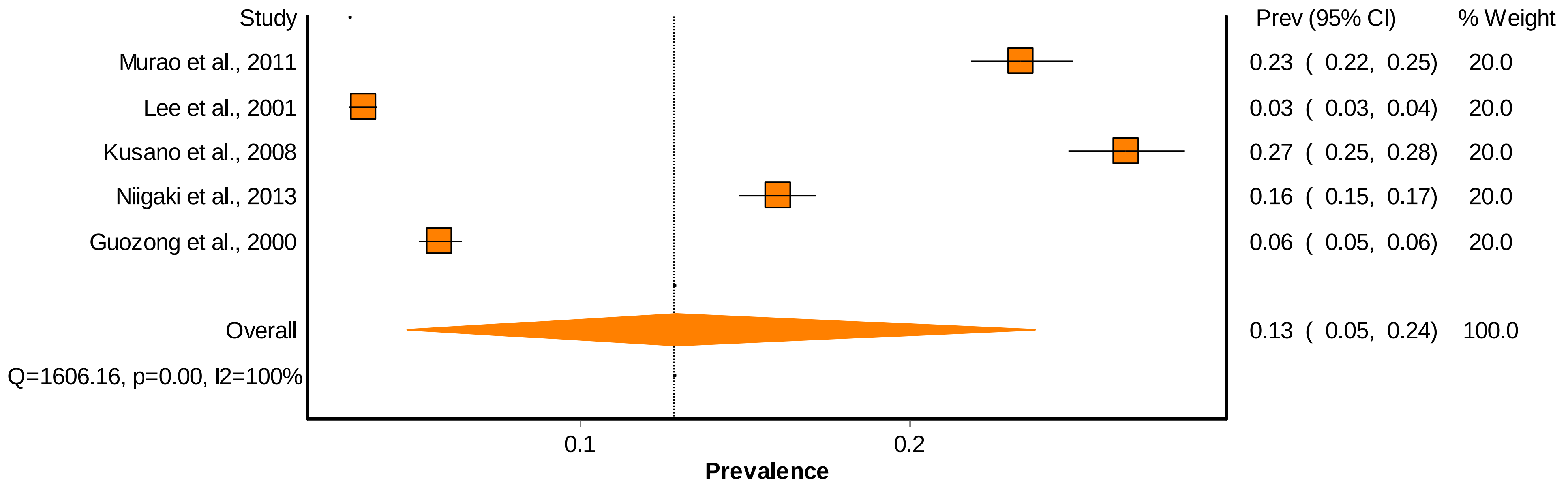

Figure S38. Forest plot showing global prevalence of GORD according to studies using endoscopically proven GORD to define GORD with studies pooled using a random effects model.

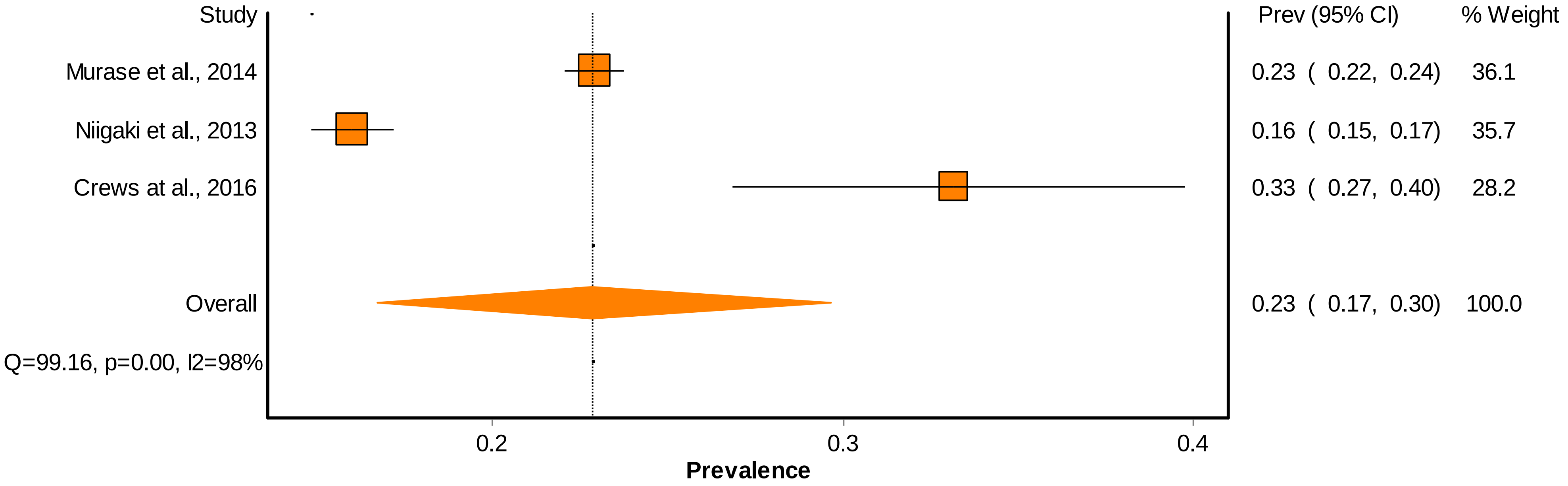

Figure S39. Forest plot showing global prevalence of GORD according to studies defining GORD cases as those currently undergoing treatment for GORD with studies pooled using a random effects model.

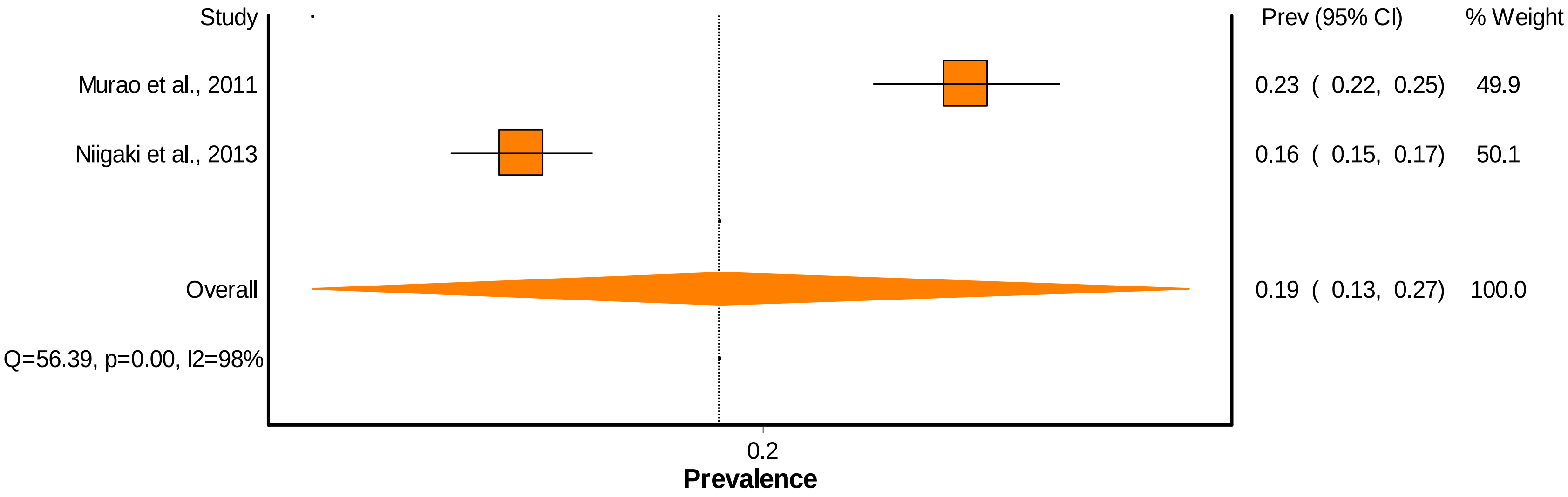

Figure S40. Forest plot showing global prevalence of GORD according to studies using a QUEST score of at least 6 to define GORD with studies pooled using a random effects model.

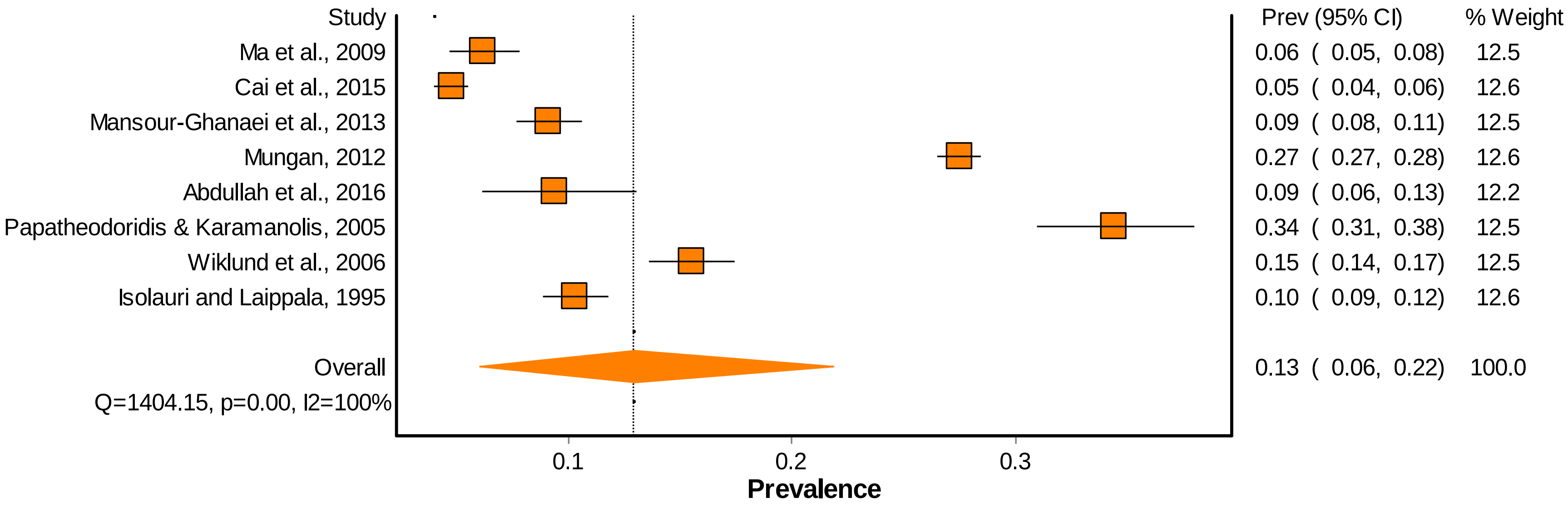

Figure S41. Forest plot showing global prevalence of GORD according to studies investigating GORD symptoms during the previous week with studies pooled using a random effects model.

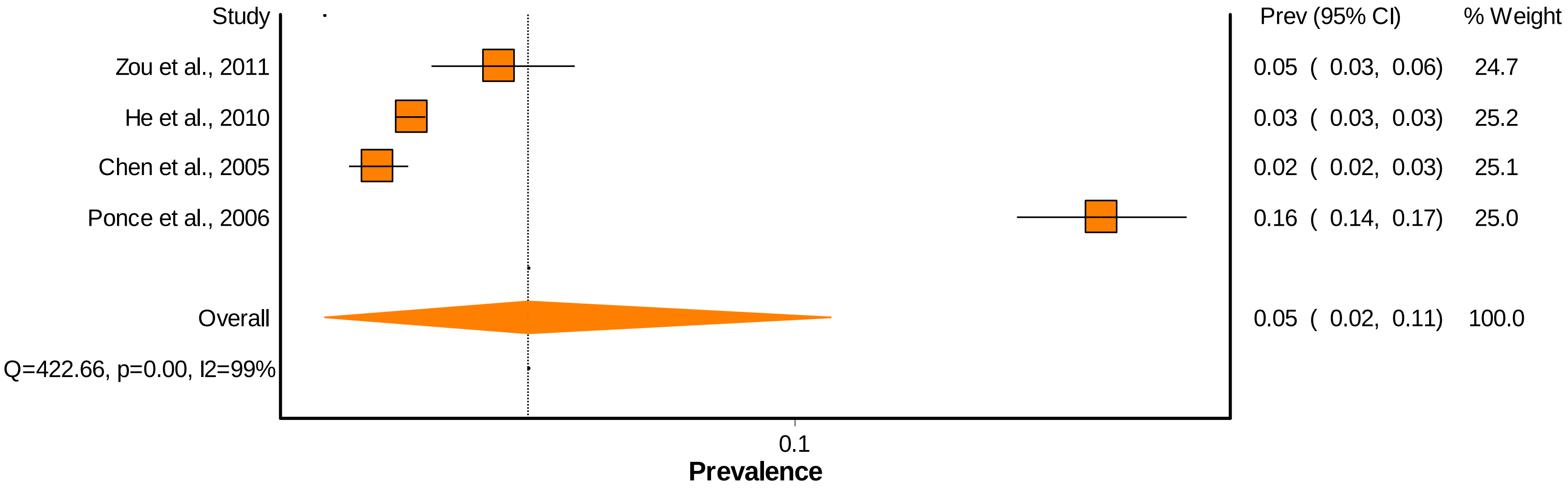

Figure S42. Forest plot showing global prevalence of GORD according to studies investigating GORD symptoms during the previous month with studies pooled using a random effects model.

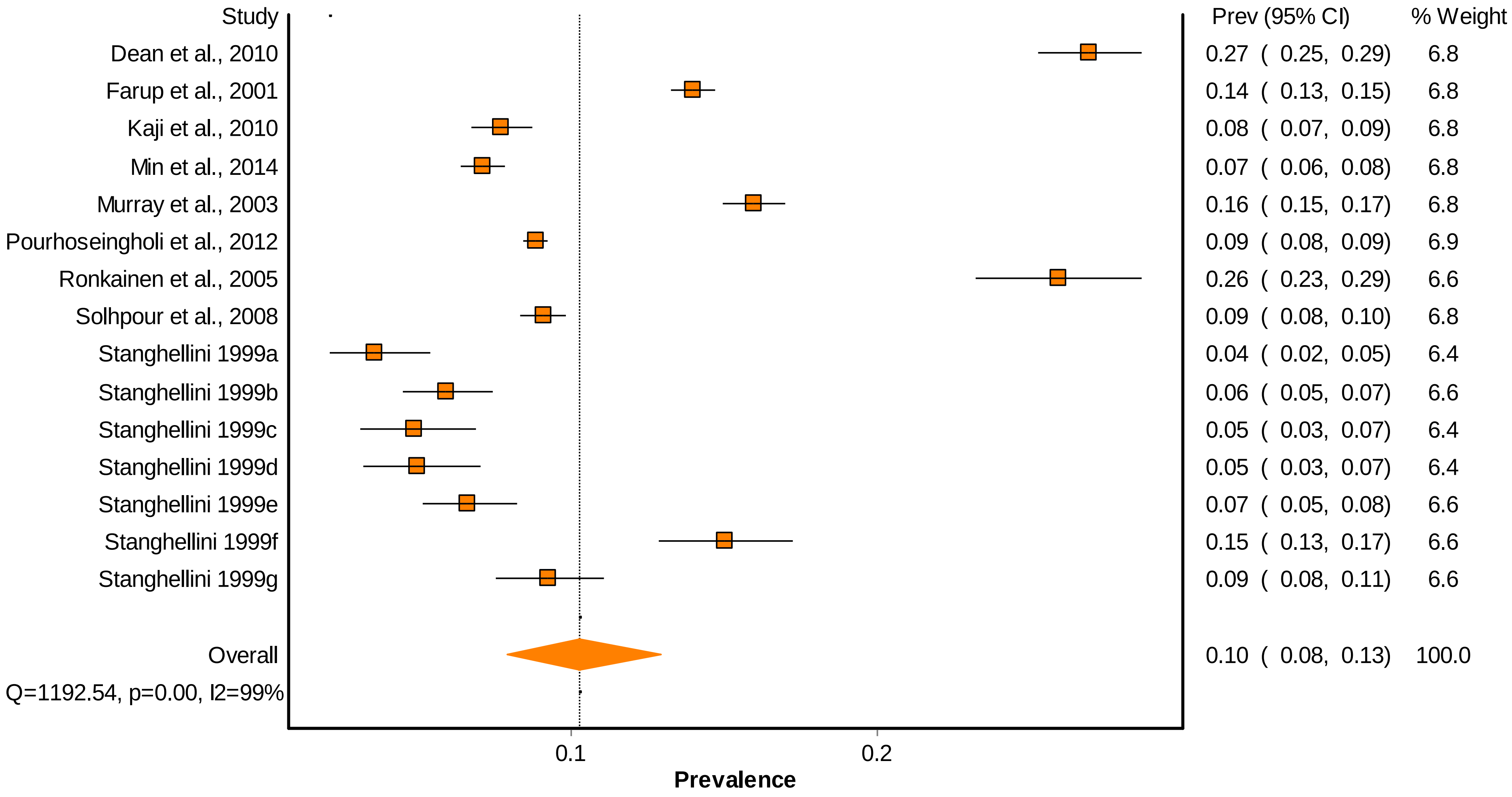

Figure S43. Forest plot showing global prevalence of GORD according to studies investigating GORD symptoms during previous 3 months with studies pooled using a random effects model.

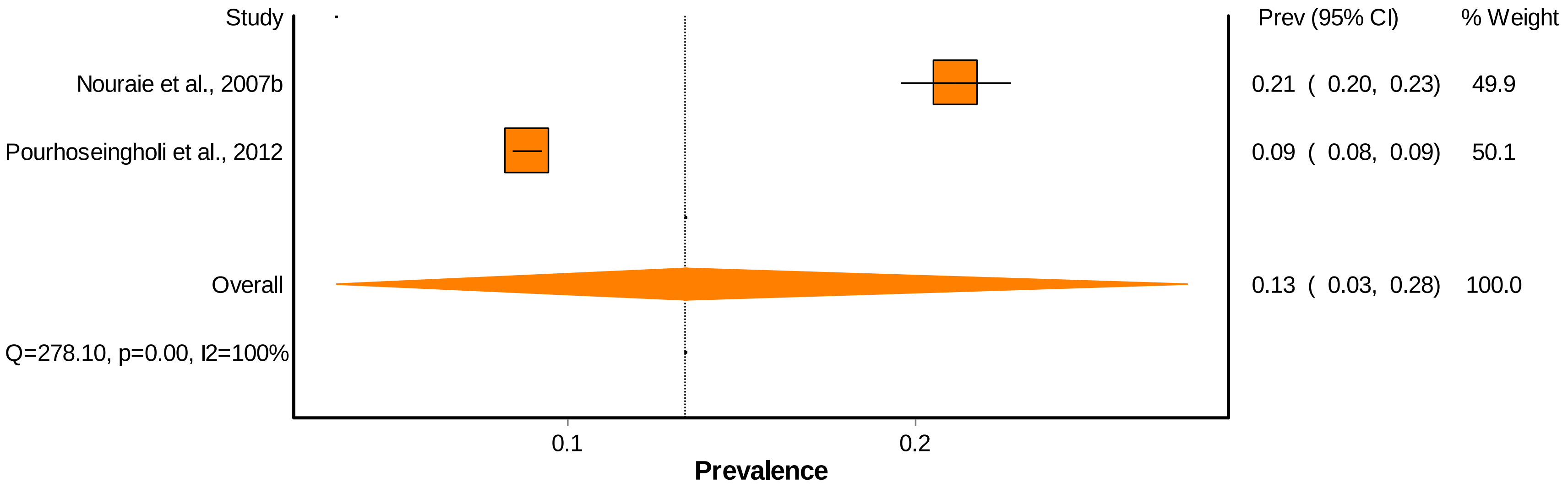

Figure S44. Forest plot showing global prevalence of GORD according to studies investigating GORD symptoms during previous 6 months with studies pooled using a random effects model.

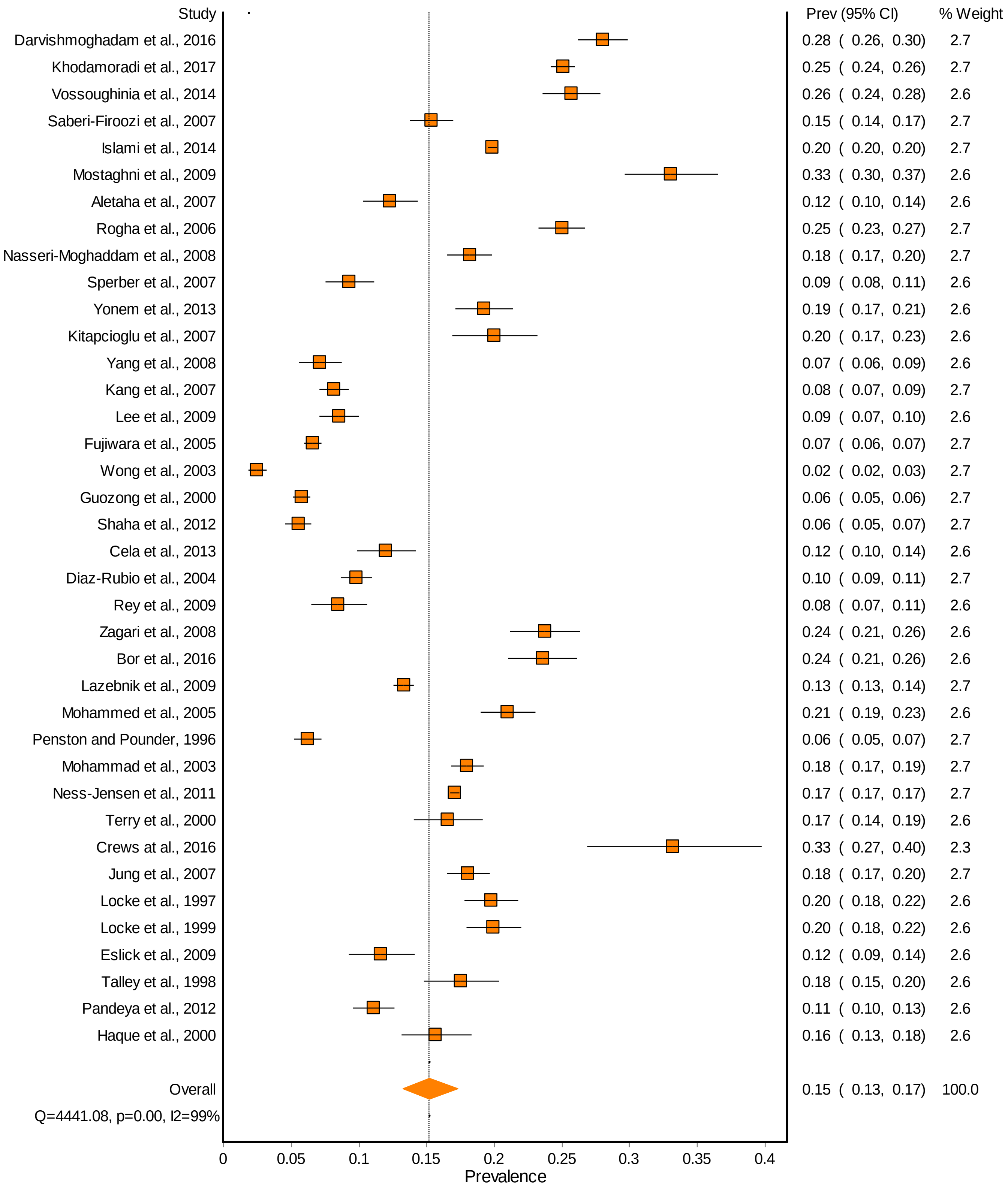

Figure S45. Forest plot showing global prevalence of GORD according to studies investigating GORD symptoms during previous 12 months with studies pooled using a random effects model.

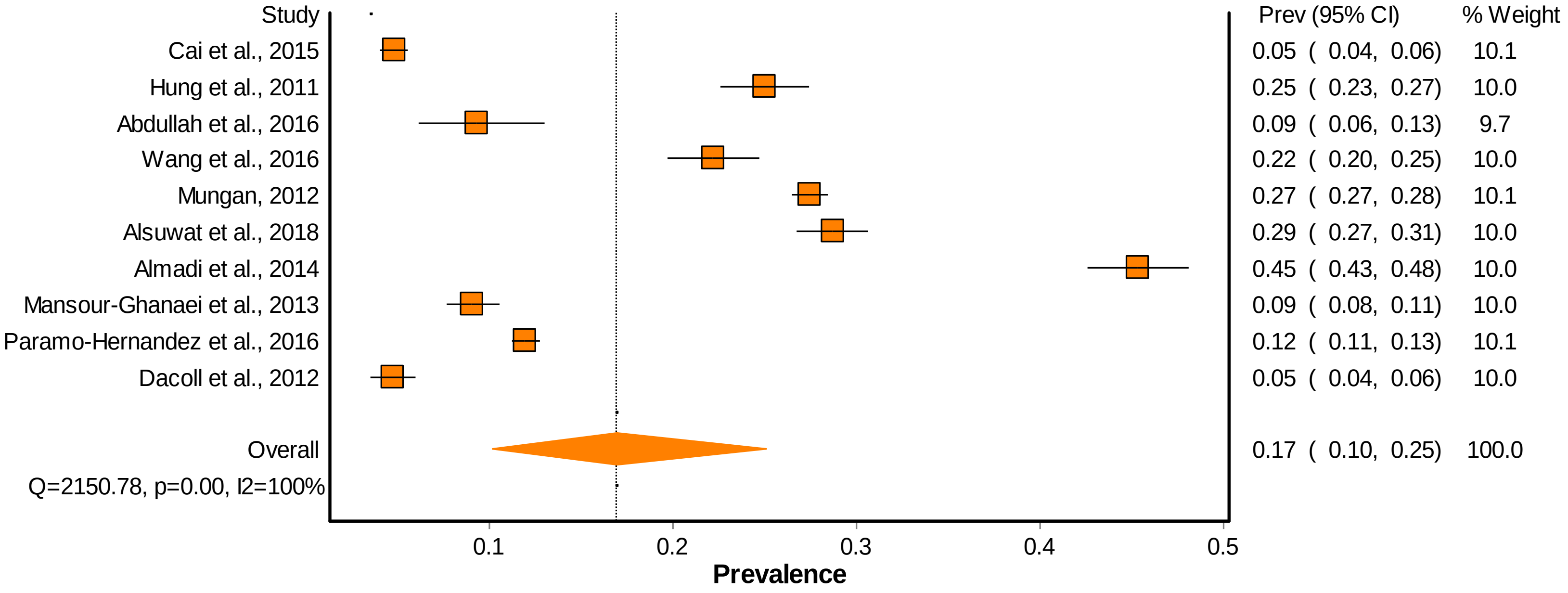

Figure S46. Forest plot showing global prevalence of GORD according to studies using the GERDQ instrument to diagnose GORD with studies pooled using a random effects model.

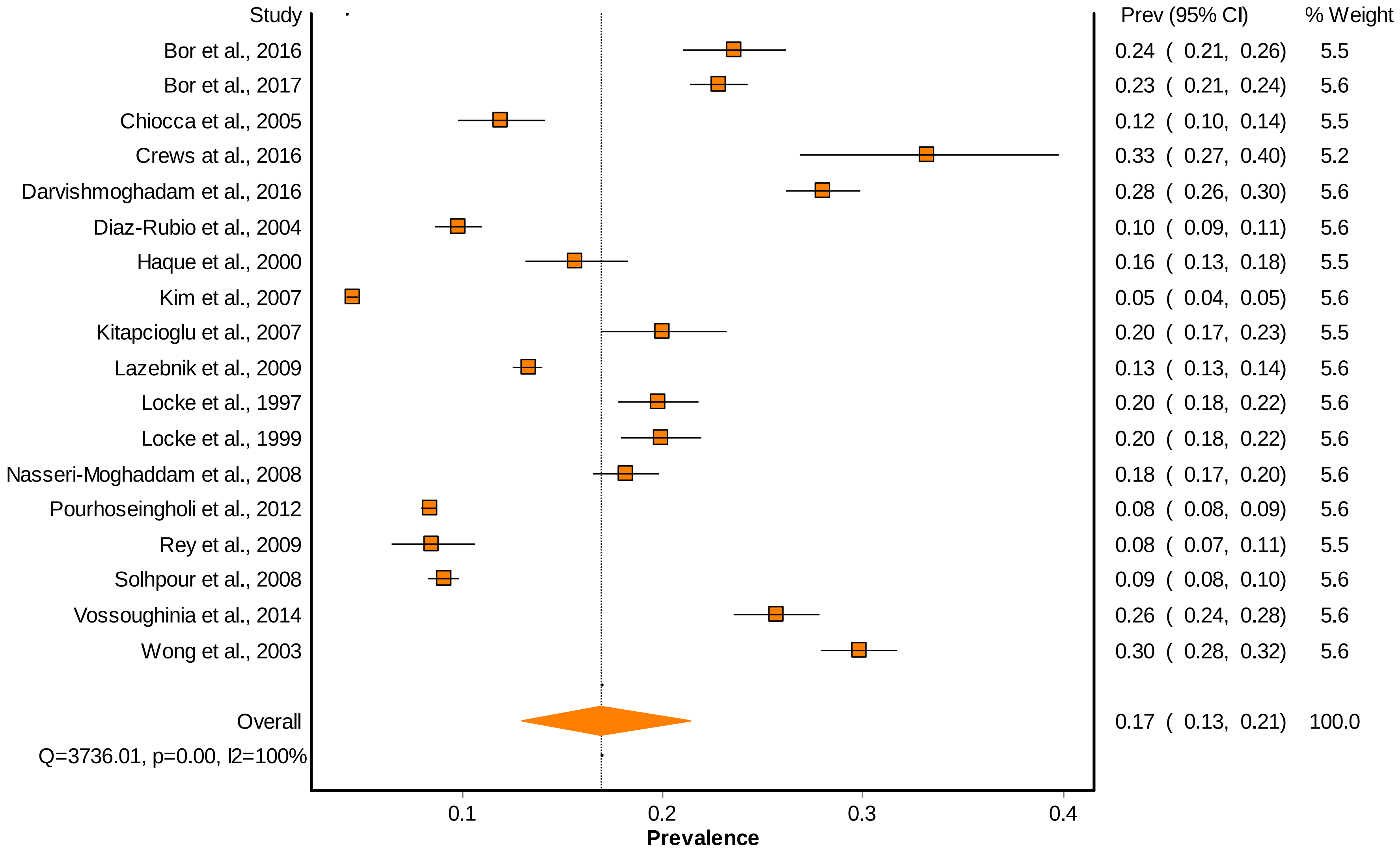

Figure S47. Forest plot showing global prevalence of GORD according to studies using the Mayo Reflux Questionnaire to diagnose GORD with studies pooled using a random effects model.

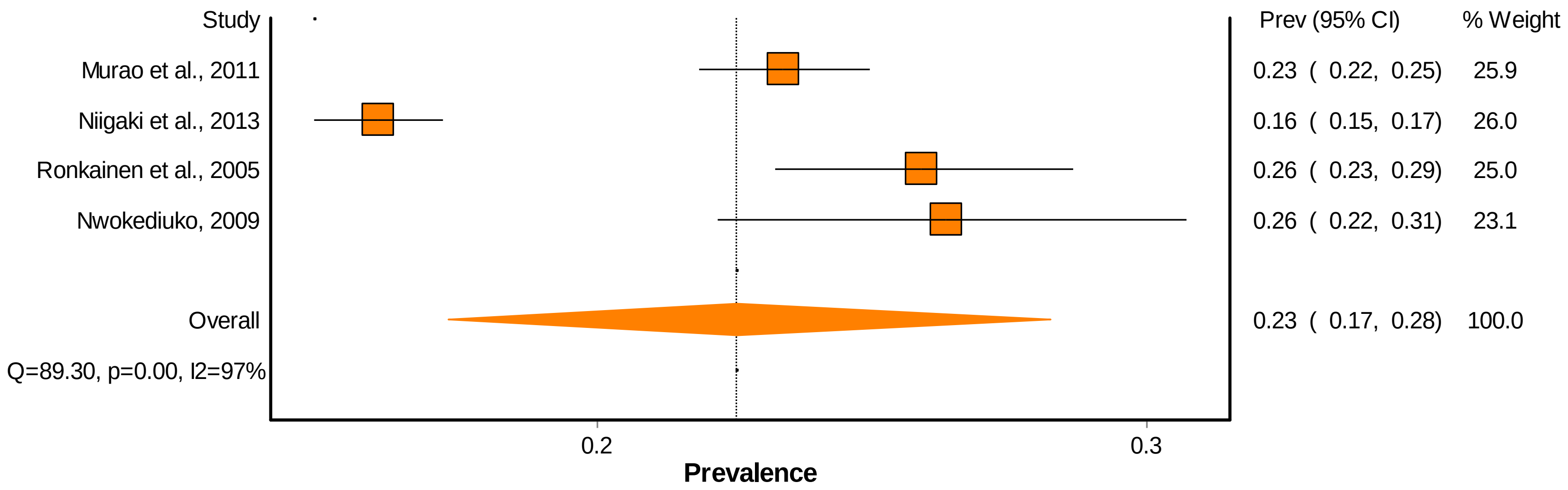

Figure S48. Forest plot showing global prevalence of GORD according to studies using the QUEST instrument to diagnose GORD with studies pooled using a random effects model.

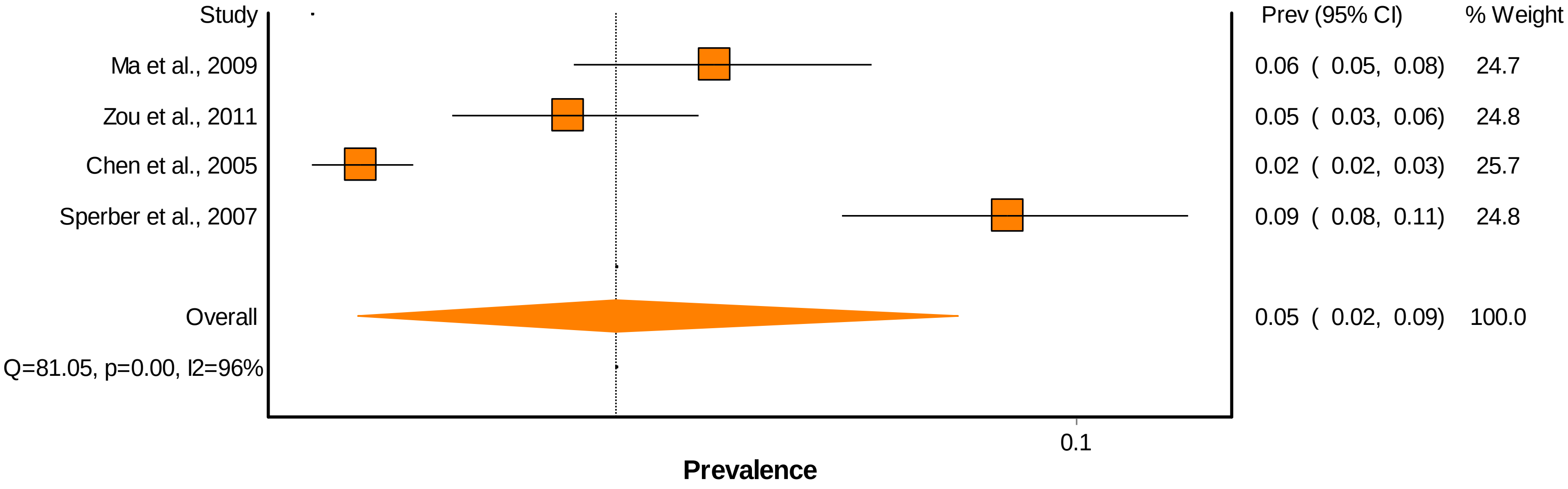

Figure S49. Forest plot showing global prevalence of GORD according to studies using the RDQ instrument to diagnose GORD with studies pooled using a random effects model.

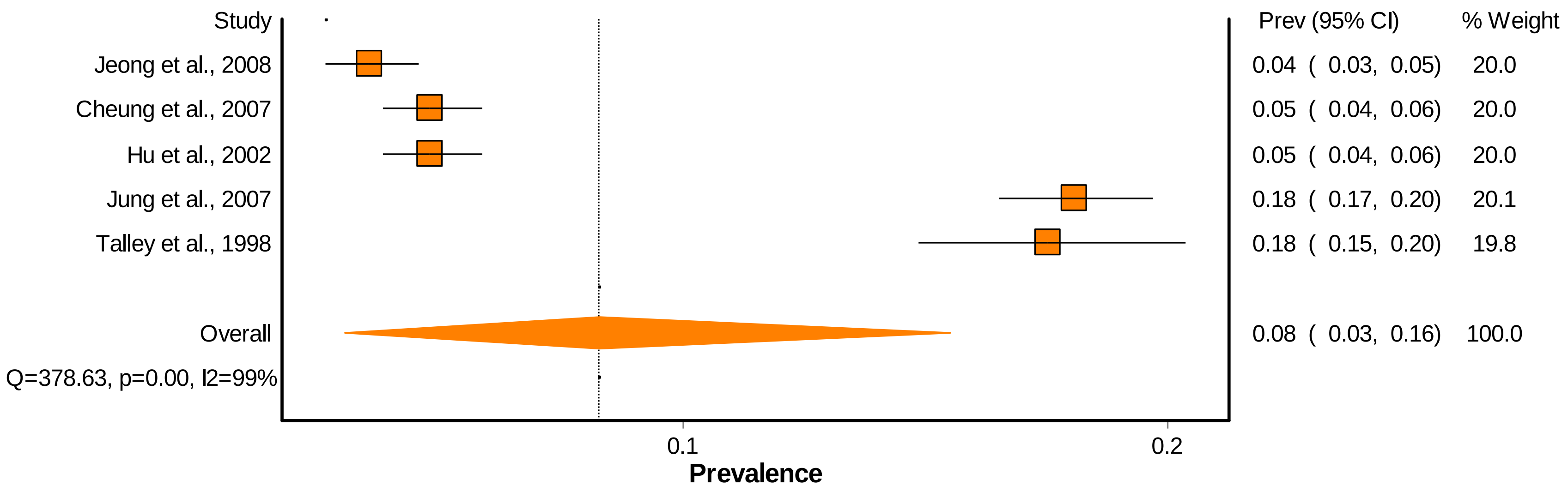

Figure S50. Forest plot showing global prevalence of GORD according to studies using the BDQ instrument to diagnose GORD with studies pooled using a random effects model.

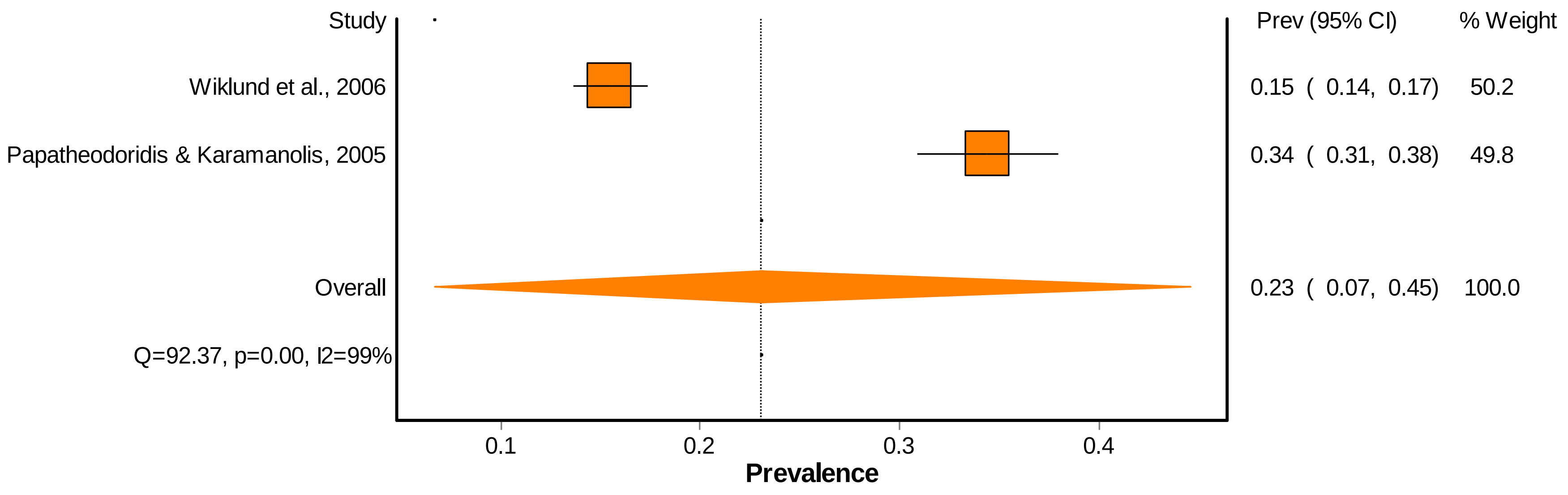

Figure S51. Forest plot showing global prevalence of GORD according to studies using the GSRS instrument to diagnose GORD with studies pooled using a random effects model.

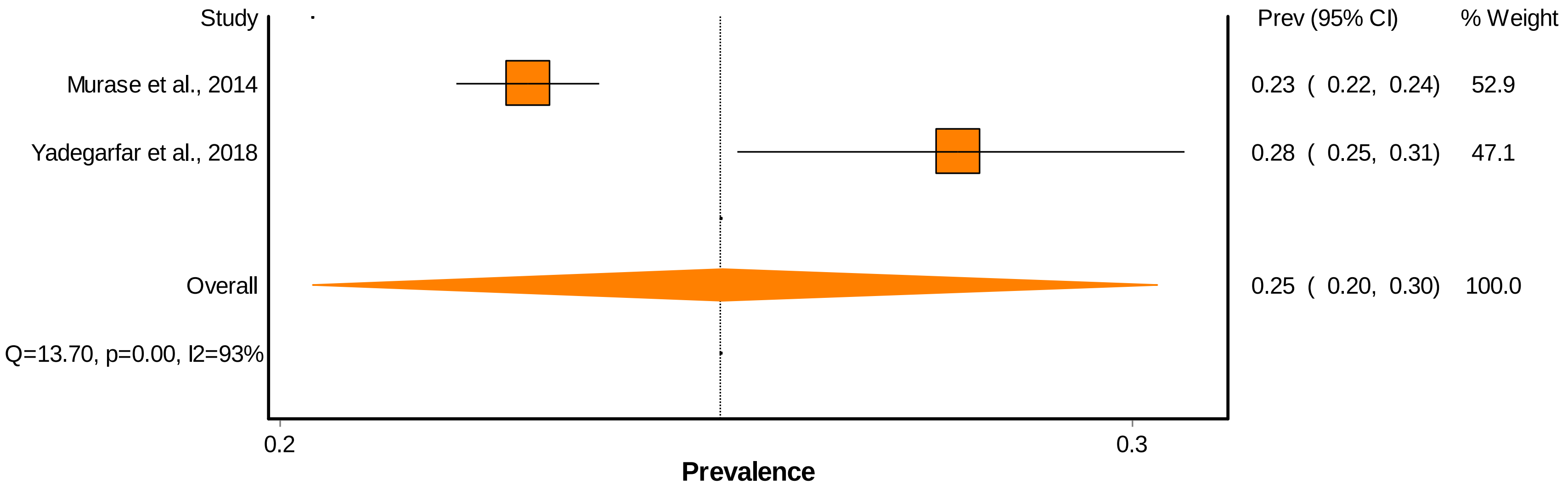

Figure S52. Forest plot showing global prevalence of GORD according to studies using the FSSG instrument to diagnose GORD with studies pooled using a random effects model.

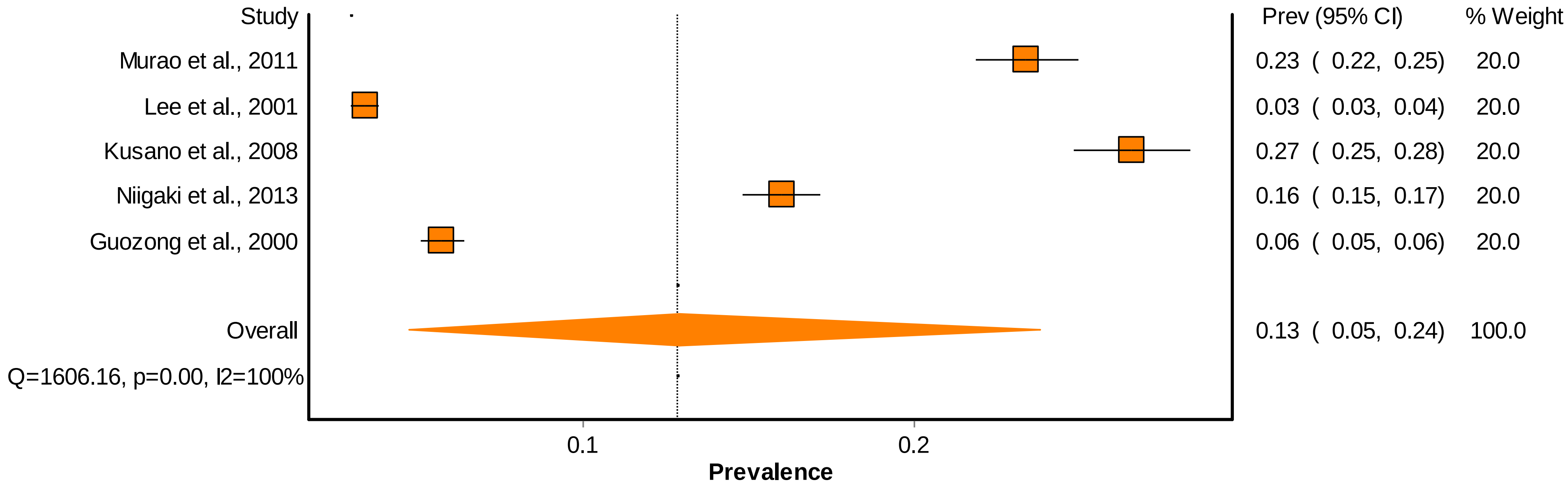

Figure S53. Forest plot showing global prevalence of GORD according to studies using endoscopy to diagnose GORD with studies pooled using a random effects model.

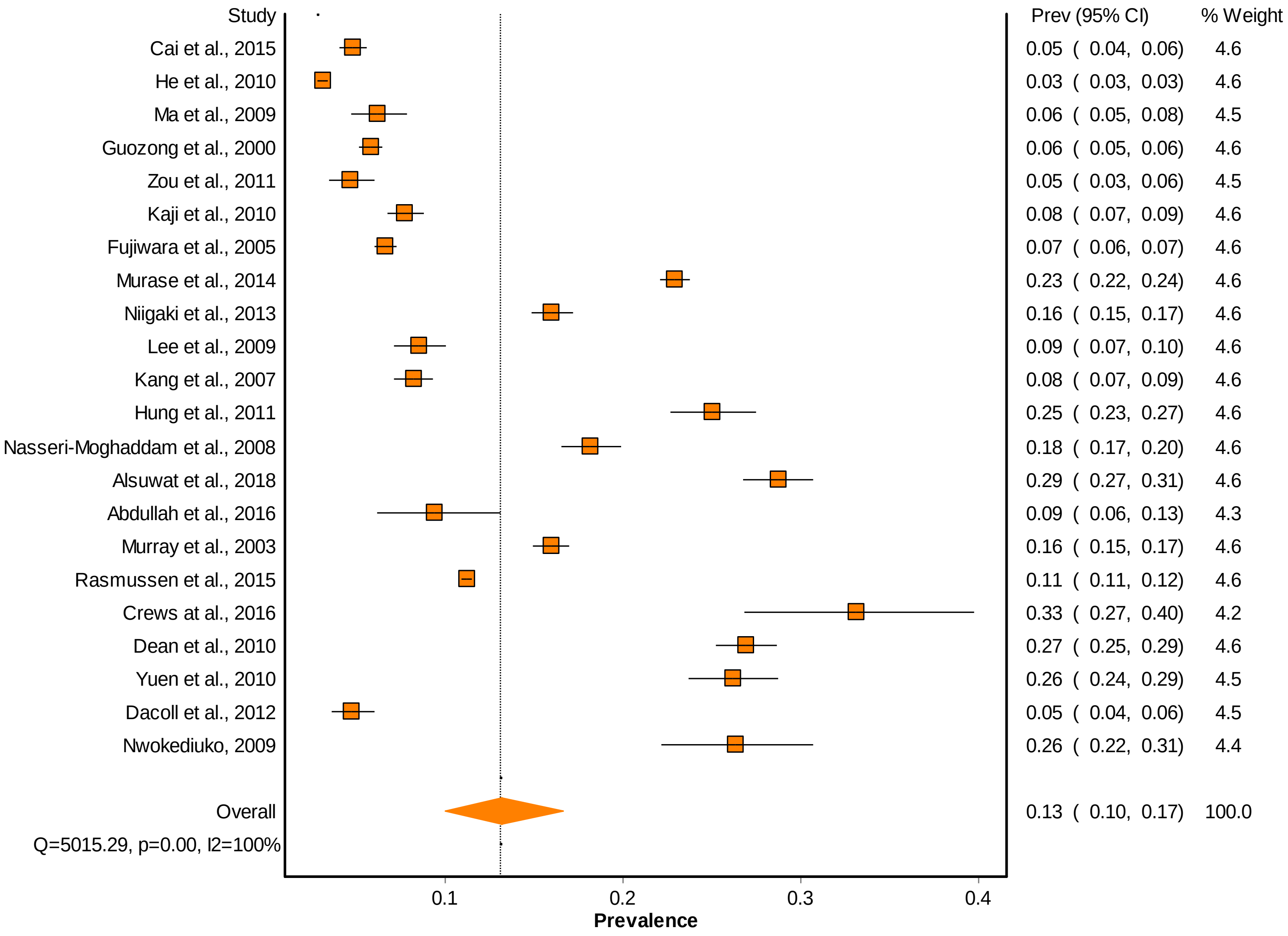

Figure S54. Forest plot showing global prevalence of GORD according to studies using self-completed questionnaires to collect data from subjects for the diagnosis GORD with studies pooled using a random effects model.

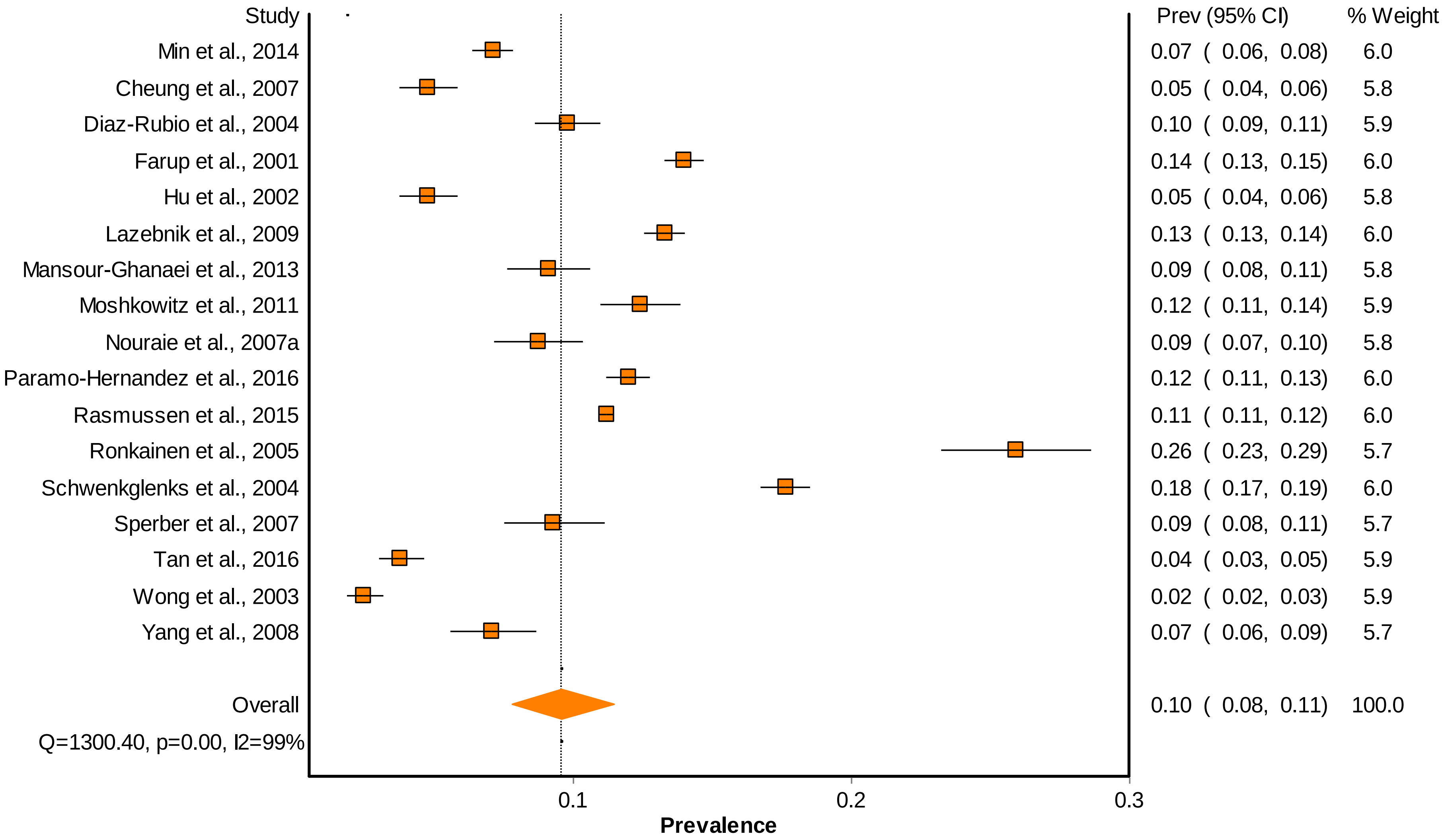

Figure S55. Forest plot showing global prevalence of GORD according to studies using telephone interviews to collect data from subjects for the diagnosis GORD with studies pooled using a random effects model.

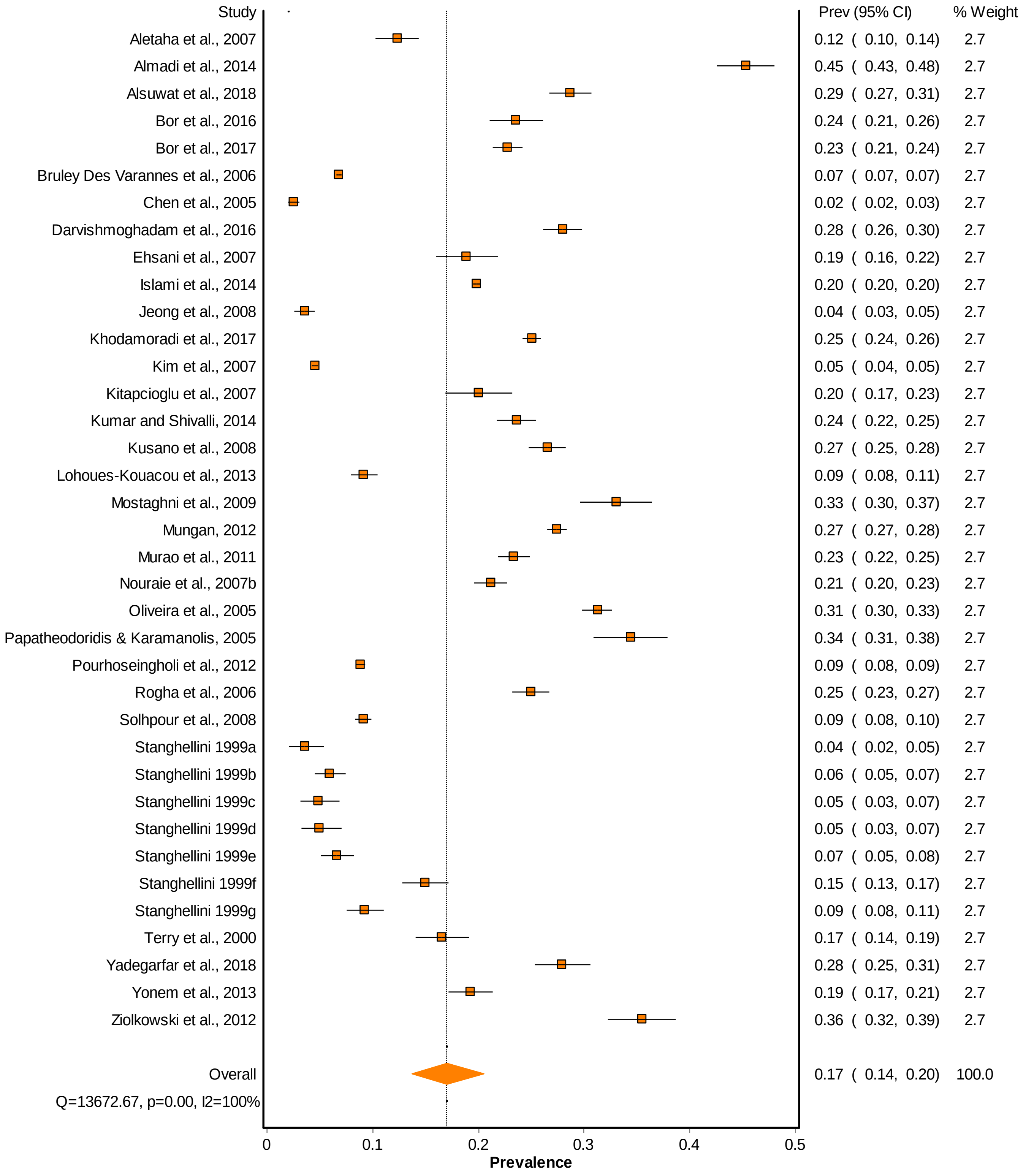

Figure S56. Forest plot showing global prevalence of GORD according to studies using face-to-face interviews to collect data from subjects for the diagnosis GORD with studies pooled using a random effects model.

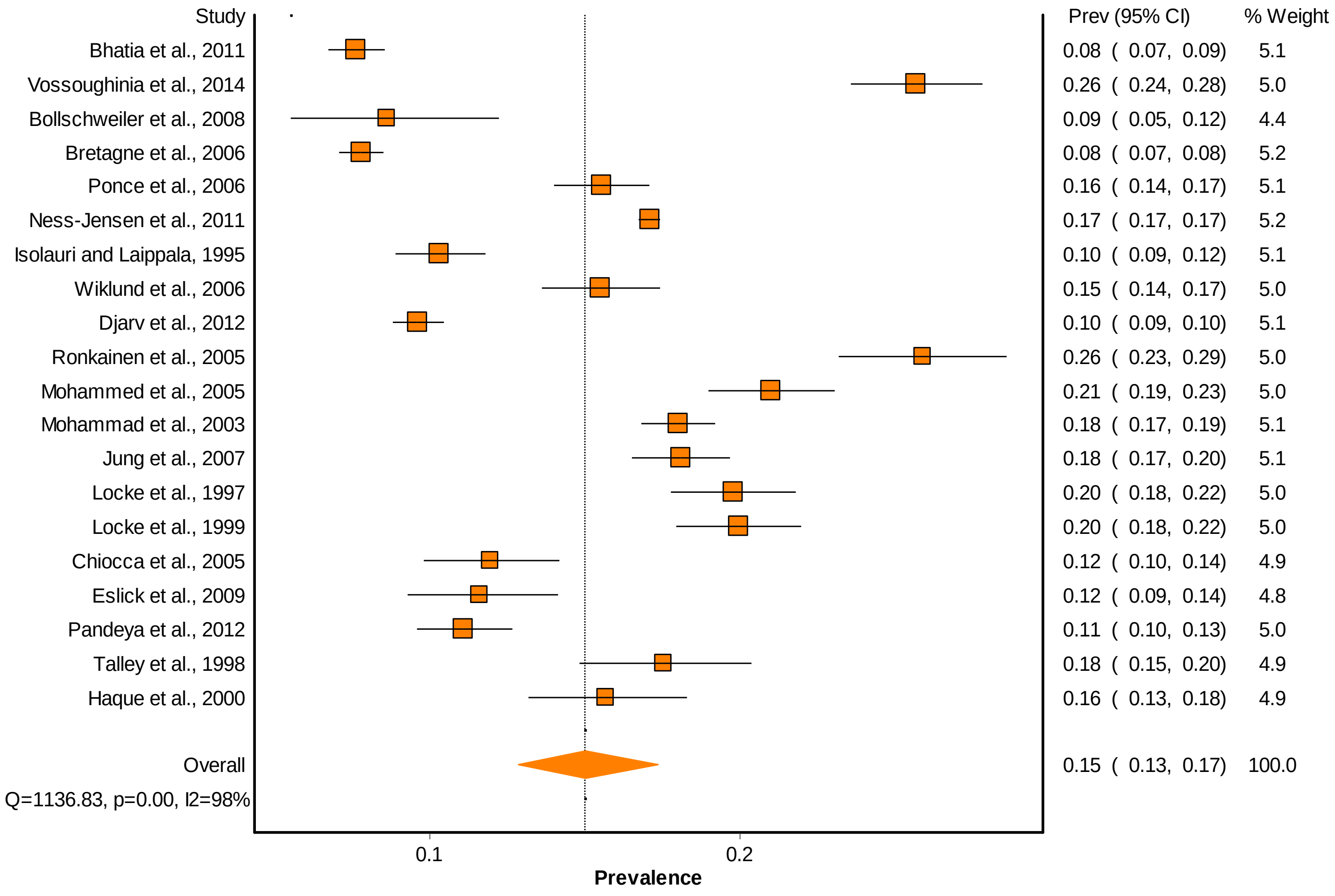

Figure S57. Forest plot showing global prevalence of GORD according to studies using postal questionnaires to collect data from subjects for the diagnosis GORD with studies pooled using a random effects model.

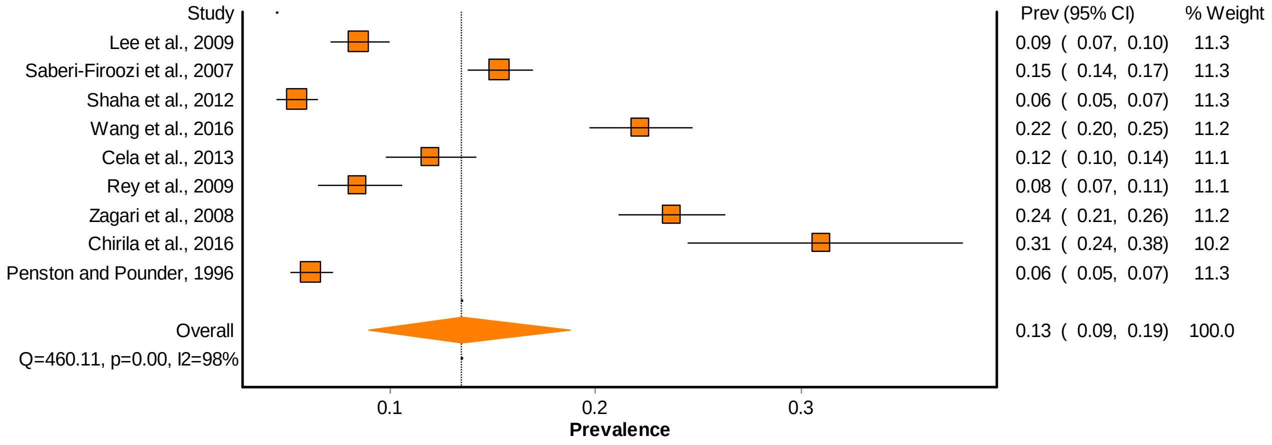

Figure S58. Forest plot showing global prevalence of GORD according to studies using interview-administered questionnaires to collect data from subjects for the diagnosis GORD with studies pooled using a random effects model.
